# Polymethoxyflavones in Citrus Regulate Lipopolysaccharide-Induced Oscillating Decay of Circadian Rhythm Genes by Inhibiting *Nlrp3* Expression

**DOI:** 10.1155/2021/8419415

**Published:** 2021-09-14

**Authors:** Yue Wang, Bo Song, Jiebiao Chen, Jinping Cao, Xian Li, Chongde Sun

**Affiliations:** Laboratory of Fruit Quality Biology, The State Agriculture Ministry Laboratory of Horticultural Plant Growth, Development, and Quality Improvement, Fruit Science Institute, Zhejiang University, Zijingang Campus, Hangzhou 310058, China

## Abstract

The aim of this study is to compare the regulatory abilities of citrus flavonoids on the oscillating expression of circadian genes. Seven varieties of citrus fruits and twenty-five citrus flavonoids were selected and evaluated. *Per2* luciferase bioluminescence report system and serum shock were used to induce circadian gene expression in mouse microglia BV-2 cells. *In vivo* experiments were carried out using C57BL6/J mice to evaluate the regulation of flavonoids on the oscillatory expression of liver biorhythm genes. Lipopolysaccharide was used to interfere the gene oscillating expression. QRT-PCR was performed to detect the expression of circadian rhythm-related genes, including *Clock*, *Bmal1*, *Per1*, *Per2*, *Per3*, *Cry1*, *Cry2*, *Rev-erbα*, *Rev-erbβ*, *Rorα*, *Dbp*, and *Npas2*. The results show that the polymethoxyflavones (PMFs) exerted stronger circadian gene regulatory capability, while the flavonoids containing glycosides showed no biological activity. Also, all tested flavonoids decreased LPS-induced nitric oxide release, but only polymethoxyflavones inhibited circadian rhythm disorder. PMFs inhibited Nlrp3 inflammasome-related genes and proteins, including Nlrp3, IL-1*β*, ASC, and Caspase1, while other flavonoids only affected IL-1*β* and Caspase1 expression. This mechanism was preliminarily verified using the *Nlrp3* inhibitor INF39.

## 1. Introduction

The circadian rhythm, also called the circadian clock, is the intrinsic rhythm of the life activity of organisms determined by the temporal structure, as well as specific genes and proteins [1]. Homeostasis of the circadian rhythm is vital to the organism. The functional patterns of the circadian genes, such as expression and phase, affect basal metabolism [2], organ function [3], and system homeostasis [4]. The biological clock is in a dynamic equilibrium state, and it can be affected by many factors, such as photoperiod [5], inflammatory factors [6], and nutrient intake [7]. An excessive inflammatory response is a common cause of circadian rhythm disturbance [8]. Studying the effects of environmental factors on the circadian rhythm, especially the interaction of ubiquitous inflammatory factors and natural products with the circadian clock, would contribute to revealing the mechanism of circadian clock regulation and guide the consumption of natural products.

Natural products are primary or secondary metabolites of animals, plants, or microorganisms. Flavonoids are an important class of natural products, in which the main structure consists of a C6-C3-C6 ring at its core and substituents at different positions. The substituents of flavonoids mainly include hydroxy groups, methoxy groups, and glycosides [9]. Citrus is one of the daily intake sources of flavonoids. Interestingly, the distribution of flavonoids in citrus is tissue-specific and variety-specific. Flavonoids with high methoxy content (usually equal to or above 4 methoxy moieties), also known as polymethoxyflavones (PMFs), are present in the flavedo of citrus fruits [9], which may be related to their lipophilicity. Flavonoids with low methoxy content, such as hesperidin and narirutin, are detectable in the whole citrus fruit [9]. For example, PMFs exhibit stronger cancer inhibition activities than flavanones [10, 11], while the abilities of flavanones to bind free radicals are stronger than those of PMFs [12]. Exploring the active site and structural features of flavonoids could more effectively explain the mechanism by which flavonoids exert their biological activity. With respect to the regulation of the circadian rhythm, the structure-activity relationship of flavonoids has not yet been tested and elucidated. We hypothesized that flavonoids are structurally specific for the regulation of the oscillating expression of circadian clock genes.

In this study, extractions from 7 varieties of citrus fruits and 25 flavonoid monomers were collected and evaluated using *Per2* luciferase bioluminescence report system and serum shock assay. The results showed that the regulation of circadian genes by flavonoids was structurally specific, and the number of methoxy substitutes was a key variable factor in regulatory capability. PMFs were the main substances in citrus flavonoids that play a role in regulating the oscillation of circadian rhythm genes. Nobiletin also exhibited an *in vivo* inhibitory effect on LPS-induced biological rhythm disorders. The mechanism was preliminarily explored, and the results showed that the regulation of the inflammatory-related circadian disturbance by PMFs was related to the inhibition of the *Nlrp3* gene. To the best of our knowledge, this is the first report to explore and compare the regulation of citrus flavonoids on inflammatory disordered circadian gene expression. The mechanism by which flavonoids inhibit LPS-induced circadian disturbance was also preliminarily revealed in this study.

## 2. Materials and Methods

### 2.1. Chemicals and Materials

Seven citrus fruits including Fuju (FJ, *Citrus reticulata*), Zijinougan (ZJOG, *Citrus reticulata* cv *Suavissima*), Youliang (YL, *Citrus unshiu*), Daetiancheng (DETC, *Citrus sinensis*), Manwengan (MWG, *Citrus hybrids*), Jiweiputaoyou (JWPTY, *Citrus paradisi*), and Mabuwendan (MBWD, *Citrus maxima*) were harvested from Chongqing, China in December 2020. The plant materials were identified by Professor Xiaochun Zhao from China Citrus Research Institute. Fruits free of mechanical damage, diseases, and pests were selected, washed, and stored for later use. The pericarp flavedo layer was separated and then frozen with liquid nitrogen and ground into powder.

Standards of sinensetin, diosmetin, isovitexin, narirutin, and rhoifolin were purchased from Sigma-Aldrich (St. Louis, MO, USA). Standards of isosinensetin, gardenin B, hesperetin, apigenin, naringenin, vitexin, vicenin-2, hesperidin, neohesperidin, diosmin, neodiosmin, didymin, poncirin, naringin, isorhoifolin, eriocitrin, neoeriocitrin, and INF39 were purchased from Shanghai Yuanye Bio-Technology Co., Ltd. (Shanghai, China). Lipopolysaccharides were purchased from Sigma-Aldrich (St. Louis, MO, USA). Nitric oxide assay kit was purchased from Beyotime Biotechnology (Shanghai, China). RPMI-1640 medium, DMEM, fetal calf serum, horse serum, and trypsin-EDTA were purchased from Gibco (Waltham, MA, USA). Trizol reagent was purchased from Invitrogen (Carlsbad, CA, USA). iScript cDNA synthesis kit and SsoFast EvaGreen Supermix was purchased from Bio-Rad (Berkeley, CA, USA). Cell counting kit-8 was purchased from Dojindo (Shanghai, China). Mouse Elisa Nlrp3 kit was purchased from Shanghai Jianglai Industrial. (Shanghai, China). Mouse ELISA ASC kit was purchased from 4A Biotech Co., Ltd. (Beijing, China). pENTR5′-TOPO vector was purchased from Invitrogen (Shanghai, China). pLV7-*Bsd* plasmids were purchased from Hanbio (Shanghai, China). Blasticidin, forskolin, and luciferin were purchased from Solarbio (Beijing, China). Mouse ELISA Caspase1 kit and mouse ELISA IL-1*β* kit were purchased from R&D Systems (Carlsbad, CA, USA). Among the citrus flavonoids tested, nobiletin, tangeretin, and 5-demethylnobiletin were extracted and purified from citrus according to our previous report [13]. Purities of diosmin and neodiosmin were above 95%, and other standards were above 98%. The mouse microglia BV-2 cell line and human embryonic kidney cell line 293T were purchased from the Institute of Cells, Chinese Academy of Science (Shanghai, China).

### 2.2. Separation, Identification, and Quantification of Citrus Flavonoids

The separation of citrus flavonoid compositions was performed according to our previous report [14]. The citrus flavedo powders were subjected to ultrasonic-assisted extraction using ethanol as a solvent, the organic phase was removed by rotary evaporation, and the crude citrus flavonoid extract was further concentrated by vacuum centrifugation.

The identification of citrus flavonoids was carried out following our previous report [9]. The Waters 2695-2996 UPLC system was used for the qualitative and quantitative analysis of citrus flavonoids, with Waters ACQUITY UPLC HSS T3 (1.8 *μ*m, 2.1 × 150 mm). The mobile phases were water (mobile phase A) and acetonitrile (mobile phase B) with a flow rate of 0.4 mL/min. The gradient elution procedure was as follows: 0 to 0.5 min, 2 to 20% B; 0.5 to 2.5 min, 20% B; 2.5 to 8.5 min, 20 to 28% B; 8.5 to 10.5 min, 28 to 50% B; 10.5 to 15 min, 50 to 98% B; 15 to 15.5 min, 98 to 2% B; and 15.5 to 16 min, 2% B. The scanning wavelength range was 200 to 600 nm, and the column temperature was 30°C. The main liquid chromatographic signal peaks in each citrus variety were confirmed by comparison with standards, and the peak area standard curve method was used for quantification.

### 2.3. Construction of Lentiviral Luciferase Reporters

The construction and screening of *Per2* luciferase reporter system were carried out with reference to previous reports [15].

The following primers were used to amplify the *Per2* promoter DNA fragment (562 bp) as follows: forward: 5′-CTC GAG CGG ATT ACC GAG GCT GGT CAC GTC-3′, reverse: 5′-CTC GAG TCC CTT GCT CGG CCC GTC ACT TGG-3′. This fragment was then cloned into the pENTR5′-TOPO vector to form pENTR5′-P(*Per2*). d*Luc* contains the firefly luciferase gene and a C-terminal PEST sequence, which could be used for rapid protein degradation. The d*Luc* fragment was amplified by PCR and cloned into the pENTR/D-TOPO vector to generate pENTR/D-d*Luc*. The two pENTR5′-P(*Per2*) and pENTR/D-d*Luc* plasmids were fused with the lentiviral target vector pLV7-*Bsd* (blasticidin resistance gene), and the cloning enzyme was used for recombination to generate the pLV7-*Bsd*-P(*Per2*)-d*Luc* reporters.

### 2.4. Production of Lentiviral Particles

Human embryonic kidney cell line 293T cells within passage number of 4 were cultured in DMEM at 37°C, 5% CO_2_, 10% FBS, and 1× glutamine. Before transfection, 1 mL 0.001% poly-L-lysine was added to the 6-well plate and incubated at 25°C for 20 min; then, the solution was aspirated and rinsed once with 1× PBS. The cells were seeded in the plate at a concentration of 7.5 × 10^6^ cells per well. When cell proliferation reached confluence of 80%, the culture medium was replaced with fresh culture medium. The lentivirus reporter plasmid DNA (2 *μ*g) was mixed with packaging vectors (1.3 *μ*g GAG/Pol, 0.5 *μ*g REV, and 0.7 *μ*g VSVG). PLV156-CMV-EGFP was transfected with a lentivirus green fluorescent protein (GFP) expression vector and used as a control for transfection and follow-up trials. 100 *μ*L 0.25 M CaCl_2_ diluted with DNase/RNase-free ddH_2_O and 100 *μ*L 2× BSS solution (50 mM BES, 280 mM NaCl, and 1.5 mM Na_2_HPO_4_, pH 6.95) were added to the mixture. After incubation under 25°C for 15 min, the transfected mixture was added drop by drop to 293T cells and the culture plates were gently rotated. The particles were examined under a microscope to confirm formation and incubated overnight. When cell reached confluence of 100%, the medium was removed and changed to fresh DMEM complete medium, and the transfection efficiency was evaluated by observing the expression of EGFP in control cells. The medium containing infectious virus particles was collected, centrifuged at 3000 × g for 5 min, and the supernatant was collected.

### 2.5. Infection of BV-2 Cells

Mouse microglia BV-2 cells were cultured in RPMI-1640 medium added with 10% FBS at 37°C, 5% CO_2_. The cells, at passage number 3, were seeded in 12-well plates at a concentration of 5 × 10^6^ cells per well and cultured overnight. The polybrene was added to the medium containing virus particles to the final polybrene concentration of 5 *μ*g/mL. When BV-2 cells reached confluence of 20%, the medium was replaced with transfection medium and incubated for 24 h. Then, the mixture containing viral particles and polybrene was removed, washed with 1× PBS, and then replaced with a new medium for overnight culture to restore cell growth. When cells reached confluence to 100%, the cells were split and cultured overnight. The medium was removed before the cell confluence went to 50%; fresh medium containing 10 *μ*g/mL of blasticidin was then added to select stably transfected cells. The selection medium was replaced every 2 days, and stable blasticidin-resistant cells were obtained after about 2 times.

### 2.6. Serum Shock Procedures

The serum shock procedure was carried out based on the previous report [16]. For luciferase bioluminescence reporter assay, the stably transfected BV-2 cells were seeded in 96-well plates at a concentration of 1 × 10^4^ cells per well. For circadian gene detection assay, BV-2 cells were seeded in 6-well plates at a concentration of 2 × 10^5^ cells per well and cultured for 96 h before flavonoid treatment. At the serum shock, the medium was exchanged with serum-rich medium (RPMI-1640 containing 50% horse serum), and after 2 h, the medium was replaced with serum-free RPMI-1640 medium. Flavonoid treatments at different concentrations were performed 24 h before serum shock, and flavonoids were dissolved in dimethylsulfoxide (DMSO), for a final DMSO concentration of 0.05% *v*/*v*, and incubated with the cells. Both the serum-rich and the serum-free medium contained the same concentrations of flavonoids as previously treated. The BV-2 cell lines were used at low passage number not exceeding 7. Each experiment was repeated 3 times independently. DMSO was used as a solvent control.

### 2.7. Reporter Cell Bioluminescence

BV-2 cells resistant to blasticidin were seeded in 96-well plates and incubated overnight. Then, the cells were treated with citrus flavonoid extracts and incubated for 24 h before serum shock. Serum shock-treated cells were replaced with the recording media: RPMI-1640 medium, 1× glutamine, 1 *μ*M forskolin, 1 mM luciferin, and 25 mM HEPES, pH 7.4. After replacing the medium, the 96-well plates were covered and sealed with vacuum grease. The microplate reader was used to record the luminescence, counting once every 15 min; the temperature was set to 36°C; and the baseline was the original luminescence value.

### 2.8. Cell Viability Assay

The cell viability assay was performed using a cell counting kit-8 (cck-8) based on our previous report [13]. BV-2 cells at a density of 5 × 10^4^ cells per well were seeded in 96-well plates with 100 *μ*L RPMI-1640 medium and cultured overnight. Different concentrations of DMSO-dissolved flavonoids were added into the medium and incubated with cells for 24 h. Then, the medium was removed and cells were washed twice with PBS. The cck-8 reagent was diluted in FBS-free RPMI-1640 medium and added to the wells. After incubating in the incubator for 1 h, the absorbance at 450 nm and 620 nm was read using a microplate reader. Cell viability was calculated as cell viability (%) = (OD_450(treatment)_ − OD_620(treatment)_)/(OD_450(control)_–OD_620(control)_) × 100%. DMSO was used as a solvent control. Each experiment was repeated 3 times independently.

### 2.9. Animal Assay

Male C57BL/6J mouse of 6-week-old was purchased from Zhejiang Experimental Animal Center. The mice were raised in an SPF environment, with free access to feed and drinking water, the temperature was kept at 22 ± 2°C, and the light-dark cycle is 12 to 12 h. Through preliminary tests (data not shown), the mice were divided into 3 groups with 35 mice in each group, which were the LPS-induced circadian rhythm disturbance model group (the LPS group, intraperitoneal injection of 0.25 mg/kg LPS for 9 d), the nobiletin rescue treatment group for LPS-induced circadian clock disturbance (the LPS+nobiletin group, LPS 0.25 mg/kg+nobiletin 200 mg/kg), and the control group (saline intraperitoneal injection). Mice in the LPS+nobiletin group were treated with LPS after 21 d of continuous of nobiletin intragastric administration; then, the nobiletin treatment was maintained while under LPS treatment. Twenty-four hours after the last LPS injection, mice were sacrificed at the time points of ZT1, ZT5, ZT9, ZT13, ZT17, ZT21, and ZT25 for cervical vertebra dislocation. After dissection, liver tissues were collected for measuring the indicators related to biological rhythm. The animal assays were carried out in accordance with the ethical guidelines of the Animal Experimentation Committee in the College of Medicine, Zhejiang University (animal ethical clearance number: ZJU20200622).

### 2.10. Quantitative Real-Time Polymerase Chain Reaction (qRT-PCR)

The qRT-PCR assay was performed based on our previous report [13]. Total RNA was extracted using Trizol reagent, following cDNA synthesis with an iScript cDNA synthesis kit. SsoFast EvaGreen Supermix was used for the PCR reaction, with the sequences of primers listed in Table S1. Each experiment was repeated 3 times independently.

### 2.11. LPS-Induced Circadian Rhythm Disturbance Treatment and Nitric Oxide (NO) Release Analysis

LPS was used as a positive control drug for inducing circadian rhythm disturbance and NO release analysis. LPS dissolved in DMSO at a concentration of 0.1 *μ*g/mL, which was within the safe concentration as reported in previous studies [8], was incubated with the cells. The order of treatment was as follows: flavonoids were incubated with the cells for 12 h; then, LPS was added, and LPS and flavonoids were coincubated with the cells another 12 h before serum shock. NO content was examined using the detection kit according to the manufacturer's instructions. DMSO was used as the negative control. Each experiment was repeated 3 times independently.

### 2.12. Enzyme-Linked Immunosorbent Assay (ELISA)

The ELISA was used to detect the protein concentration of Nlrp3, ASC, IL-1*β*, and Caspase1 and was carried out based on our previous report [13]. BV-2 cells at a density of 5 × 10^4^ cells per well were plated onto 96-well plates and cultured overnight for cell adhesion. Flavonoids were added and incubated for 24 h before detection. LPS was added 12 h before detection. DMSO was used as the solvent control. Each experiment was repeated 3 times independently.

### 2.13. Verification Assay Using Nlrp3 Inhibitor INF39

A verification assay was carried out with the *Nlrp3* inhibitor INF39 according to a previous report [17]. INF39 (dissolved in DMSO with a final concentration of 10 *μ*M) at a safe dose range (data not shown) was added to the BV-2 cells simultaneously with flavonoids for 24 h before serum shock. Three independent experiments were performed with three parallel wells in each experiment.

### 2.14. Statistics

Resulting data were expressed as the mean ± standard deviation. Statistical analyses were carried out using SPSS 19.0 software (IBM, Armonk, NY, USA). Data was analyzed for significance and calculated using one-way analysis of variance (ANOVA), followed by Student's *t*-test. Statistical significance was set at *p* < 0.05.

## 3. Results

### 3.1. Regulation of Citrus Flavonoid Extracts on Circadian Rhythm of BV-2 Cells

**Figure 1:**
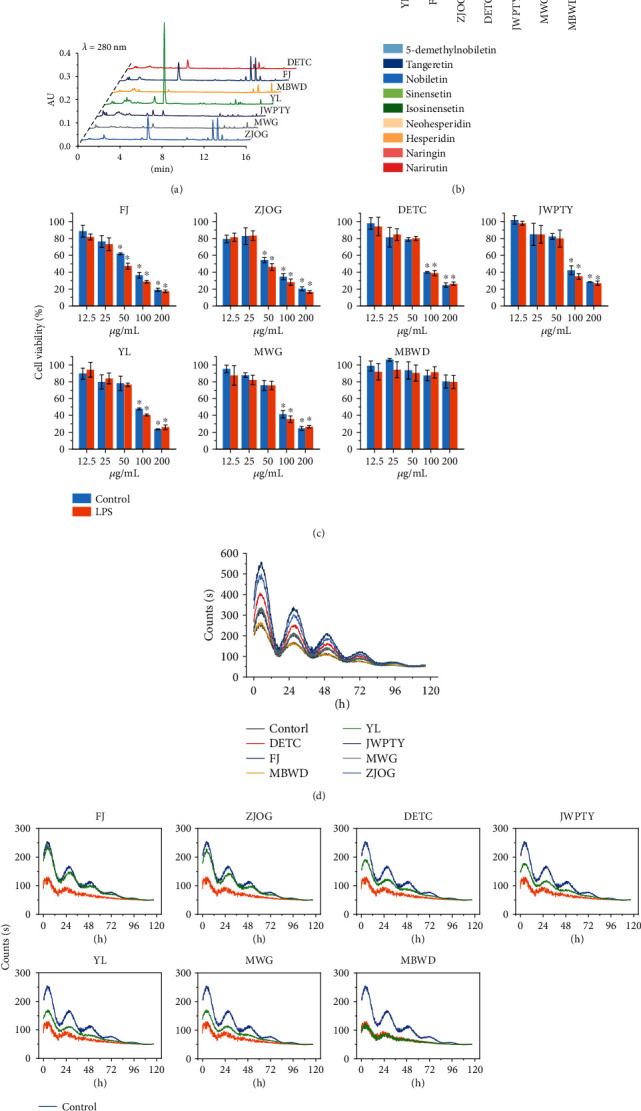
Regulation of citrus flavonoids on circadian rhythms of BV-2 cells: (a) HPLC chromatogram of flavonoid extracts from 7 varieties of citrus; (b) flavonoid contents in flavedo of 7 citrus varieties; (c) cell viabilities of BV-2 cells under different citrus flavonoid extracts and LPS treatments measured using a cell counting kit-8 assay; (d) effects of citrus flavonoid extracts on the expression of circadian rhythm gene *Per2* using luciferase bioluminescence reporters; (e) effects of citrus flavonoid extracts on LPS-disturbed expression of circadian gene *Per2*. FJ: Fuju; ZJOG: Zijinougan; YL: Youliang; DETC: Daetiancheng; MWG: Manwengan; JWPTY: Jiweiputaoyou; MBWD: Mabuwendan. Data is presented as the mean ± standard deviation (*n* = 3). ∗ indicates significant difference between flavonoid treatments compared to the DMSO solvent control (*p* < 0.05).

To compare the effects of different types of citrus fruit extracts on the expression of circadian rhythm genes, 5 types, 7 varieties of citrus fruits including Fuju (FJ, *C. reticulata*), Zijinougan (ZJOG, *C. reticulata* cv *Suavissima*), Youliang (YL, *Citrus unshiu*), Daetiancheng (DETC, *Citrus sinensis*), Manwengan (MWG, *Citrus hybrids*), Jiweiputaoyou (JWPTY, *Citrus paradisi*), and Mabuwendan (MBWD, *Citrus maxima*) were collected as extraction materials. UPLC was used to qualitatively and quantitatively analyze the flavonoids of citrus flavedo extracts. As shown in Figures 1(a) and 1(b), the flavonoid composition and content of different citrus varieties were different. Among the 7 citrus varieties, YL had the highest flavonoid content, while MBWD had the lowest flavonoid content. Quantitative analysis of 9 flavonoid compounds with high content found that citrus flavonoids were mainly composed of flavanones and PMFs, which was consistent with previous reports [9]. Neohesperidin was the main flavanone in ZJOG, MWG, and JWPTY; hesperidin was the main flavanone in FJ, DETC, and YL; and naringin was the main flavanone in MBWD. The PMF content of ZJOG (39.94 mg/g FW) and FJ (57.75 mg/g FW) was significantly higher than that of other citrus varieties, while MBWD contained almost no PMFs (0.36 mg/g FW), which was also consist with previous reports [18–21].

The *Per2* gene luciferase bioluminescence reporter system was used to detect the effect of citrus flavonoid extract on the expression of circadian rhythm genes in BV-2 cells. To prevent the influence of flavonoid cytotoxicity, we selected the citrus flavonoid extract concentration with a cell viability higher than 80% for subsequent experiments (FJ and ZJOG, 25 *μ*g/mL; DETC, JWPTY, YL, and MWG, 50 *μ*g/mL; and MBWD, 400 *μ*g/mL) (Figure 1(c)). Serum shock was used to induce circadian gene expression in BV-2 cells. The experimental results (Figure 1(d)) showed that except for MBWD, the other flavonoid extracts all induced the expression of *Per2* gene, and FJ and ZJOG had the strongest inducing effect. Citrus extract showed no effect on the phase of *Per2* gene expression. LPS was used to induce the disruption of *Per2* gene oscillatory expression. The results (Figure 1(e)) showed that LPS significantly inhibited the oscillating expression of *Per2* gene, while FJ and ZJOG treatments showed an inhibitory effect on LPS-induced disruption. DETC, JWPTY, YL, and MBWD also showed a similar effect, but not MBWD.

The induction of *Per2* oscillating expression by citrus extract was positively correlated with the PMF content. We speculated that PMFs were the main substances in citrus flavonoids that induced the oscillating expression of circadian clock genes. To verify this hypothesis, we conducted a follow-up experiment using 25 citrus flavonoid monomers.

### 3.2. Effects of Citrus Flavonoids on the Oscillating Expression of Circadian Genes Induced by Serum Shock

As shown in Table 1, 25 citrus flavonoids were collected to compare their regulatory effect on the expression of circadian rhythm genes. The structural differences among the flavonoids include the single or double carbon-carbon connection between the 2 and 3 positions of the C ring, the hydroxy or methoxy substituents number and position, and the substitution position of the glucoside, rutinoside, or neohesperidoside. To avoid the toxicity effects of flavonoids on BV-2 cells, we used different concentrations of flavonoids alone or together with LPS to incubate with the cells, then measured the cell viability using the cck-8 method. As shown in Figure S1, citrus flavonoids exhibited different proliferation inhibition abilities in BV-2 cells. Comparison of the cell viabilities to the flavonoid structures suggested that the differences in safe dose ranges were mainly related to the methoxy and glycoside substituents of the flavonoids. PMFs, including nobiletin, sinensetin, isosinensetin, tangeretin, 5-demethylnobiletin, and gardenin B, with 4 or more methoxy substituents, had a safe dose range below 20 *μ*M. Fifteen flavonoids containing glycosides exhibited a safe dose range below 320 *μ*M, including three flavonoids with glucoside (isovitexin, vitexin, and vicenin-2), six flavonoids with rutinoside (hesperidin, diosmin, didymin, narirutin, isorhoifolin, and eriocitrin), and six flavonoids with neohesperidoside (neohesperidin, neodiosmin, poncirin, naringin, rhoifolin, and neoeriocitrin). The other 4 flavonoids (hesperetin, apigenin, naringenin, and diosmetin) had a safe dose range below 160 *μ*M. The cell viabilities decreased compared to PMF treatment alone, when LPS was incubated with the cells after 12 h of PMF pretreatment. The safe dose range was below 10 *μ*M. For flavonoids with less than three methoxy substituents, the safe dose ranges were inconsistent between with and without LPS incubation. Therefore, the final concentrations of 10 *μ*M for nobiletin, sinensetin, isosinensetin, tangeretin, 5-demethylnobiletin, and gardenin B; 160 *μ*M for hesperetin, apigenin, naringenin, and diosmetin; and 320 *μ*M for isovitexin, vitexin, vicenin-2, hesperidin, neohesperidin, diosmin, neodiosmin, didymin, poncirin, narirutin, naringin, isorhoifolin, rhoifolin, eriocitrin, and neoeriocitrin were used for further experiments.

**Figure 2:**
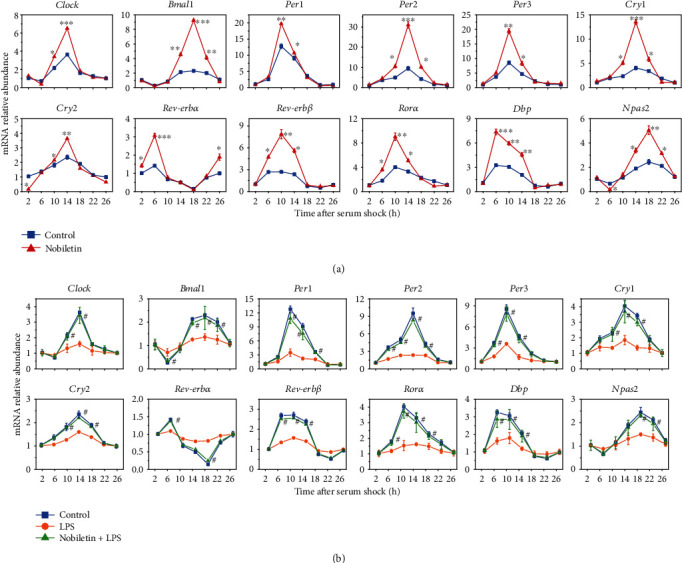
Effects of 10 *μ*M nobiletin pretreatment on the expression of circadian genes (a) and effects of 10 *μ*M nobiletin pretreatment on LPS-disturbed expression of circadian (b). *Clock*, *Bmal1*, *Per1*, *Per2*, *Per3*, *Cry1*, *Cry2*, *Rev-erbα*, *Rev-erbβ*, *Rorα*, *Dbp*, and *Npas2*, in BV-2 cells. Relative mRNA levels were measured using qRT-PCR. Data is presented as the mean ± standard deviation (*n* = 3). ^∗^*p* < 0.05, ^∗∗^*p* < 0.01, and ^∗∗∗^*p* < 0.001, compared to the DMSO blank control. ^#^*p* < 0.05 of treatments compared to the LPS-induced circadian clock disorder.

Then, the 25 flavonoids, at safe dose ranges, were used to pretreat BV-2 cells for 24 h, and serum shock was used to induce the expression of circadian genes, including *Clock*, *Bmal1*, *Per1*, *Per2*, *Per3*, *Cry1*, *Cry2*, *Rev-erbα*, *Rev-erbβ*, *Rorα*, *Dbp*, and *Npas2*. As shown in Figure 2(a) and Figure S2, eight out of 25 flavonoids exhibited circadian gene regulatory abilities, all of which share the structural characteristic of containing a methoxy substituent. For treatments using flavonoids containing glycosides or containing no methoxy groups, circadian genes did not show significant differences between treatment and control groups. The main function of flavonoids in gene regulation was to promote the expression of circadian rhythm genes in the crest of the time point. The more the methoxy substitutes the PMFs had, the higher the expression of the genes. Nobiletin, sinensetin, isosinensetin, tangeretin, gardenin B, 5-demethylnobiletin, diosmetin, and hesperetin inhibited *Cry2* gene expression in the troughs (*p* < 0.05). Also, *Npas2* gene expression in the troughs was inhibited by nobiletin, sinensetin, isosinensetin, and gardenin B (*p* < 0.05).

### 3.3. Inhibition of LPS-Induced Circadian Clock Disorder Mediated by Citrus Flavonoids in BV-2 Cells

Previous studies have reported that LPS could induce disorders in the expression of circadian rhythm genes [8]. To investigate the mitigation effect of flavonoids on LPS-induced circadian rhythm disturbance, BV-2 cells were pretreated with flavonoids for 12 h before LPS was added to the medium. Serum shock was used to induce expression of circadian genes after LPS was incubated with cells for another 12 h. Results (Figure 2(b) and Figure S3) showed that LPS inhibited the oscillating expression of circadian-related genes by decreasing gene expression in crests and increasing gene expression in troughs. Among the 25 flavonoids tested, only 6 PMFs exhibited inhibitory effects on LPS-induced circadian disorder (*p* < 0.05). Other flavonoids with less than four methoxy groups did not show significant regulatory effects. In addition, the number of methoxy groups was particularly important. PMFs with a higher methoxy content exhibited stronger effect, in which nobiletin showed the best regulatory ability. At the 5th position, the substitution of methoxy by hydroxy decreased the activity of PMFs.

### 3.4. Inhibition of LPS-Induced NO-Release Mediated by Citrus Flavonoids

**Figure 3:**
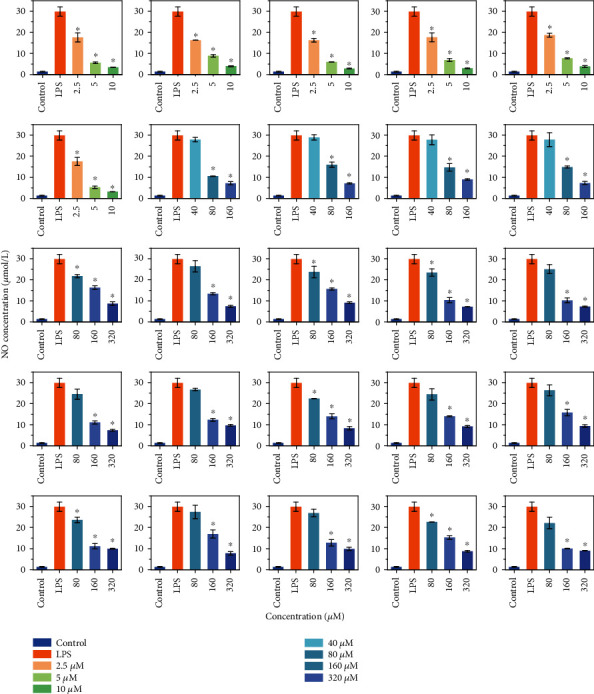
Inhibition of LPS-induced NO release by treatment with different concentrations of flavonoids. NO concentration was determined using a NO assay kit. Data is presented as the mean ± standard deviation (*n* = 3). ∗ indicates significant difference between flavonoid treatments compared to the LPS treatment (*p* < 0.05).

LPS is a reagent that could cause cellular inflammatory reactions, while flavonoids are reported to exhibit anti-inflammatory activities [11, 22–24]. To investigate whether regulation of the circadian rhythm is associated with inhibition of inflammation, we determined the concentration of NO release from BV-2 cells after LPS induction. As shown in Figure 3, within their safe dose ranges, all 25 citrus flavonoids exhibited inhibitory capabilities on the NO release induced by LPS (*p* < 0.05), although the effective concentrations varied. PMFs were effective in test concentration ranges from 2.5 to 10 *μ*M and exhibited a gradient effect, while for diosmetin, hesperetin, apigenin, and naringenin, the effective concentration ranges were 80 to 100 *μ*M. Glycosylated flavonoids were effective from 80 or 160 *μ*M to 320 *μ*M. To further investigate the mechanism by which flavonoids inhibit inflammation, the most effective flavonoid treatment concentration was selected for mechanism exploration experiments.

**Figure 4:**
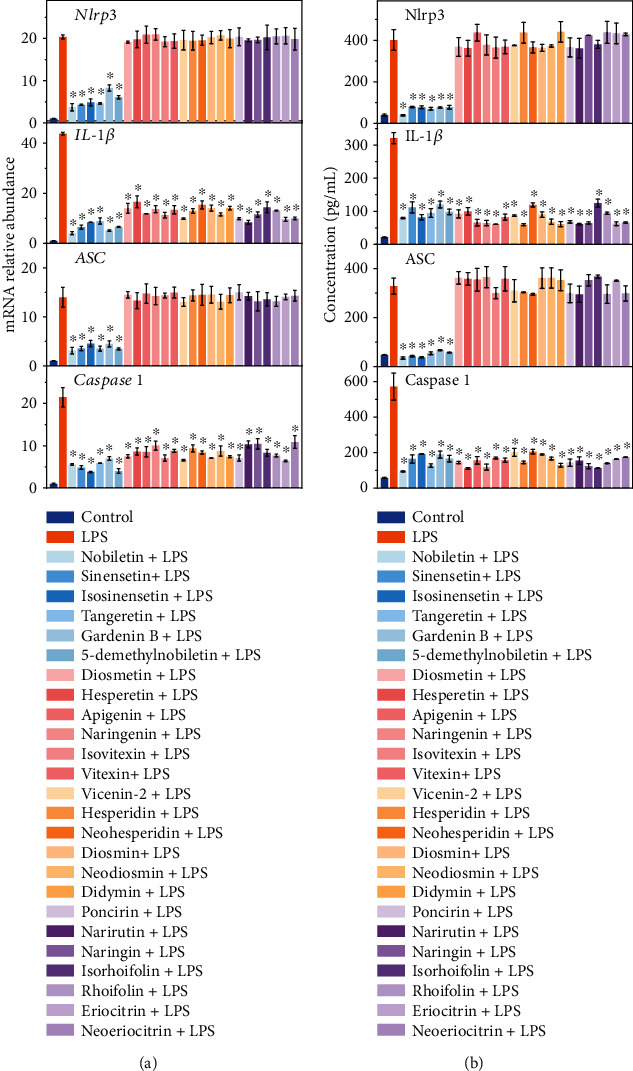
Regulation of Nlrp3 expression by citrus flavonoids in BV-2 cells: (a) regulation of *Nlrp3* inflammasome-related genes, *Nlrp3*, *IL-1β*, *ASC*, and *Caspase 1*, by citrus flavonoids (indicated in the color-coded legend to the right of panel), as measured by qRT-PCR; (b) ELISA detection of Nlrp3 inflammasome-related proteins regulated by flavonoids (indicated by the color-coded legend to the right of panel). The pretreatment concentration was 10 *μ*M for nobiletin, sinensetin, isosinensetin, tangeretin, 5-demethylnobiletin, and gardenin B; 160 *μ*M for diosmetin, hesperetin, apigenin, and naringenin; and 320 *μ*M for isovitexin, vitexin, vicenin-2, hesperidin, diosmin, didymin, narirutin, isorhoifolin, eriocitrin, neohesperidin, neodiosmin, poncirin, naringin, rhoifolin, and neoeriocitrin. Data is presented as the mean ± standard deviation (*n* = 3). ∗ indicates significant difference between flavonoid treatments compared to the LPS treatment (*p* < 0.05).

The Nlrp3 inflammasome was found to be related to the circadian rhythm according to previous reports [8, 25]. To explore whether the flavonoid-mediated regulation of the circadian rhythm disturbance was related to the transcription and translation of the Nlrp3 inflammasome, Nlrp3 inflammasome RNA and protein expression was detected. Results (Figures 4(a) and 4(b)) showed that LPS increased the expression of Nlrp3-related genes and proteins, while flavonoids with different structures exhibited different regulatory effects. PMFs inhibited *Nlrp3*, *IL-1β*, *ASC*, and *Capase1* gene and protein expression (*p* < 0.05), while the other 19 flavonoids selectively inhibited *IL-1β* and *Capase1* (*p* < 0.05) and showed no significant difference in the regulation of *Nlrp3* and *ASC*. These results indicate that the regulation of PMFs on LPS-induced circadian disturbance might be related to the *Nlrp3* and *ASC* genes in the Nlrp3 inflammasome.

### 3.5. Regulation of Nobiletin on the LPS-Induced Circadian Disorder in Mouse Liver

**Figure 5:**
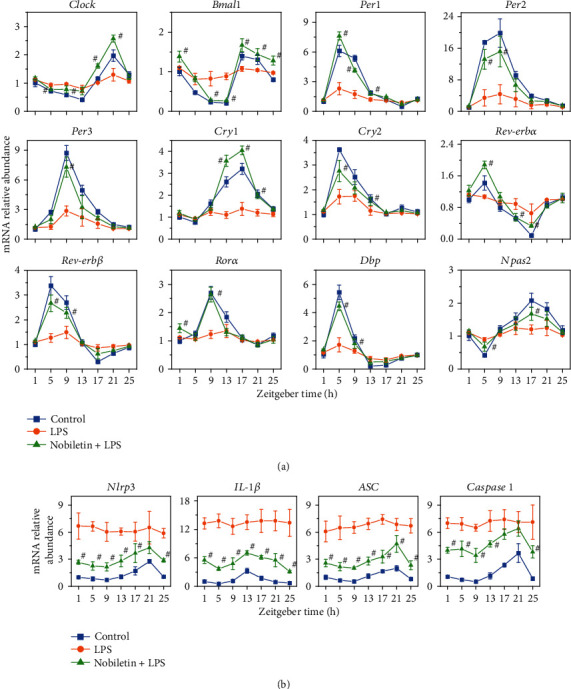
Regulation effects of nobiletin on the mouse liver circadian gene expressions (a) and Nlrp3-related gene expressions (b). Relative mRNA levels were measured using qRT-PCR. Data is presented as the mean ± standard deviation (*n* = 5). ^#^*p* < 0.05 of treatments compared to the LPS treatment.

To test whether PMFs could inhibit LPS-induced liver circadian clock disturbance in mice, we selected nobiletin as a representative substance for *in vivo* experiments. As shown in Figure 5(a), intraperitoneal injection of LPS induced the dysregulation of circadian rhythm gene oscillation expression in mouse liver, while feeding nobiletin at a dose of 200 mg/kg body weight effectively inhibited this dysregulation (*p* < 0.05).

Nlrp3 inflammasome-related gene expression at different time points were detected and found that LPS treatment upregulated the expression of gene expression including *Nlrp3*, *IL-1β*, *ASC*, and *Caspase-1* but at the same time attenuated the oscillatory expression of these genes (Figure 5(b)). Nobiletin gavage treatment inhibited the expression of genes such as *Nlrp3* and at the same time significantly inhibited the oscillation expression disorder induced by LPS (*p* < 0.05).

The above results indicated that nobiletin could regulate the oscillating expression of circadian clock genes *in vitro* and *in vivo* and could inhibit LPS-induced circadian rhythm disorders. At the same time, we speculated that the inhibitory effect of PMFs on LPS may be related to the Nlrp3 inflammasome. To further verify this hypothesis, we performed a follow-up test using INF39, an inhibitor of Nlrp3.

### 3.6. Preliminary Verification Using Nlrp3 Inhibitor, INF39

**Figure 6:**
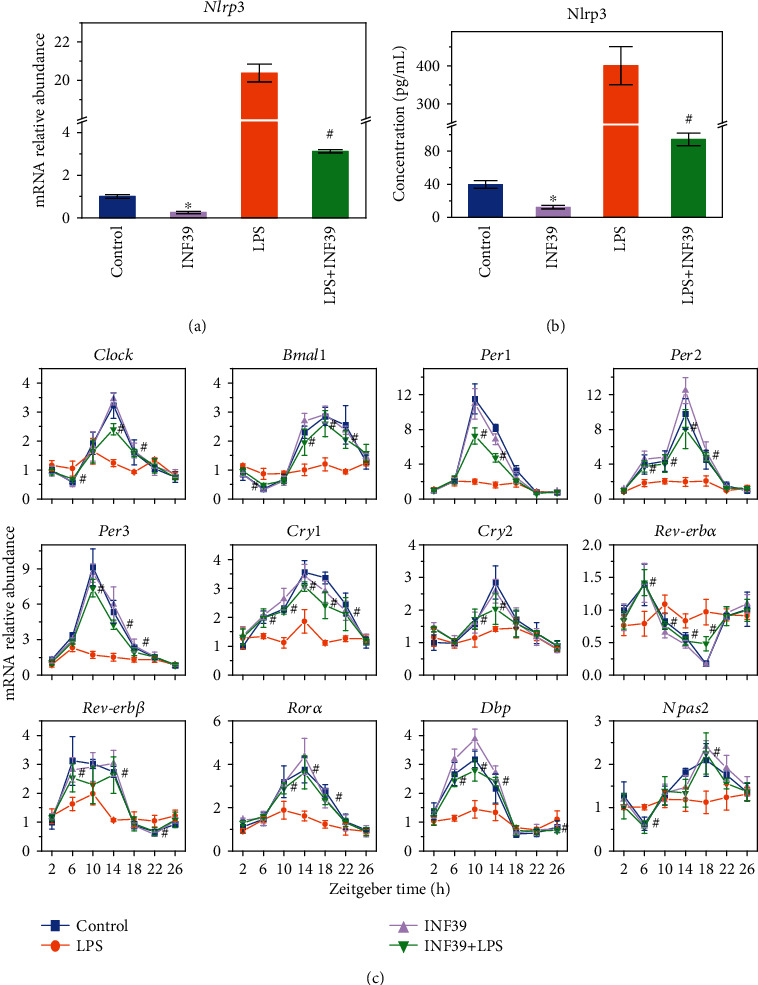
Preliminary verification of the mechanism by nobiletin-regulated circadian gene expression in BV-2 cells using the Nlrp3 inhibitor, INF39: (a) regulation of *Nlrp3* gene expression by LPS and INF39; (b) ELISA detection of Nlrp3 protein expression regulated by LPS and INF39; (c) effects of INF39-regulated circadian gene expression, as measured by qRT-PCR. Data is presented as the mean ± standard deviation (*n* = 3). ∗ indicates significant difference between INF treatments compared to control. # indicates significant difference between LPS treatments compared to the LPS and INF39 combination treatment (*p* < 0.05).

To verify whether the regulation of PMFs on LPS-induced circadian rhythm disorder was associated with the *Nlrp3* inflammasome, we used Nlrp3 inhibitor, INF39, to coincubate with LPS, then measured the expression of genes related to the circadian rhythm. As shown in Figures 6(a) and 6(b), INF39 significantly inhibited the expression of Nlrp3 gene and protein (*p* < 0.05). Circadian rhythm gene detection found that, in the absence of LPS treatment, INF39 itself did not significantly affect the circadian genes (Figure 6(c)) (*p* < 0.05). However, INF39 inhibited the disturbance of LPS on the circadian rhythm, which showed a similar effect of PMFs. These results preliminarily verified that LPS-induced circadian disorders were related to Nlrp3 inflammasome, and PMFs could restore LPS-induced circadian rhythm gene oscillation disruption by inhibiting Nlrp3.

## 4. Discussion

The orderly progression of the biological clock is one of the necessary conditions for the normal operation of living organisms. In mammals, the suprachiasmatic nucleus (SCN) is the central structure of the circadian rhythm regulatory system (Reppert et al., 2002). The circadian clock plays the role of a medium to regulate the internal milieu to adapt to the external environment in a proper time sequence, such as learning and sleeping [26].

*In vitro* screening facilitates the study of natural products and the exploration of their mechanism. The luciferase bioluminescence reporter system is a classic biological clock screening method [15]. Simulation of circadian rhythm oscillating operation induced by treatment, such as serum shock, provides a means for high-throughput screening of predrugs and nutrient factors [16]. We screened the circadian clock regulation ability through *Per2* gene expression and found that the content of PMFs in citrus flavonoid extracts was positively correlated with circadian gene expression oscillation.

Twenty-five citrus flavonoids were selected for further research. The specificity of their structural characteristics includes the type and number of substituents and the connection state between the 2nd and 3rd carbons. In this experiment, we compared the structure-activity relationship of citrus flavonoids in three aspects, including circadian clock enhancement, inhibition of NO release, and regulation of LPS-induced circadian disturbances.

The roles of methoxy and glycoside substituents in the flavonoid structure were relatively noticeable. In the circadian gene expression enhancing assays, the structure-activity relationship of flavonoids showed the positive effect of methoxy groups and the adverse effect of glycosides. The results indicated that the presence of methoxy groups was indispensable for enhancing the oscillating expression of circadian rhythms. However, if the structure contained glycosides, the regulatory ability of the flavonoids was greatly diminished. These results were consistent with previous reports [23], where both nobiletin and tangeretin exhibited a regulatory effect on the circadian rhythm, while glucoside-containing flavonoids, naringenin and naringin, did not exhibit circadian regulation abilities.

The LPS treatment also caused circadian rhythm disturbance of BV-2 cells, mainly exhibited as inhibition of oscillating expression of circadian-related genes. Among the 25 tested flavonoids, only the PMFs showed a rescuing effect on circadian clock gene expression oscillation, suggesting that not only the existence of the methoxy group but also the number of methoxy groups is important for the regulatory ability of PMFs. Nobiletin was tested *in vivo* as a representative substance of PMFs, and it was found that nobiletin had an *in vivo* inhibitory effect on LPS-induced biological rhythm disorders.

It has been reported that exogenous or endogenous inflammatory factors could induce disorders of the circadian clock, such as inhibition of the oscillating expression of cell clock genes and proteins [23]. However, does inhibition of inflammation necessarily promote the normal expression of circadian rhythm genes? This study partially answers this question. Nineteen out of the 25 tested flavonoids did not affect LPS-induced circadian rhythm disturbance, suggesting that the regulation of the circadian rhythm seems to be conditional. For the flavonoids we tested, the characteristic structure that was critical for regulatory capability was the number of methoxy groups present in the PMFs. Previous reports have shown that the *Nlrp3* inflammasome participates in the interaction between the circadian clock and inflammation [8], so we examined the expression of genes and proteins associated with the *Nlrp3* inflammasome, including *Nlrp3*, *ASC*, *IL-1β*, and *Caspase1*. Results showed that the effects of flavonoids on the expression of *Nlrp3* and *ASC* genes were different. Only PMFs showed inhibitory effects, while other flavonoids, which did not regulate LPS-induced circadian clock disorder, did not inhibit *Nlrp3* and *ASC* gene expression. These results suggest that different mechanisms may be responsible for the same NO-releasing inhibition phenotype. Moreover, the difference between the mechanism of PMFs and non-PMFs is associated with the *Nlrp3* inflammasome. We preliminarily verified this conjecture using the *Nlrp3* inhibitor INF39.

For our hypothesis, we concluded that PMFs, flavonoids with a special structure, exhibited inhibition of inflammation-related circadian clock disorders and that the regulatory capabilities were associated with inhibition of the *Nlrp3* gene. In a few previous reports, some non-PMFs have been found to regulate Nlrp3 inflammation through Nlrp3 inflammasome inhibition, such as in the case of apigenin-ameliorated chronic mild stress-induced depressive behavior [27]. However, the experimental systems and treatment methods of these reports differ from our research, and the reported studies were not circadian-related. We will continue paying attention to reveal this contradiction.

## 5. Conclusion

We compared the circadian rhythm regulation abilities of 7 citrus varieties and 25 citrus flavonoids. Flavonoids were structurally specific for the regulation of the circadian rhythm. Methoxy groups promote regulating abilities, while glycosides inhibit regulatory abilities. LPS inhibited the oscillating expression of circadian rhythm genes. PMFs attenuated LPS-induced interference by inhibiting the *Nlrp3* gene. This study shed light on natural product research and the potential application of flavonoids in circadian rhythm regulation.

## Figures and Tables

**Table 1:** The citrus flavonoids used in this study and their substituents. Numbered columns indicate the position number of the substituents.

Flavonoids	Chemical name	2 and 3	5	6	7	8	3′	4′
Nobiletin	5,6,7,8,3′,4′-Hexamethoxyflavone	Double	OCH_3_	OCH_3_	OCH_3_	OCH_3_	OCH_3_	OCH_3_
Sinensetin	5,6,7,3′,4′-Pentamethoxyflavone	Double	OCH_3_	OCH_3_	OCH_3_	H	OCH_3_	OCH_3_
Isosinensetin	5,7,8,3′,4′-Pentamethoxyflavone	Double	OCH_3_	H	OCH_3_	OCH_3_	OCH_3_	OCH_3_
Tangeretin	5,6,7,8,4′-Pentamethoxyflavone	Double	OCH_3_	OCH_3_	OCH_3_	OCH_3_	H	OCH_3_
Gardenin B	5-Hydroxy-6,7,8,4′-tetramethoxyflavone	Double	OH	OCH_3_	OCH_3_	OCH_3_	H	OCH_3_
5-Demethylnobiletin	5-Hydroxy-6,7,8,3′,4′-pentamethoxyflavone	Double	OH	OCH_3_	OCH_3_	OCH_3_	OCH_3_	OCH_3_
Hesperetin	5,7,3′-Trihydroxy-4′-methoxyflavanone	Single	OH	H	OH	H	OH	OCH_3_
Apigenin	5,7,4′-Trihydroxyflavone	Double	OH	H	OH	H	H	OH
Naringenin	5,7,4′-Trihydroxyflavanone	Single	OH	H	OH	H	H	OH
Diosmetin	5,7,3′-Trihydroxy-4′-methoxyflavone	Double	OH	H	OH	H	OH	OCH_3_
Isovitexin	5,7,4′-Trihydroxyflavone-6-*C*-glucoside	Double	OH	Glu^1^	OH	H	H	OH
Vitexin	5,7,4′-Trihydroxyflavone-8-*C*-glucoside	Double	OH	H	OH	Glu	H	OH
Vicenin-2	5,7,4′-Trihydroxyflavone-6,8-di-*C*-glucoside	Double	OH	Glu	OH	Glu	H	OH
Hesperidin	5,7,3′-Trihydroxy-4′-methoxyflavanone-7-*O*-rutinoside	Single	OH	H	*O*-Rut^2^	H	OH	OCH_3_
Neohesperidin	5,7,3′-Trihydroxy-4′-methoxyflavanone-7-*O*-neohesperidoside	Single	OH	H	*O*-Nhp^3^	H	OH	OCH_3_
Diosmin	5,7,3′-Trihydroxy-4′-methoxyflavone-7-*O*-rutinoside	Double	OH	H	*O*-Rut	H	OH	OCH_3_
Neodiosmin	5,7,3′-Trihydroxy-4′-methoxyflavone-7-*O*-neohesperidoside	Double	OH	H	*O*-Nhp	H	OH	OCH_3_
Didymin	5,7-Dihydroxy-4′-methoxyflavanone-7-*O*-rutinoside	Single	OH	H	*O*-Rut	H	H	OCH_3_
Poncirin	5,7-Dihydroxy-4′-methoxyflavanone-7-*O*-neohesperidoside	Single	OH	H	*O*-Nhp	H	H	OCH_3_
Narirutin	5,7,4′-Trihydroxyflavanone-7-*O*-rutinoside	Single	OH	H	*O*-Rut	H	H	OH
Naringin	5,7,4′-Trihydroxyflavanone-7-*O*-neohesperidoside	Single	OH	H	*O*-Nhp	H	H	OH
Isorhoifolin	5,7,4′-Trimethoxyflavone-7-*O*-rutinoside	Double	OH	H	*O*-Rut	H	H	OH
Rhoifolin	5,7,4′-Trihydroxyflavone-7-O-neohesperidoside	Double	OH	H	*O*-Nhp	H	H	OH
Eriocitrin	5,7,3′,4′-Tetrahydroxyflavanone-7-O-rutinoside	Single	OH	H	*O*-Rut	H	OH	OH
Neoeriocitrin	5,7,3′,4′-Tetrahydroxyflavanone-7-O-neohesperidoside	Single	OH	H	*O*-Nhp	H	OH	OH

^1^Glu: glucoside; ^2^Rut: rutinoside; ^3^Nhp: neohesperidoside.

## Data Availability

We have already included the available data in our manuscript.

## Supplementary Materials

Supplementary Materials Table S1: the sequences of the primers used for quantitative real-time polymerase chain reaction (qRT-PCR) detection. Figure S1: cell viabilities of BV-2 cells under different flavonoid and LPS treatments measured using a cell counting kit-8. Data is presented as the mean ± standard deviation (*n* = 3). ^∗^*p* < 0.05, compared to the DMSO blank control. Figure S2: effects of flavonoid pretreatment on the expression of circadian genes, *Clock*, *Bmal1*, *Per1*, *Per2*, *Per3*, *Cry1*, *Cry2*, *Rev-erbα*, *Rev-erbβ*, *Rorα*, *Dbp,* and *Npas2*, in BV-2 cells. Relative mRNA levels were measured using qRT-PCR. The pretreatment concentration was 10 *μ*M for sinensetin, isosinensetin, tangeretin, 5-demethylnobiletin, and gardenin B; 160 *μ*M for diosmetin, hesperetin, apigenin, and naringenin and 320 *μ*M for isovitexin, vitexin, vicenin-2, hesperidin, diosmin, didymin, narirutin, isorhoifolin, and eriocitrin, neohesperidin, neodiosmin, poncirin, naringin, rhoifolin, and neoeriocitrin. Data is presented as the mean ± standard deviation (*n* = 3). ^∗^*p* < 0.05, ^∗∗^*p* < 0.01, and ^∗∗∗^*p* < 0.001, compared to the DMSO blank control. Figure S3: effects of flavonoid nobiletin pretreatment on LPS-disturbed expression of circadian genes, *Clock*, *Bmal1*, *Per1*, *Per2*, *Per3*, *Cry1*, *Cry2*, *Rev-erbα*, *Rev-erbβ*, *Rorα*, *Dbp*, and *Npas2*, in BV-2 cells. Relative mRNA levels were determined using qRT-PCR. The pretreatment concentration was 10 *μ*M for sinensetin, isosinensetin, tangeretin, 5-demethylnobiletin, and gardenin B; 160 *μ*M for diosmetin, hesperetin, apigenin, and naringenin, and 320 *μ*M for isovitexin, vitexin, vicenin-2, hesperidin, diosmin, didymin, narirutin, isorhoifolin, eriocitrin, neohesperidin, neodiosmin, poncirin, naringin, rhoifolin, and neoeriocitrin. Data is presented as the mean ± standard deviation (*n* = 3). ^#^*p* < 0.05 of treatments compared to the LPS-induced circadian clock disorder.
