# Supplementary material for: Polymethoxyflavones in Citrus Regulate Lipopolysaccharide-Induced Oscillating Decay of Circadian Rhythm Genes by Inhibiting Nlrp3 Expression
Source: Oxid Med Cell Longev. 2021 Sep 14;2021:8419415. doi: 10.1155/2021/8419415 (PMC8457985; doi:10.1155/2021/8419415)
Supplement: Supplementary Materials — Table S1: the sequences of the primers used for quantitative real-time polymerase chain reaction (qRT-PCR) detection. Figure S1: cell viabilities of BV-2 cells under different flavonoid and LPS treatments measured using a cell counting kit-8. Data is presented as the mean ± standard deviation (n = 3). ∗p < 0.05, compared to the DMSO blank control. Figure S2: effects of flavonoid pretreatment on the expression of circadian genes, Clock, Bmal1, Per1, Per2, Per3, Cry1, Cry2, Rev-erbα, Rev-erbβ, Rorα, Dbp, and Npas2, in BV-2 cells. Relative mRNA levels were measured using qRT-PCR. The pretreatment concentration was 10 μM for sinensetin, isosinensetin, tangeretin, 5-demethylnobiletin, and gardenin B; 160 μM for diosmetin, hesperetin, apigenin, and naringenin and 320 μM for isovitexin, vitexin, vicenin-2, hesperidin, diosmin, didymin, narirutin, isorhoifolin, and eriocitrin, neohesperidin, neodiosmin, poncirin, naringin, rhoifolin, and neoeriocitrin. Data is presented as the mean ± standard deviation (n = 3). ∗p < 0.05, ∗∗p < 0.01, and ∗∗∗p < 0.001, compared to the DMSO blank control. Figure S3: effects of flavonoid nobiletin pretreatment on LPS-disturbed expression of circadian genes, Clock, Bmal1, Per1, Per2, Per3, Cry1, Cry2, Rev-erbα, Rev-erbβ, Rorα, Dbp, and Npas2, in BV-2 cells. Relative mRNA levels were determined using qRT-PCR. The pretreatment concentration was 10 μM for sinensetin, isosinensetin, tangeretin, 5-demethylnobiletin, and gardenin B; 160 μM for diosmetin, hesperetin, apigenin, and naringenin, and 320 μM for isovitexin, vitexin, vicenin-2, hesperidin, diosmin, didymin, narirutin, isorhoifolin, eriocitrin, neohesperidin, neodiosmin, poncirin, naringin, rhoifolin, and neoeriocitrin. Data is presented as the mean ± standard deviation (n = 3). #p < 0.05 of treatments compared to the LPS-induced circadian clock disorder. [file 8419415.f1.docx]

**Table S1**

**Table S1.** The sequences of the primers used for quantitative Real-Time polymerase chain reaction (qRT-PCR) detection.

| **Genes** | **Sequences** |
| --- | --- |
| *Gapdh* | F: TCA ACG GCA CAG TCA AGG  R: ACT CCA CGA CAT ACT CAG C |
| *Clock* | F: TCT GGA TTC GCT GGC TAA TGG  R: GAC CTC CGC TGT GTC ATC TT |
| *Bmal1* | F: CTC CAG GAG GCA AGA AGA TTC  R: ATA GTC CAG TGG AAG GAA TG |
| *Per1* | F: CCC AGC TTT ACC TGC AGA AG  R: ATG GTC GAA AGG AAG CCT CT |
| *Per2* | F: CCA CAC TTG CCT CCG AAA TA  R: ACT GCC TCT GGA CTG GAA GA |
| *Per3* | F: GGT CGA CAT AAA GTC CGA ACG A  R: TCG TTA CTG GCT GCC TTT TTT ATT |
| *Cry1* | F: CCC AGG CTT TTC AAG GAA TGG AAC  R: GCA GGG AGT TTG CAT TCA TTC GAG |
| *Cry2* | F: TGT CCC TTC CTG TGT GGA AGA  R: GCT CCC AGC TTG GCT TGA |
| *Rev-erbα* | F: TTT TTC GCC GGA GCA TCC AA  R: ATC TCG GCA AGC ATC CGT TG |
| *Rev-erbβ* | F: GGA GTT CAT GCT TGT GAA GGC TGT  R: CAG ACA CTT CTT AAA GCG GCA CTG |
| *Rorα* | F: GAG ACC CCG CTG ACC CA  R: TGA CTG AGA TAC CTC GGC TG |
| *Dbp* | F: ACA TCT AGG GAC ACA CCC AGT C  R: AAG TCT CAT GGC CTG GAA TG |
| *Npas2* | F: CAG GAC TGG AAG CCA TCA TT  R: CTG ATG TTG GAG GCA TTA GAT GGC |
| *Nlrp3* | F: ATT ACC CGC CCG AGA AAG G  R: TCG CAG CAA AGA TCC ACA CAG |
| *IL-1β* | F: AGT AAG TTC CTC TCT GCA AGA GAC T  R: CAC TAG GTT TGC CGA GTA GAT CTC |
| *ASC* | F: CTG GAG TCG TAT GGC TTG GAG  R: CAA AGT GTC CTG TTC TGG CTG TA |
| *Casp1* | F: ACA AGG CAC GGG ACC TAT G  R: TCC CAG TCA GTC CTG GAA ATG |

**Figure S1**


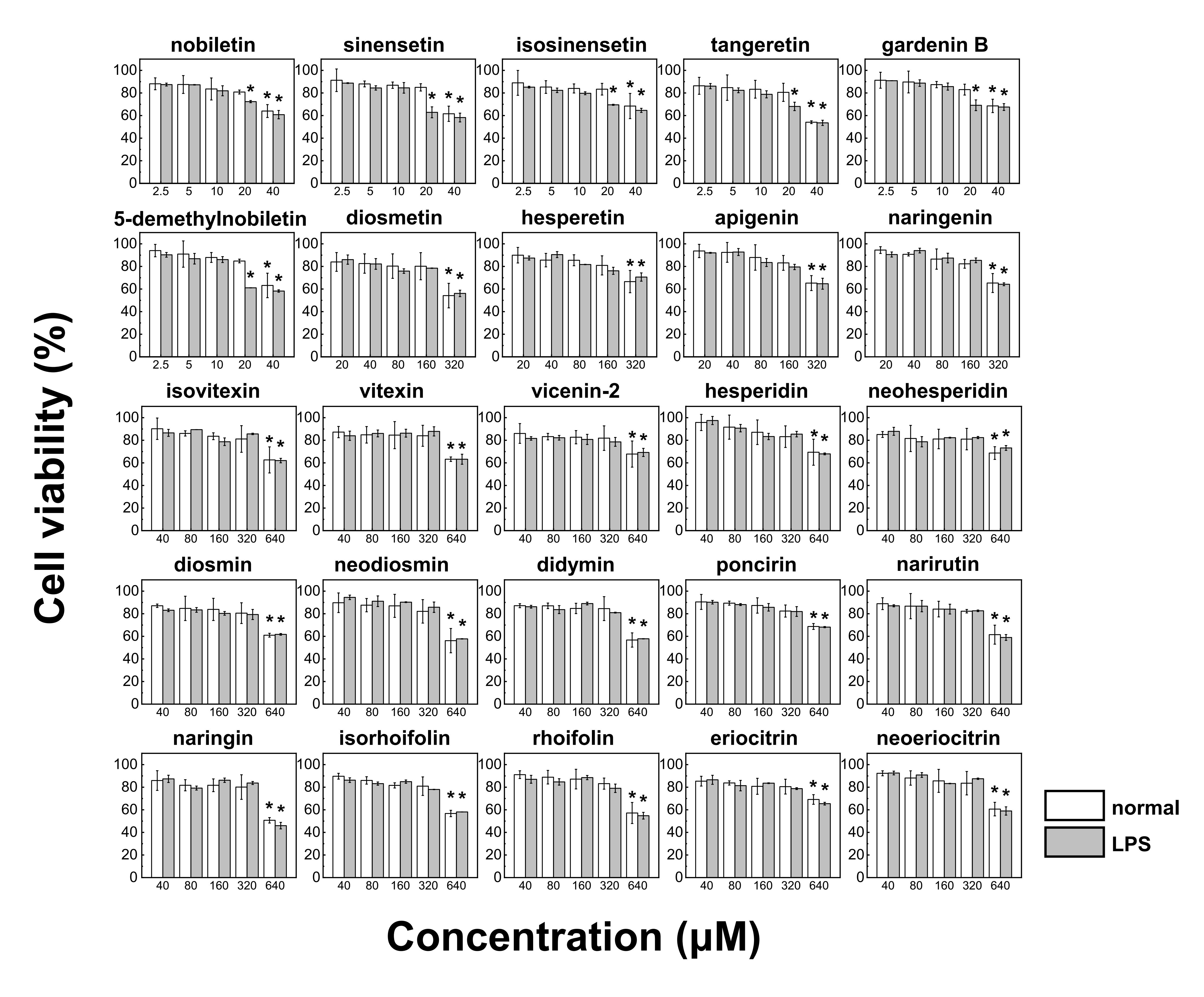


**Figure S1.** Cell viabilities of BV-2 cells under different flavonoid and LPS treatments measured using a cell count kit-8. Data is presented as the mean ± standard deviation (n=3). * *p* < 0.05, compared to the DMSO blank control.

**Figure S2**


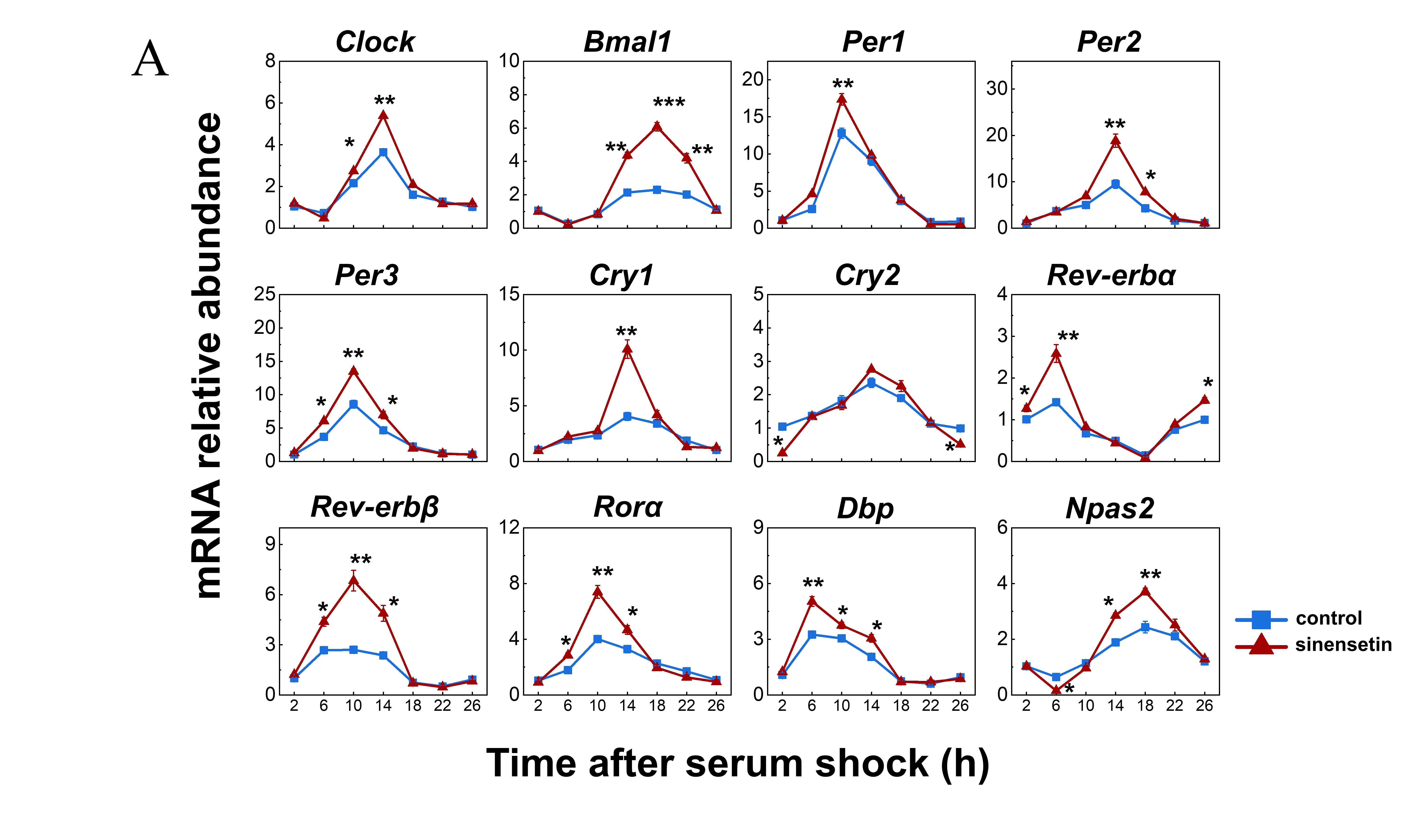

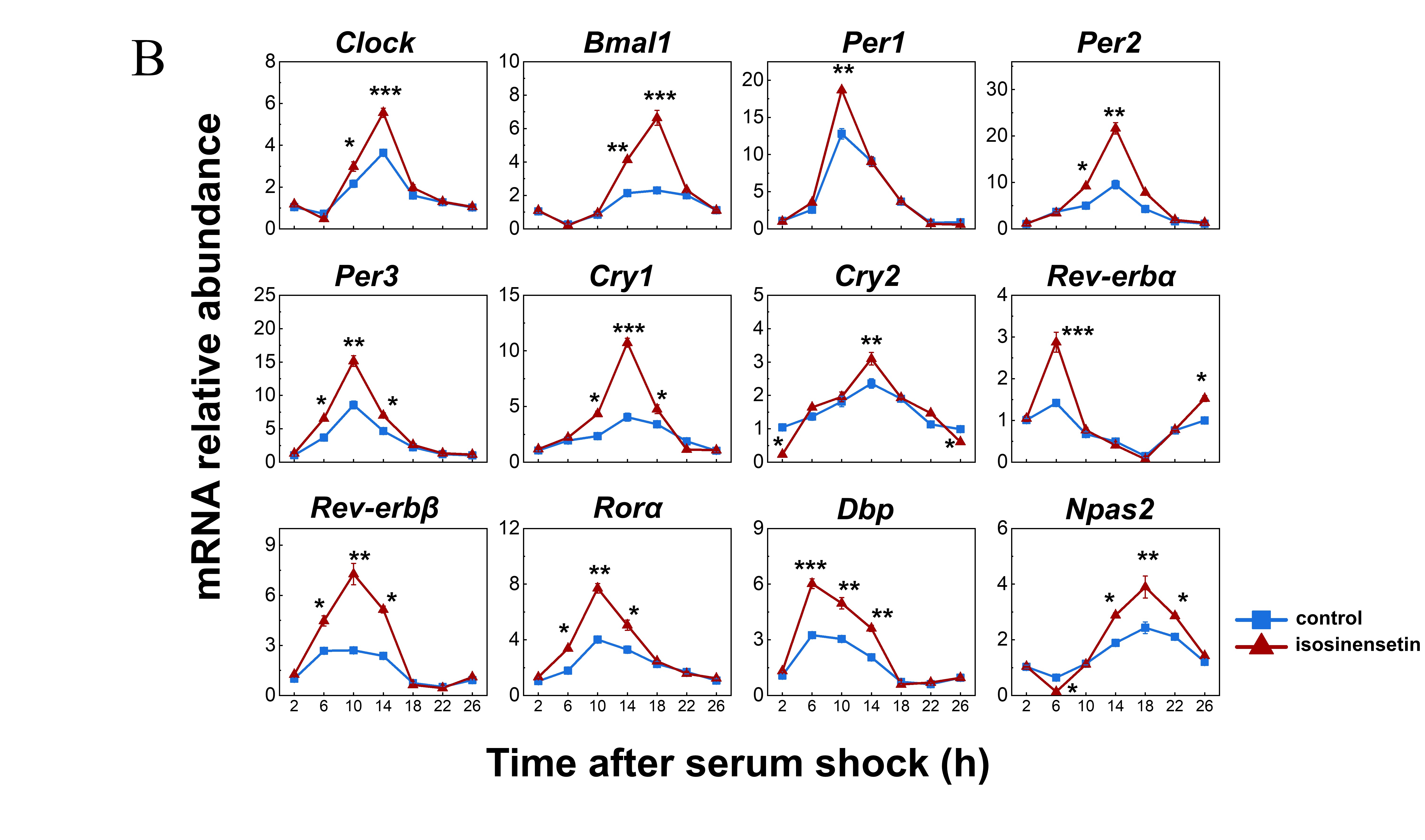

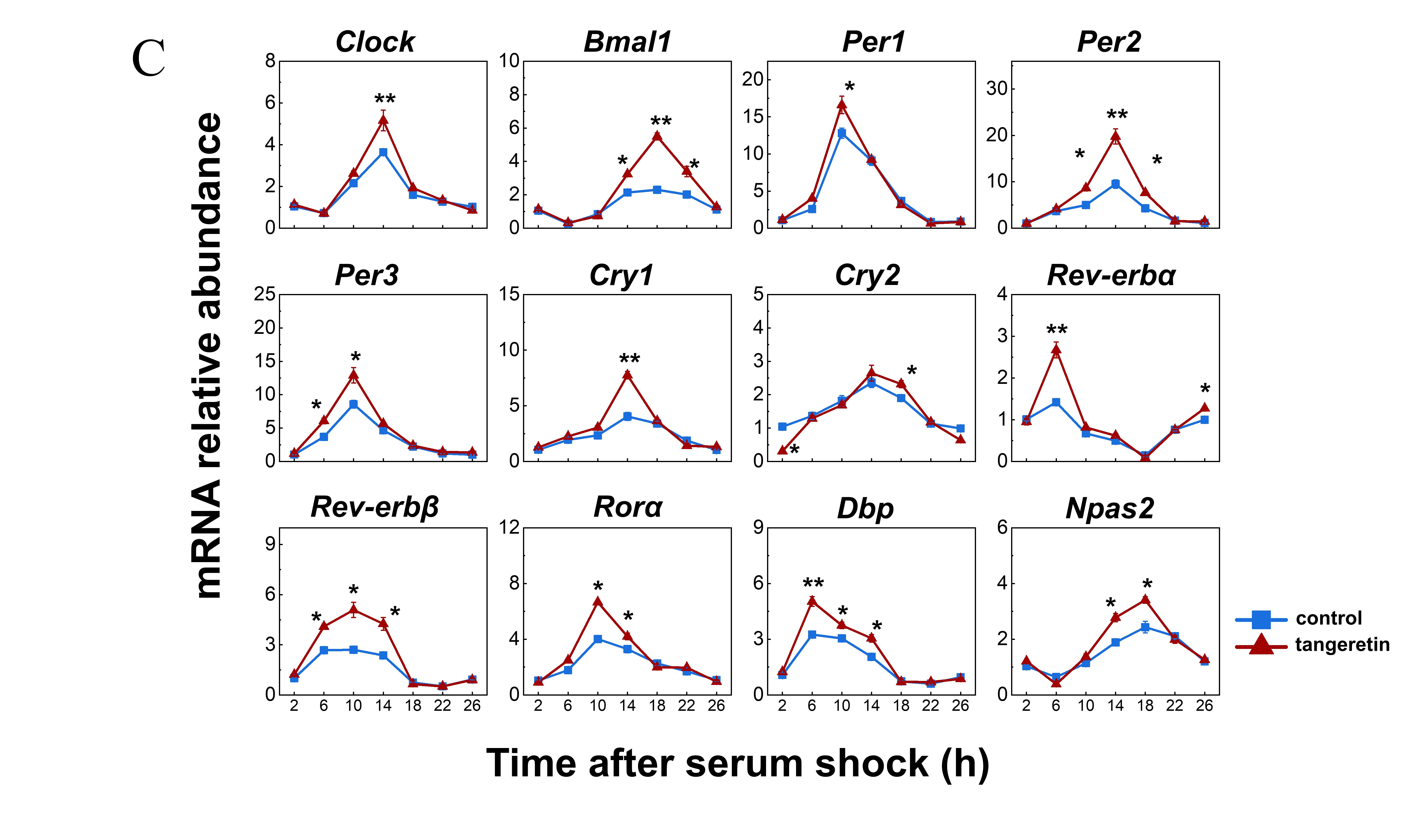

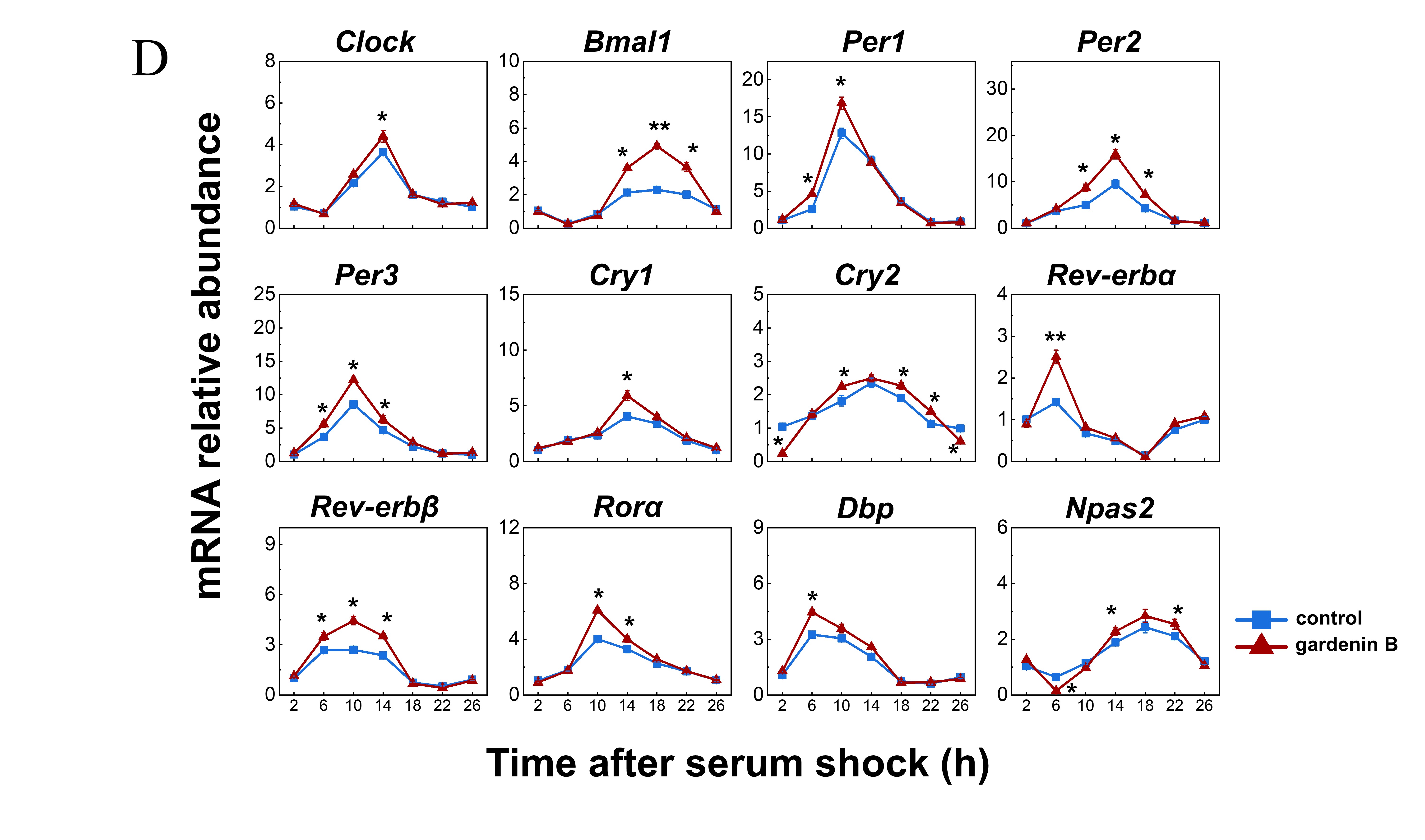

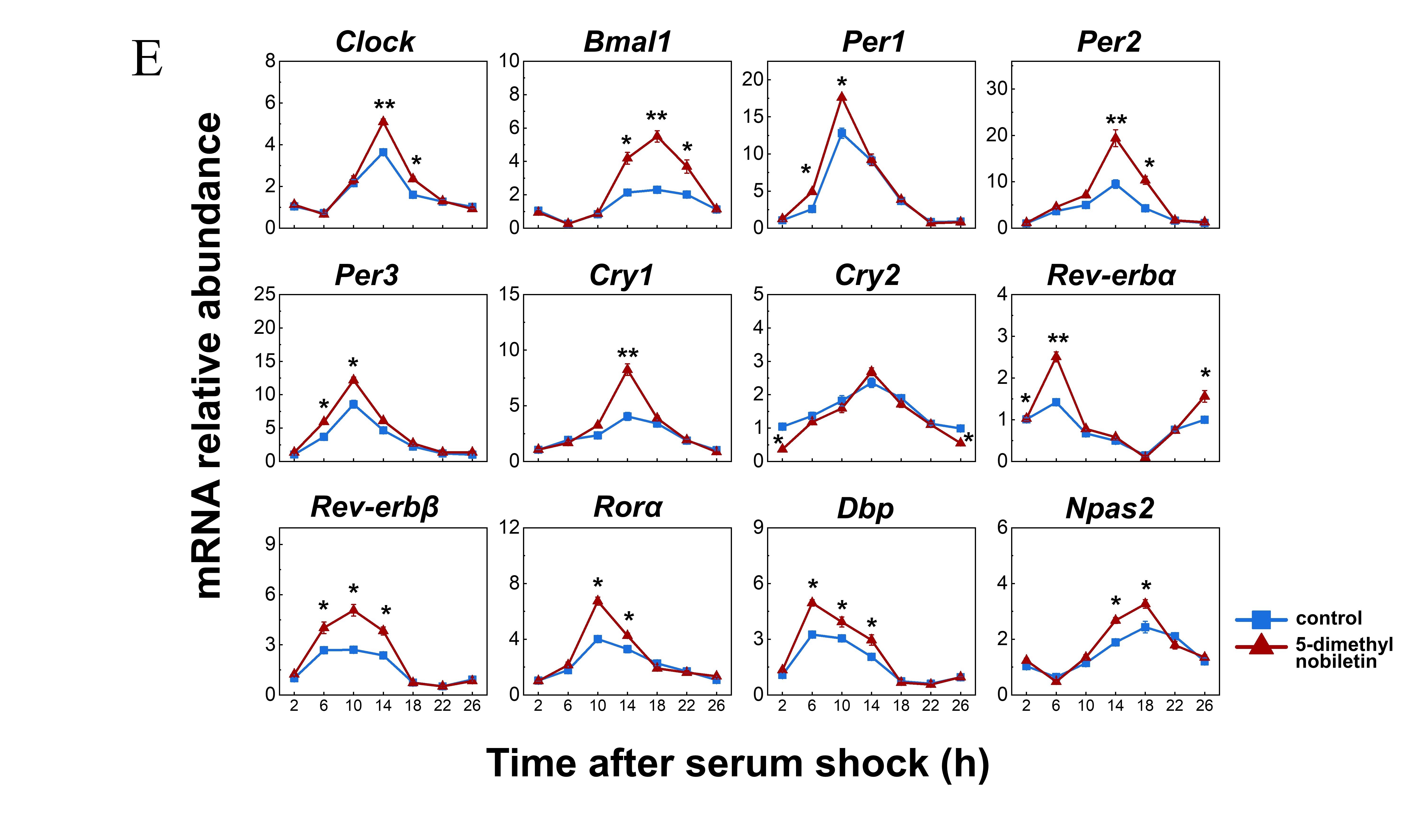

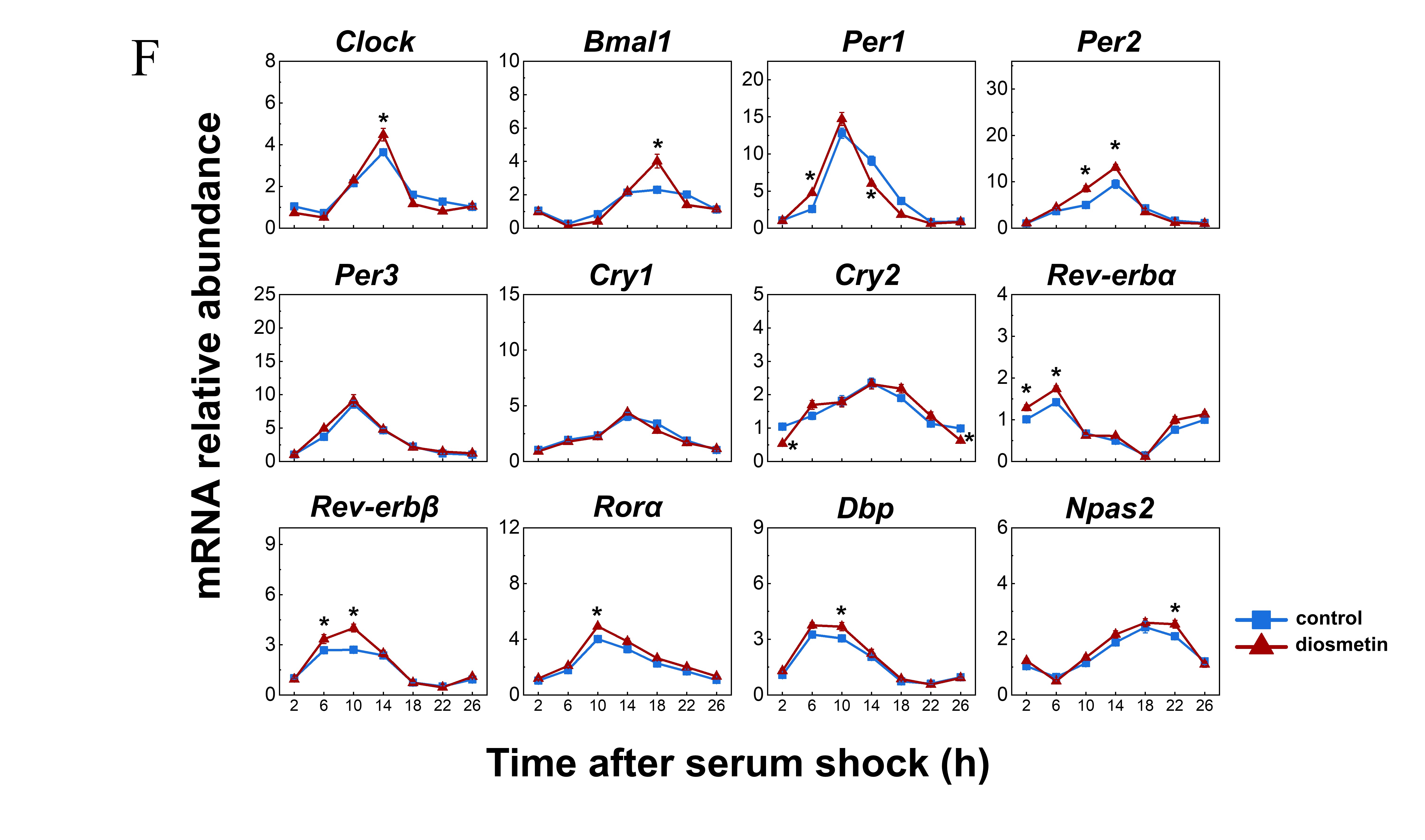

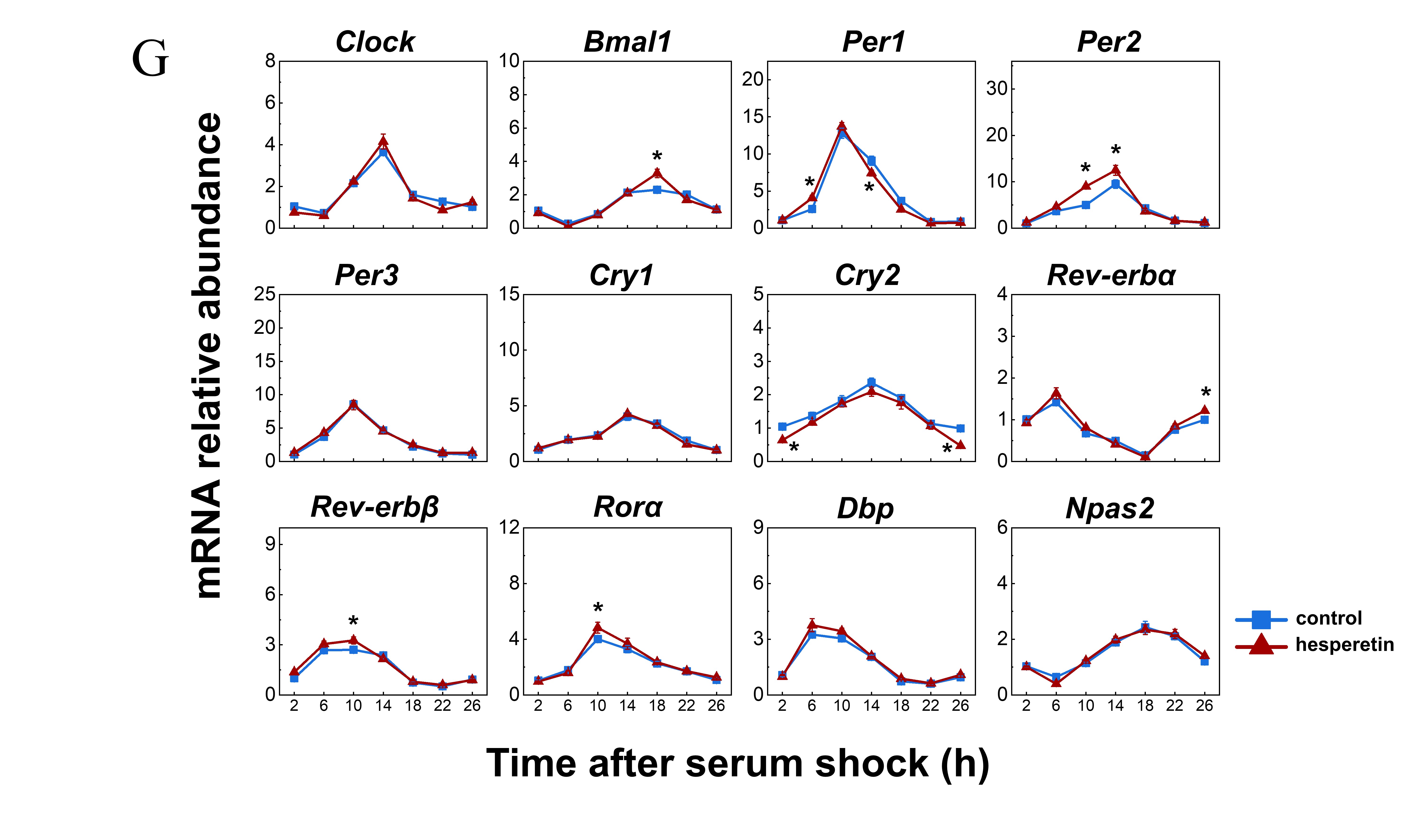

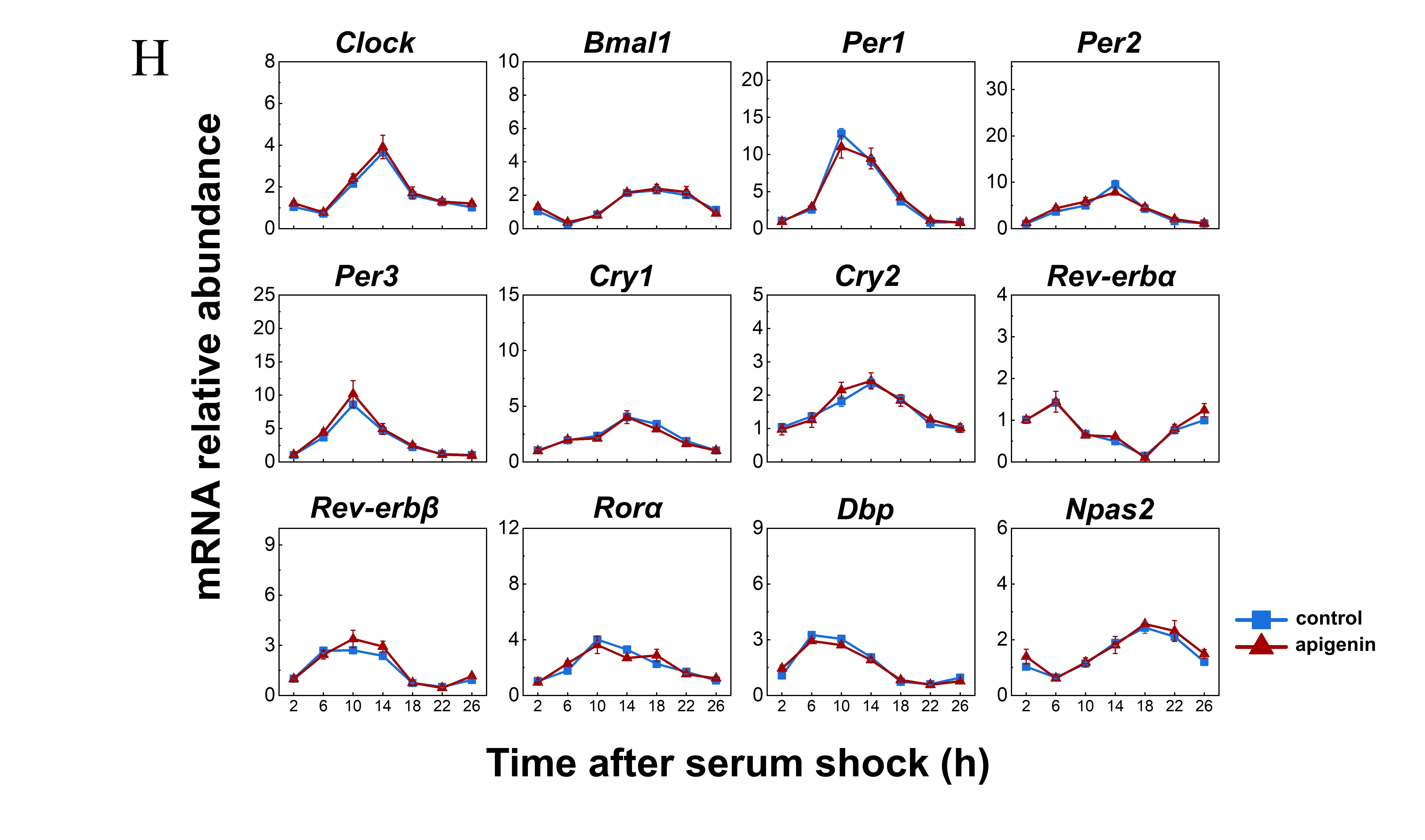

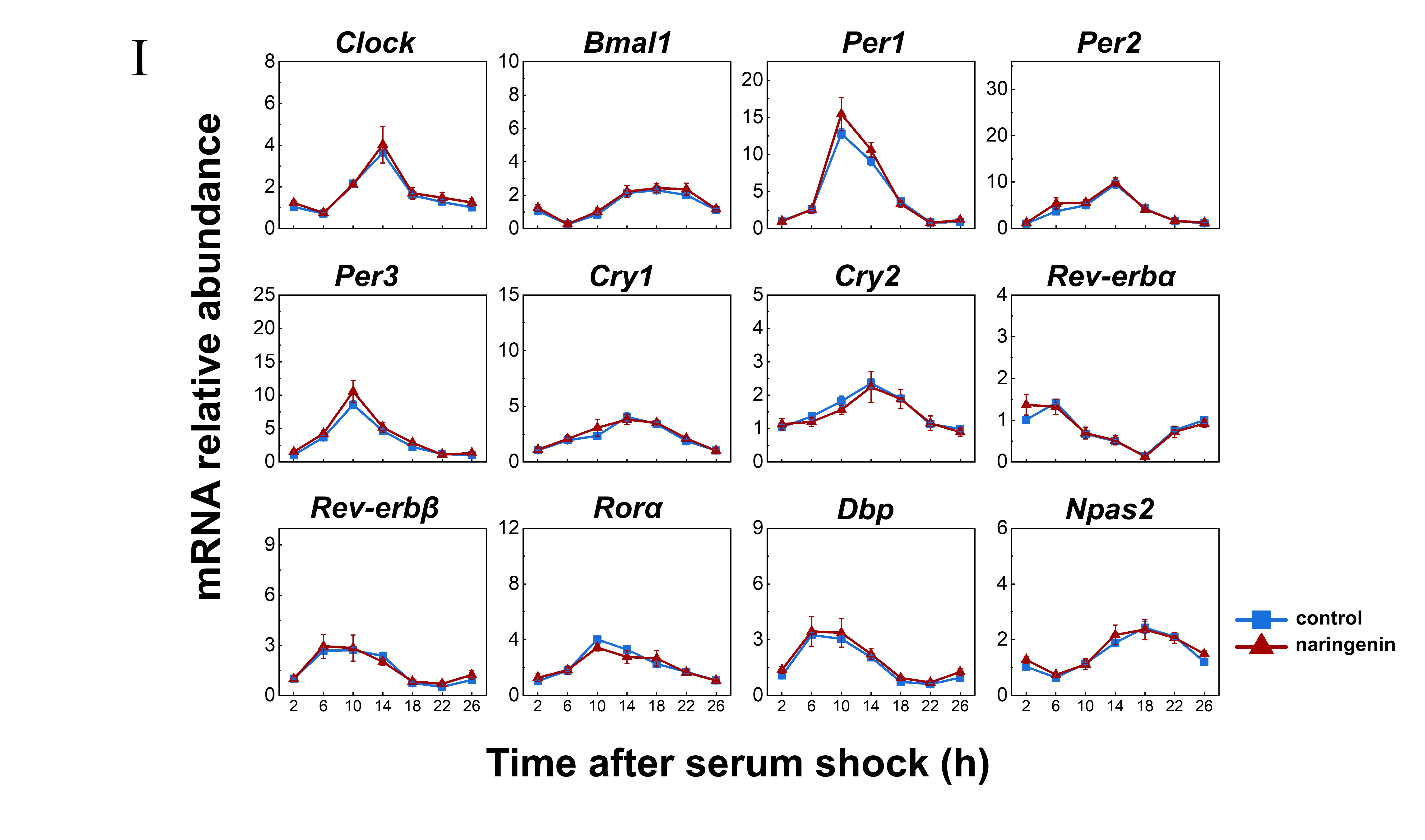

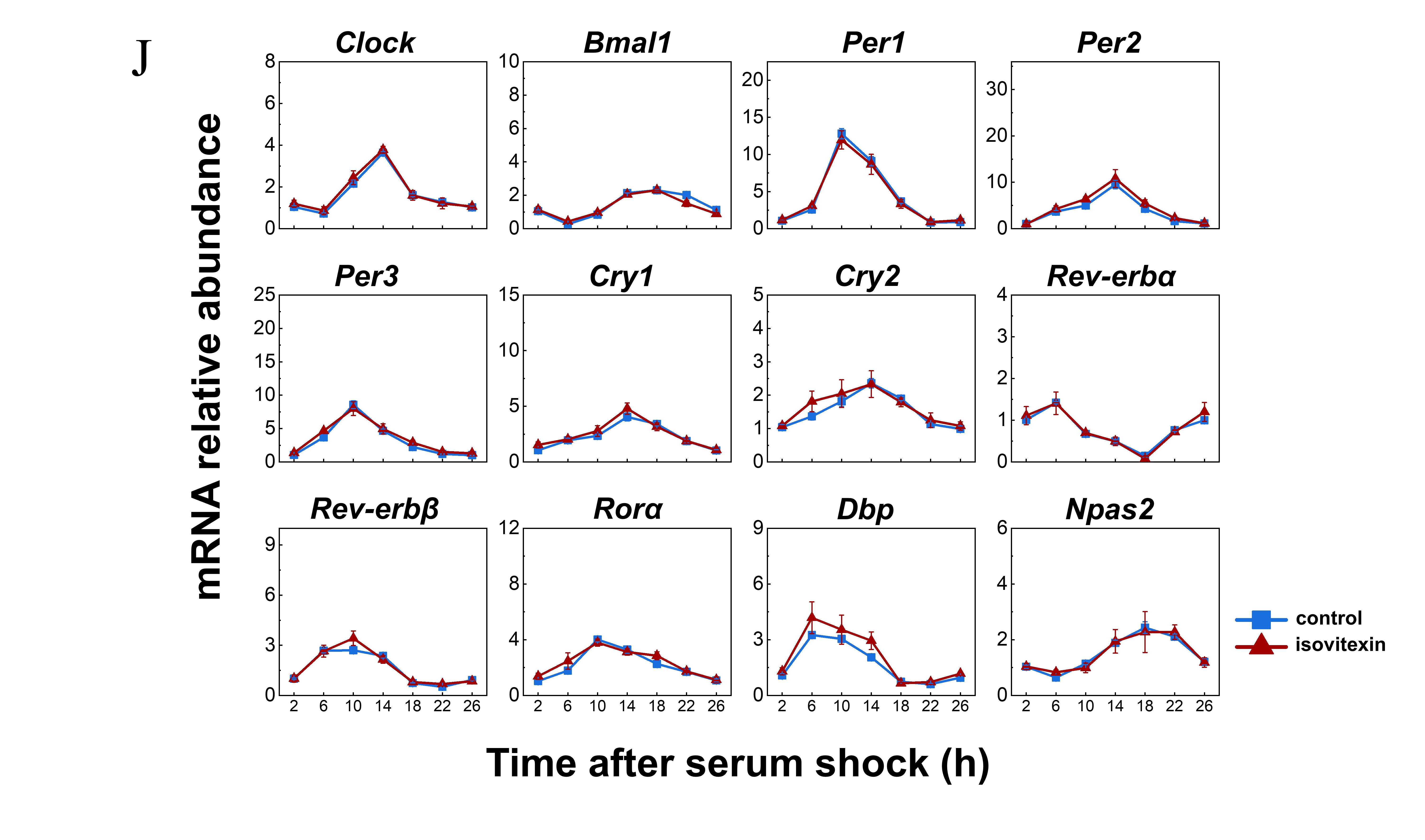

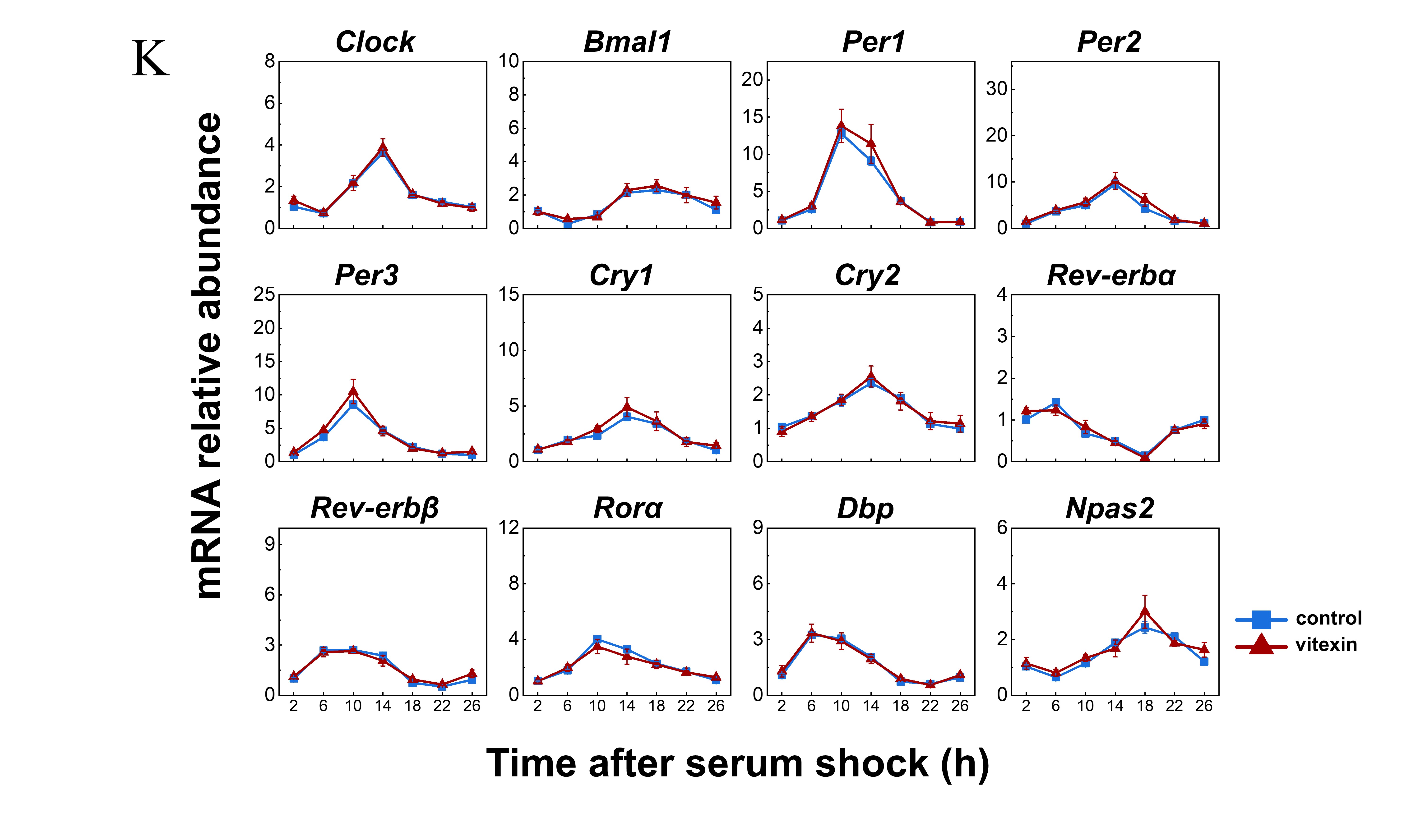

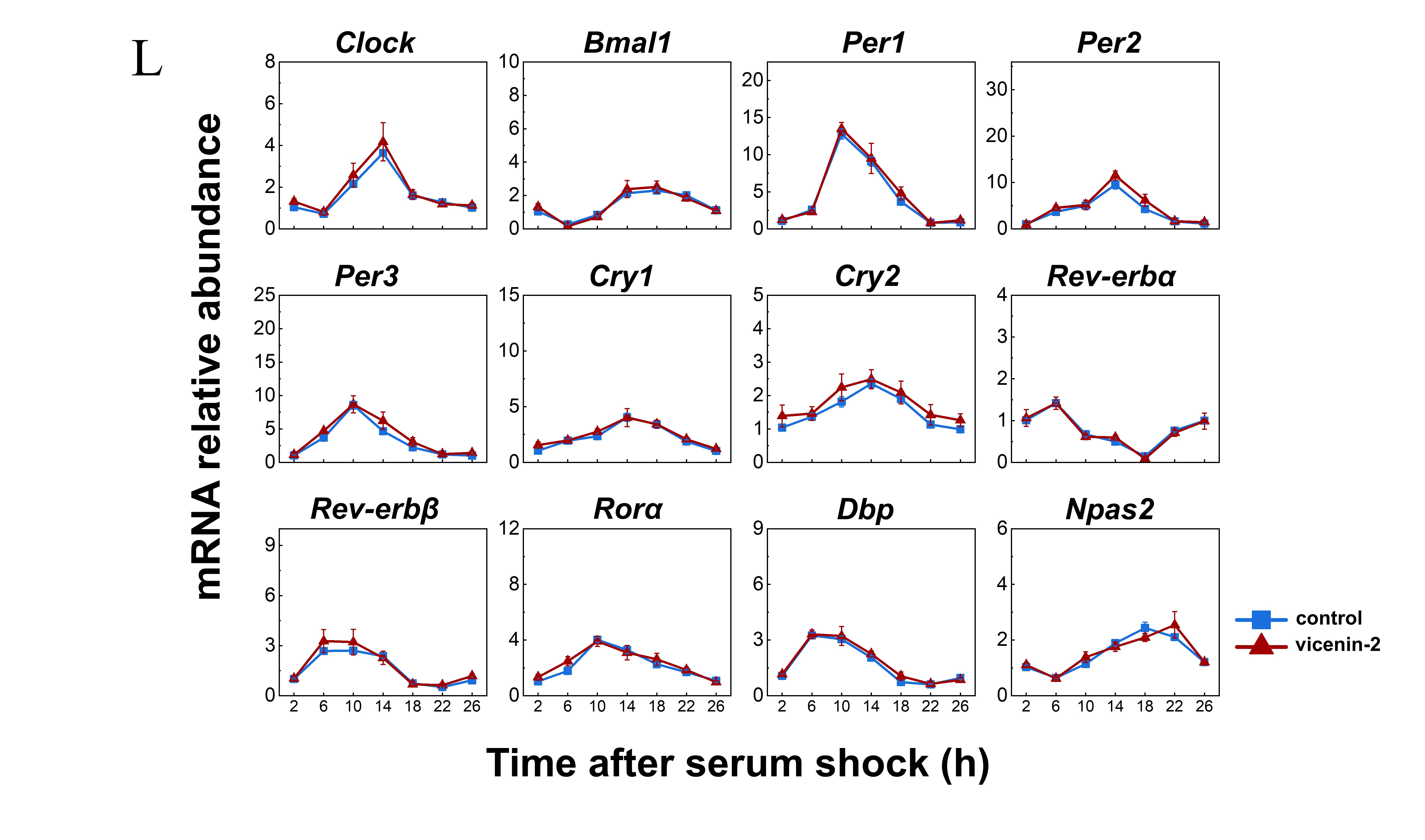

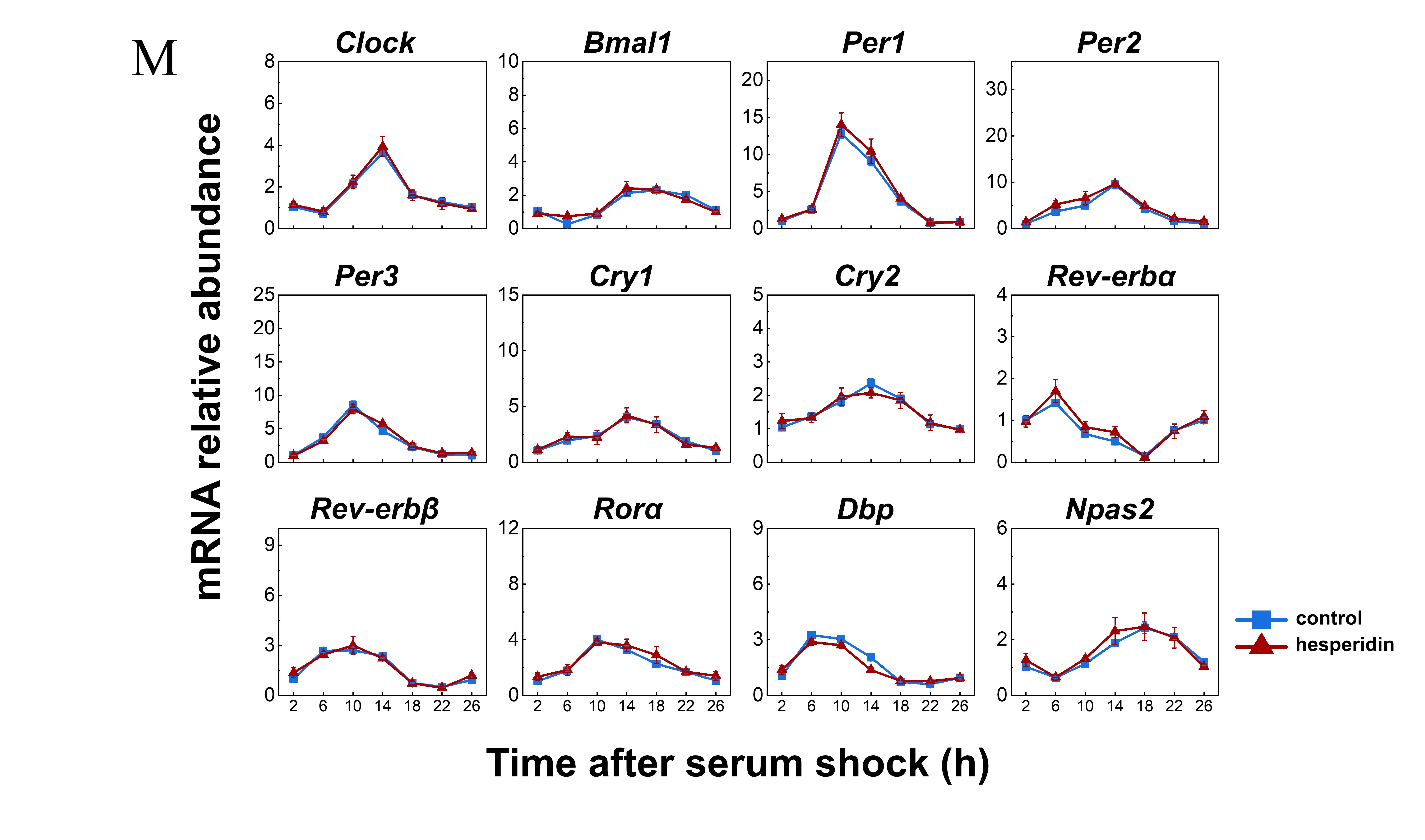

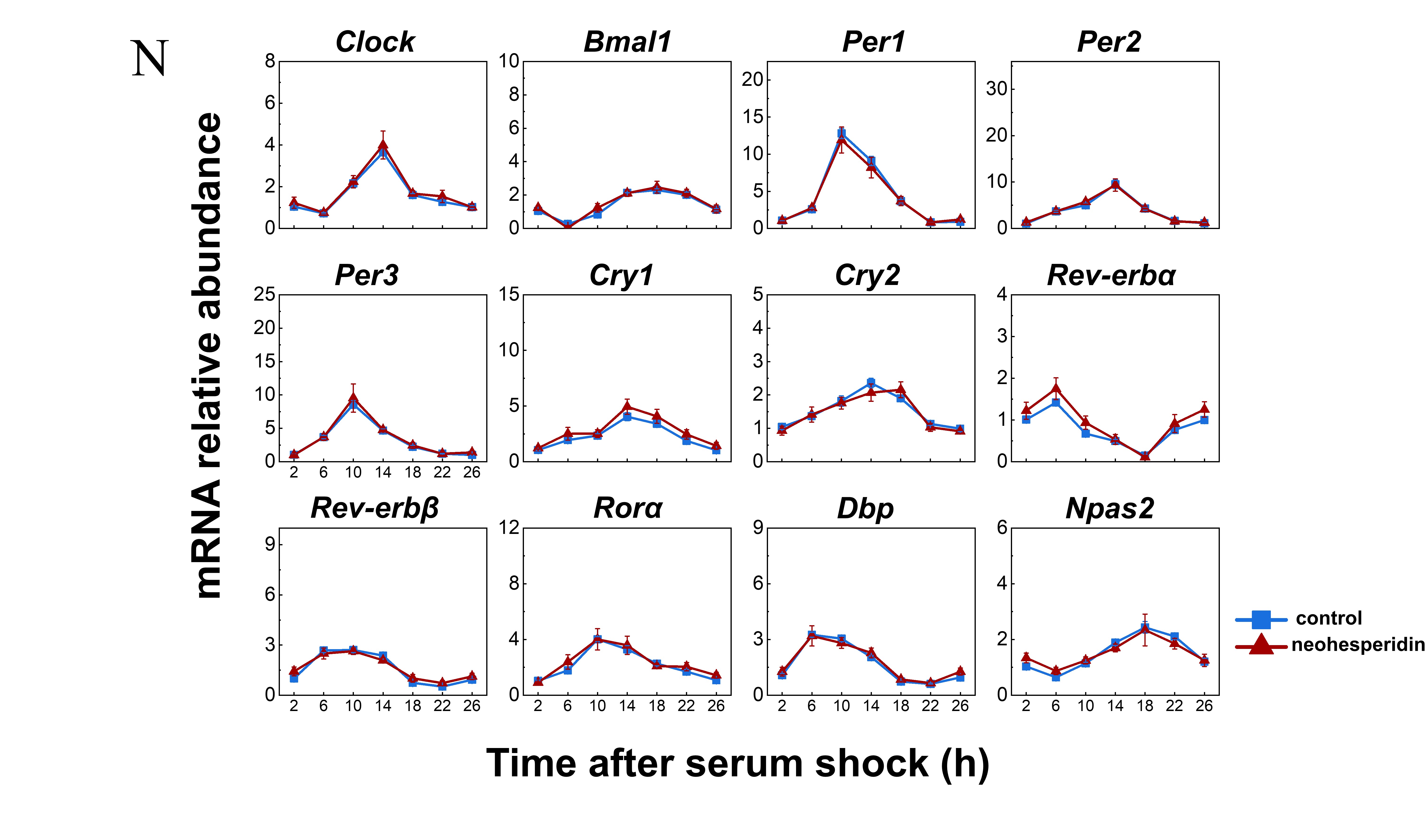

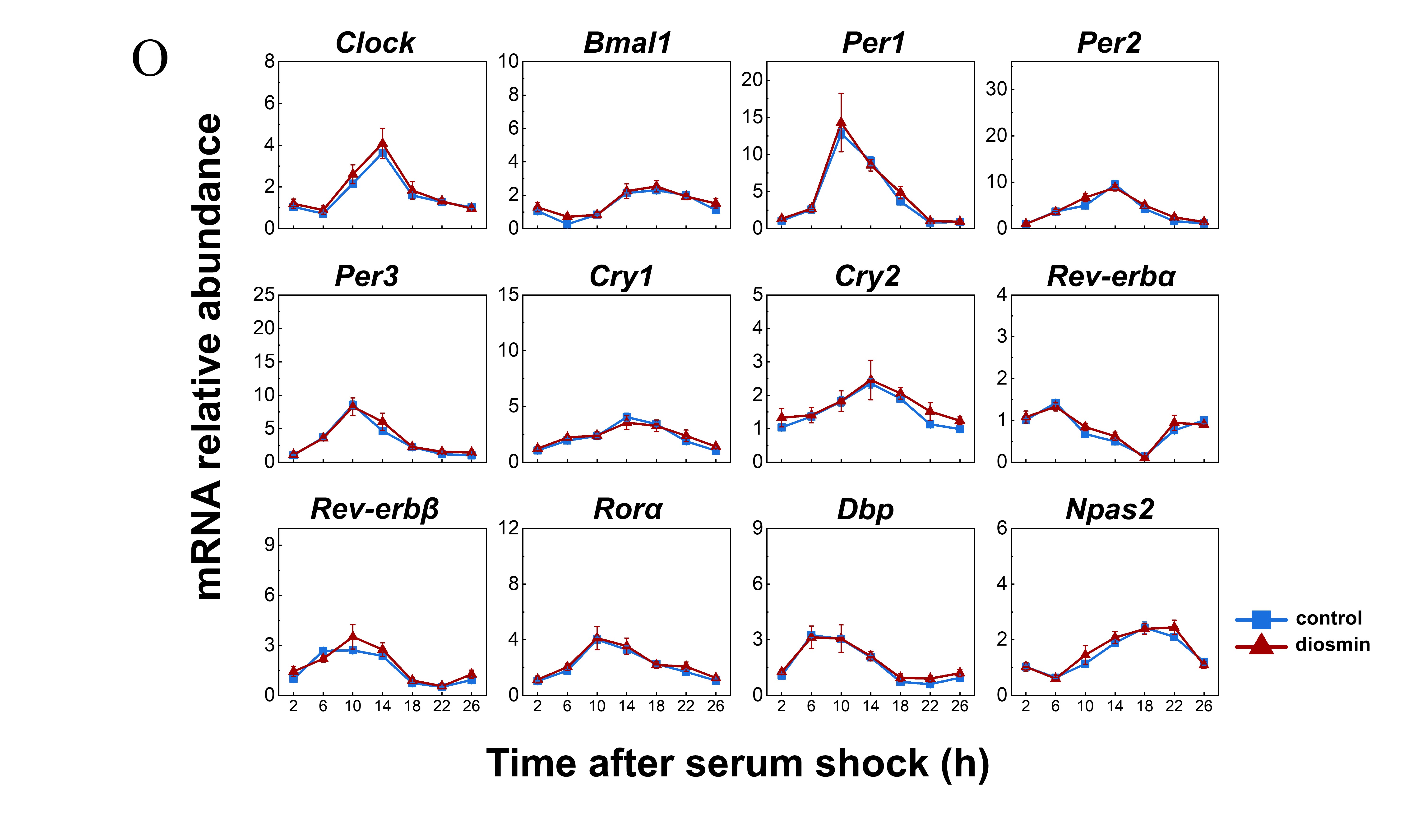

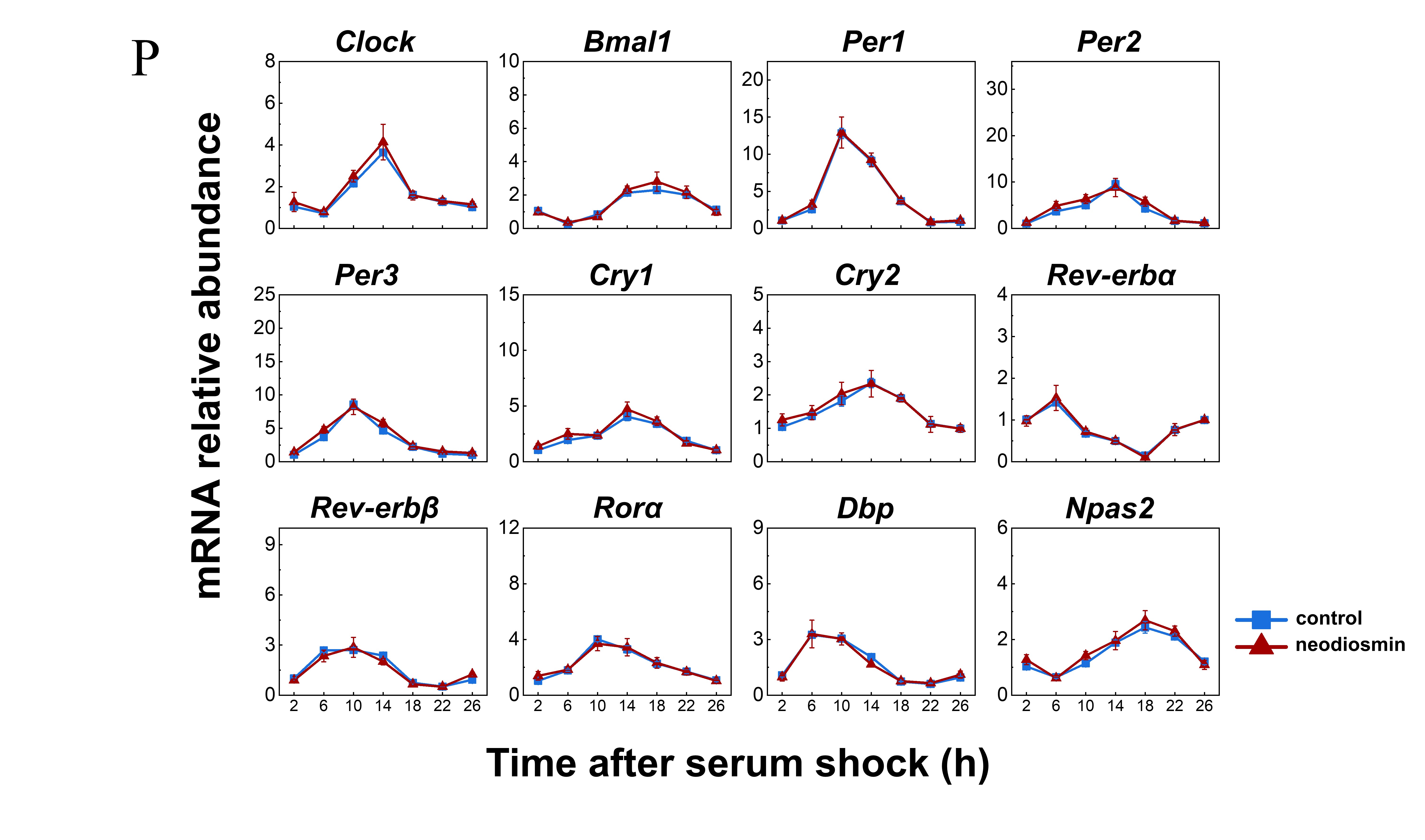

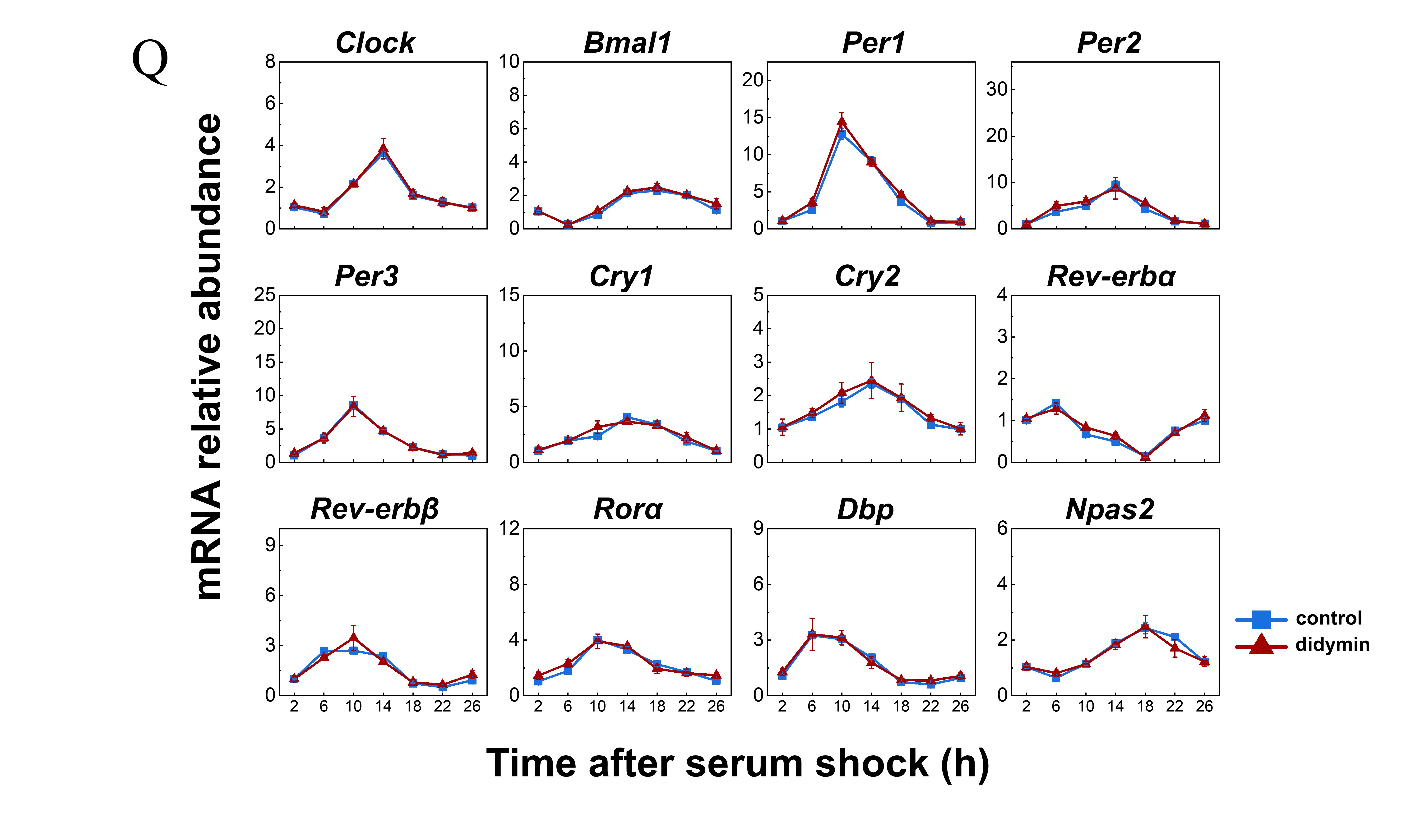

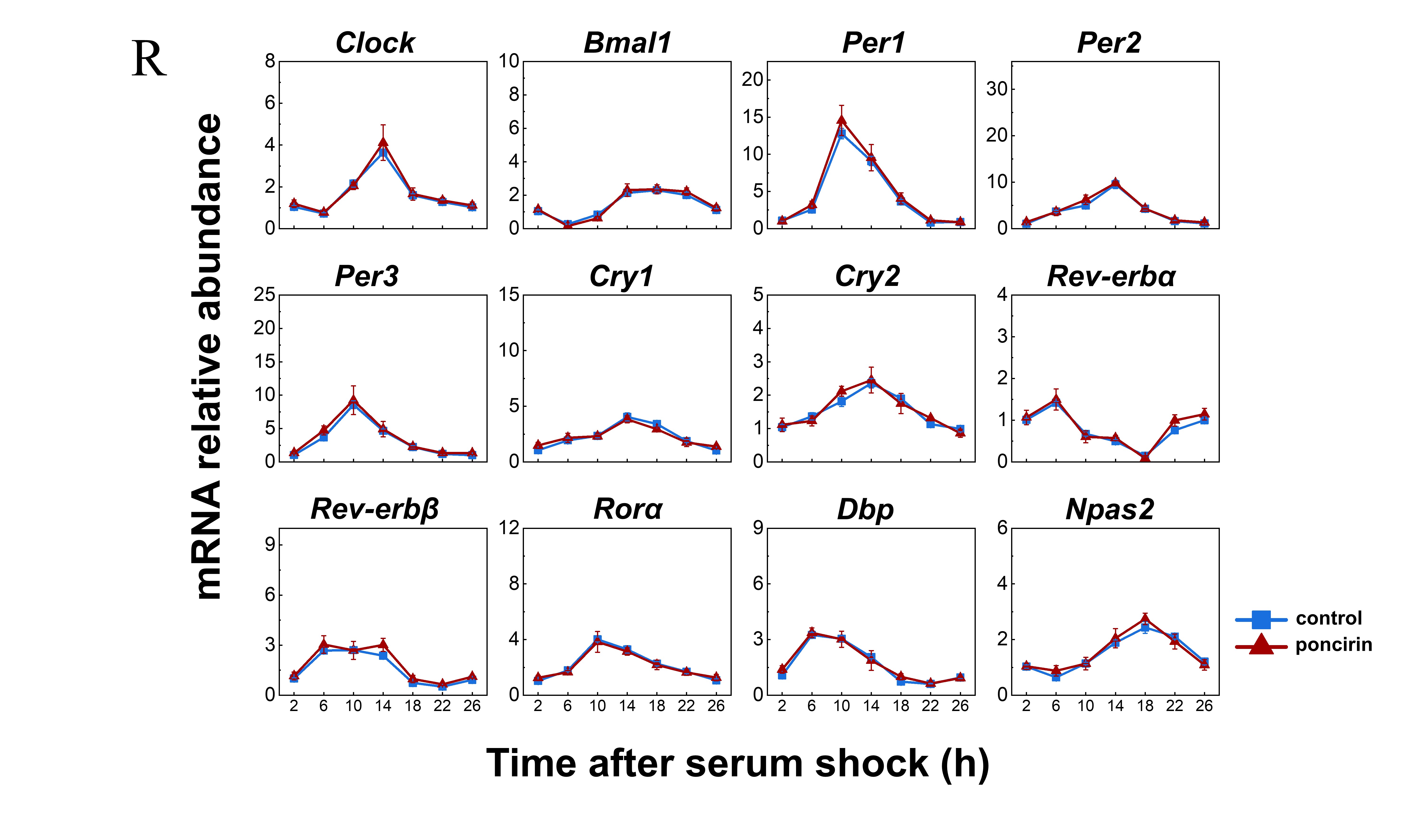

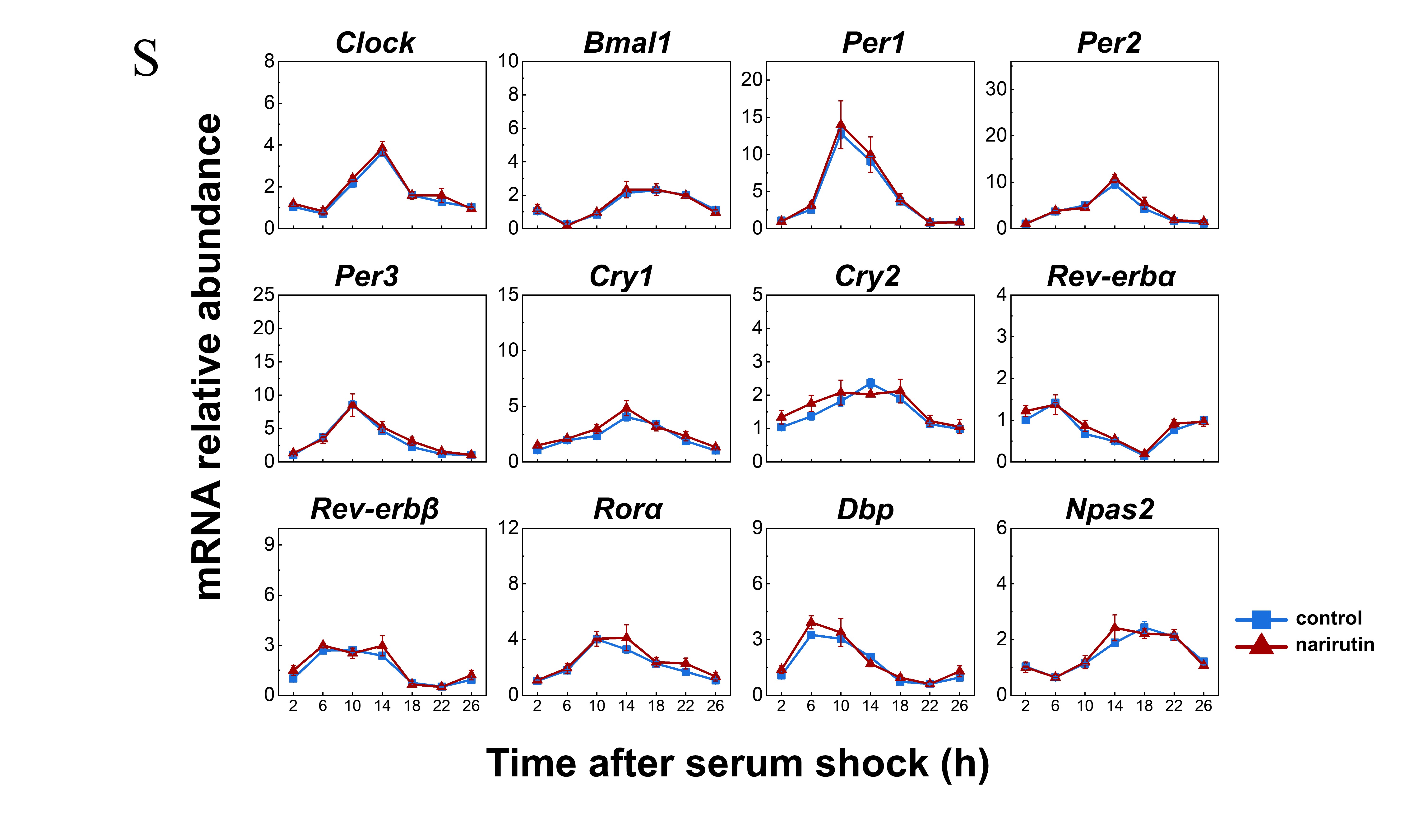

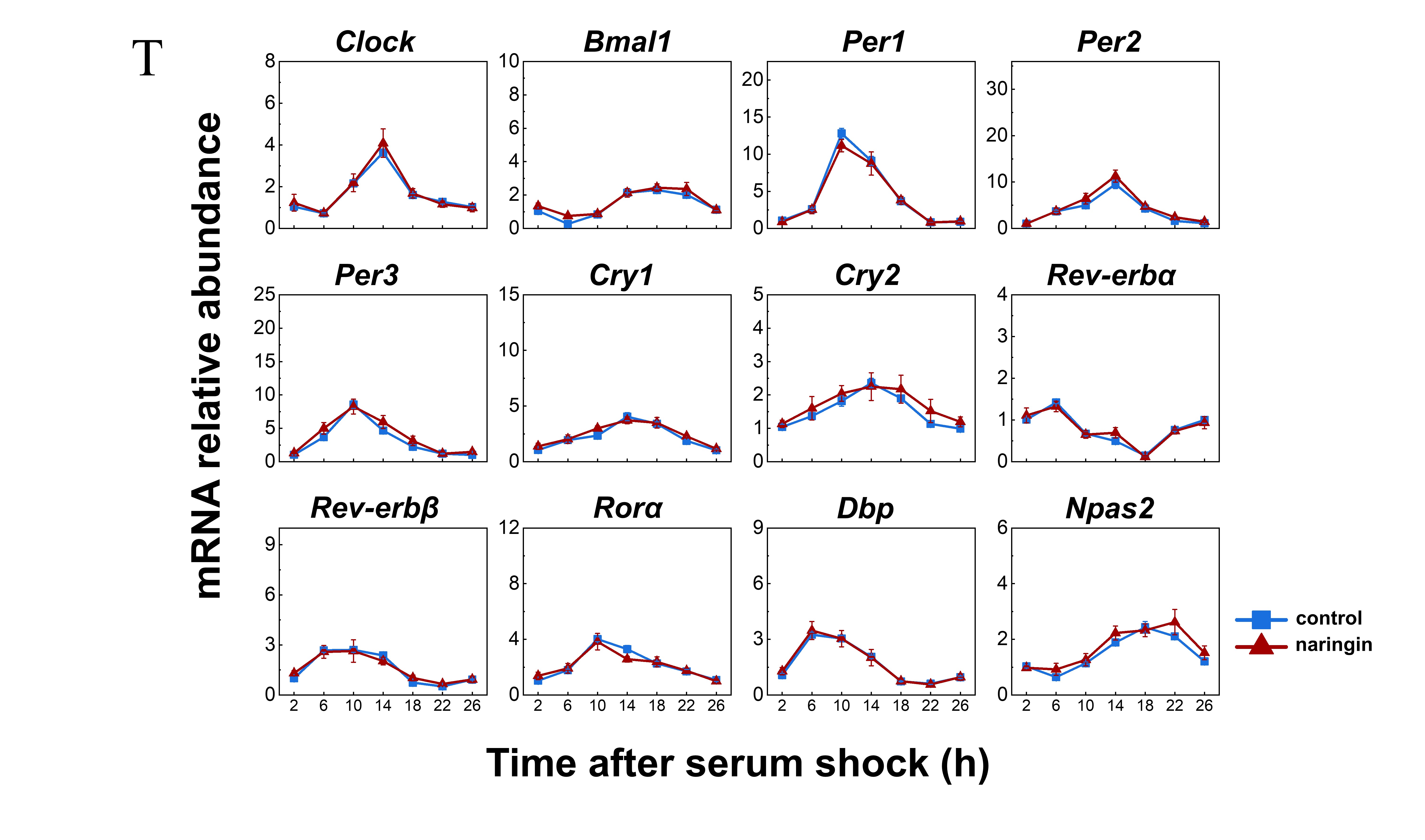


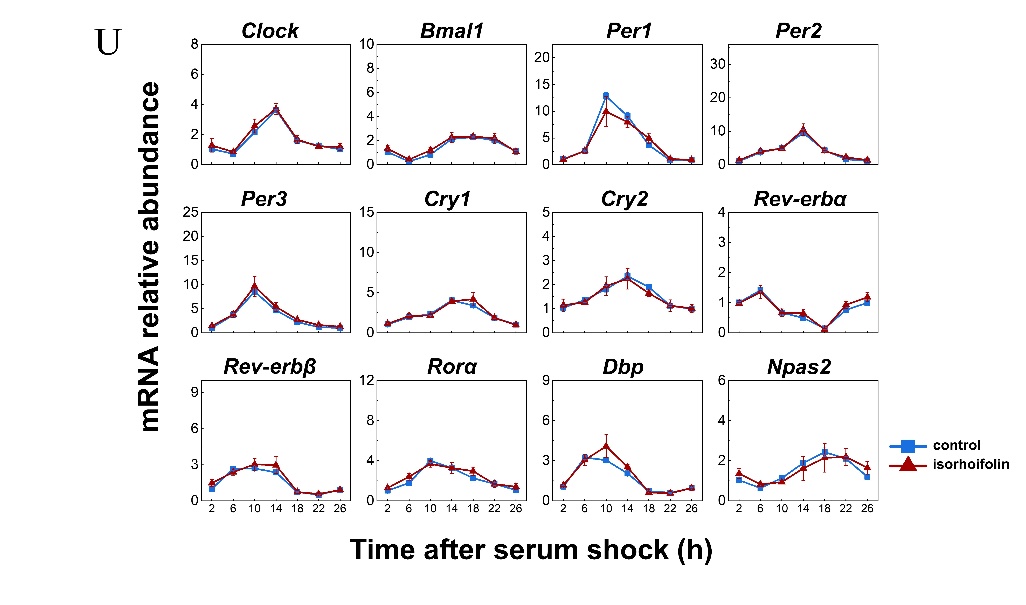


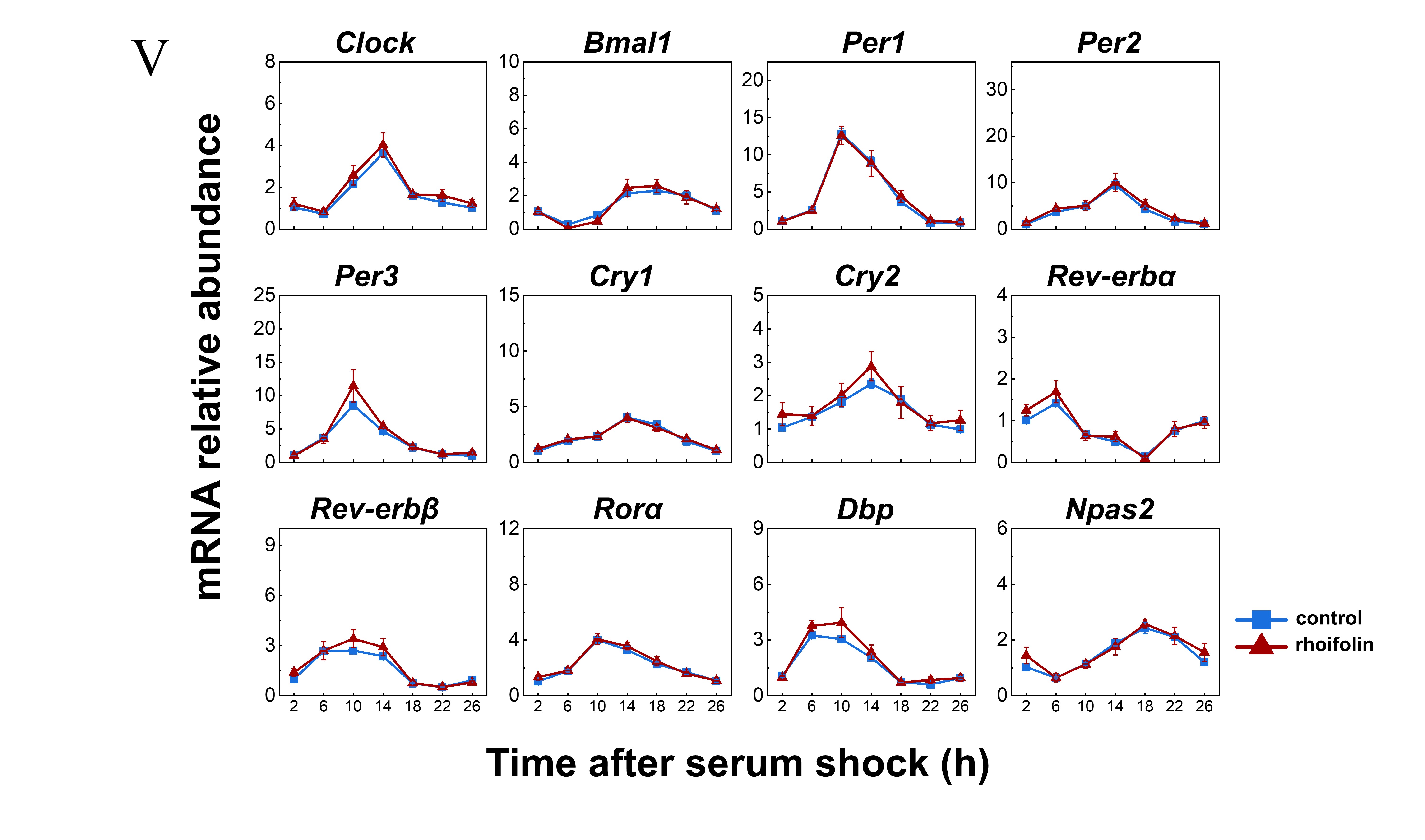

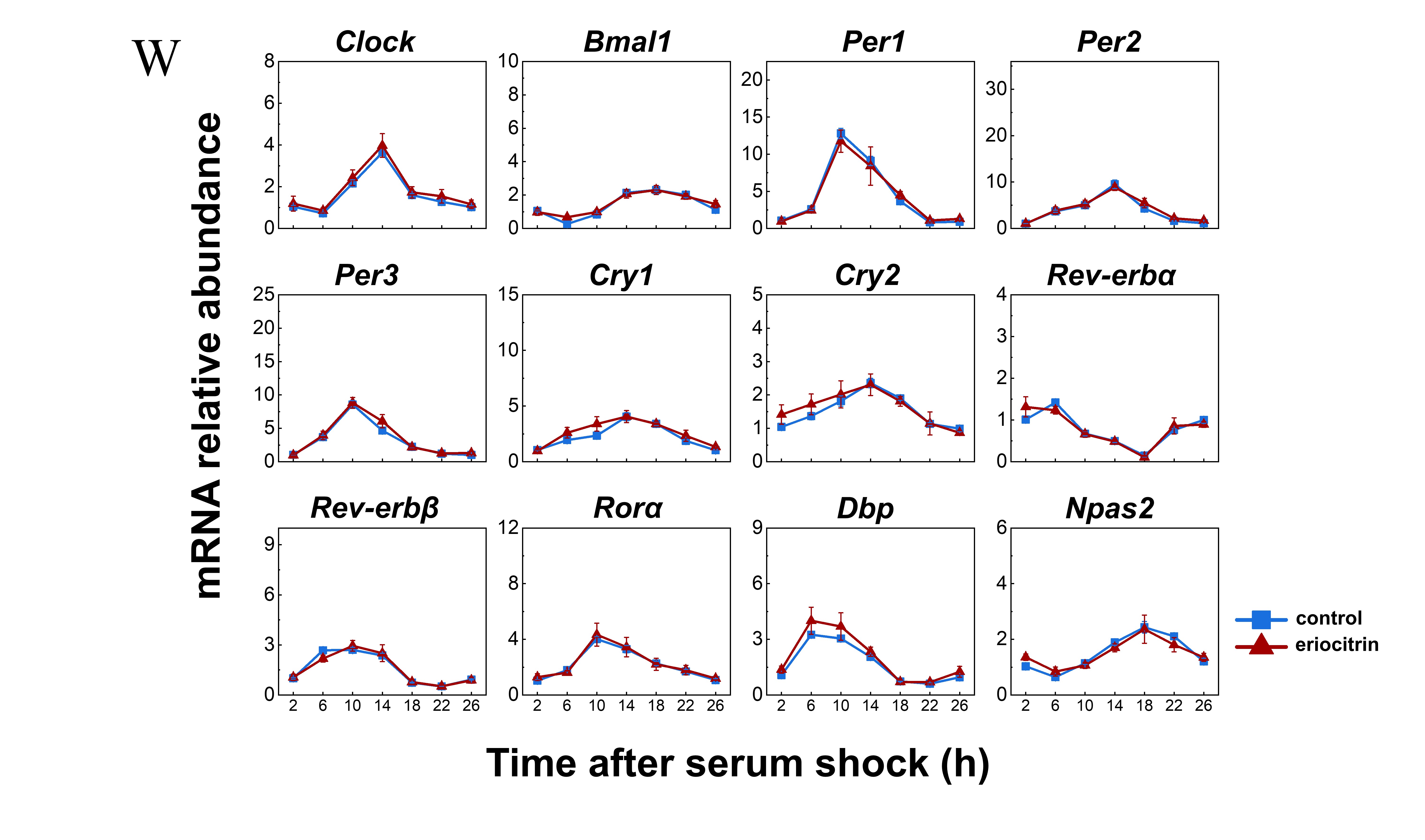

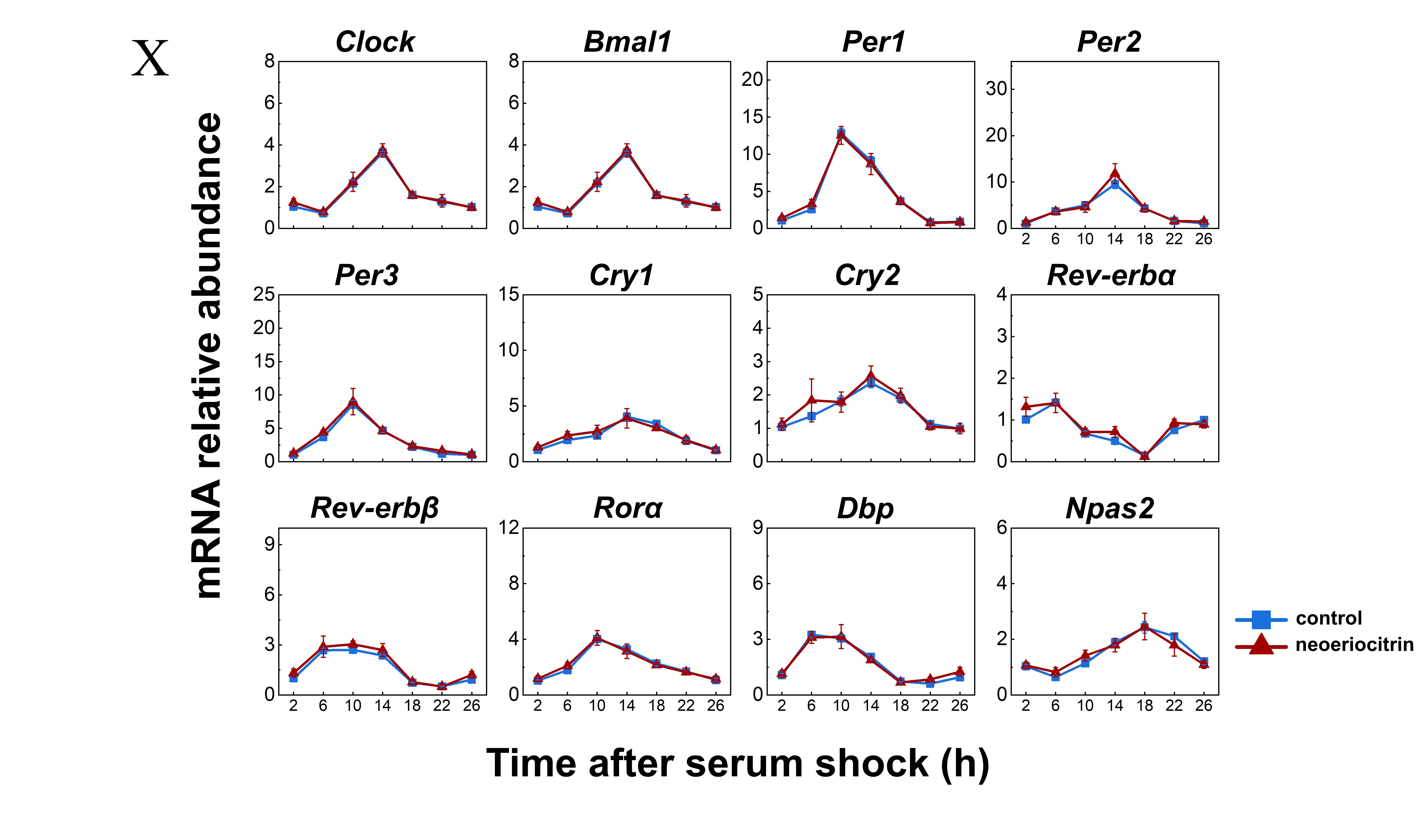


**Figure S2** Effects of flavonoid pretreatment on the expression of circadian genes, *Clock*, *Bmal1*, *Per1*, *Per2*, *Per3*, *Cry1*, *Cry2*, *Rev-erbα*, *Rev-erbβ*, *Rorα*, *Dbp,* and *Npas2*, in BV-2 cells. Relative mRNA levels were measured using qRT-PCR. The pretreatment concentration was: 10 μM for sinensetin, isosinensetin, tangeretin, 5-demethylnobiletin, and gardenin B; 160 μM for diosmetin, hesperetin, agpigenin and naringein, 320 μM for isovitexin, vitexin, vicenin-2, hesperidin, diosmin, didymin, narirutin, isorhoifolin, and eriocitrin, neohesperidin, neodiosmin, poncirin, naringin, rhoifolin, and neoeriocitrin. Data is presented as the mean ± standard deviation (n=3). * *p* < 0.05, ** *p* < 0.01, *** *p* < 0.001, compared to the DMSO blank control.

**Figure S3**


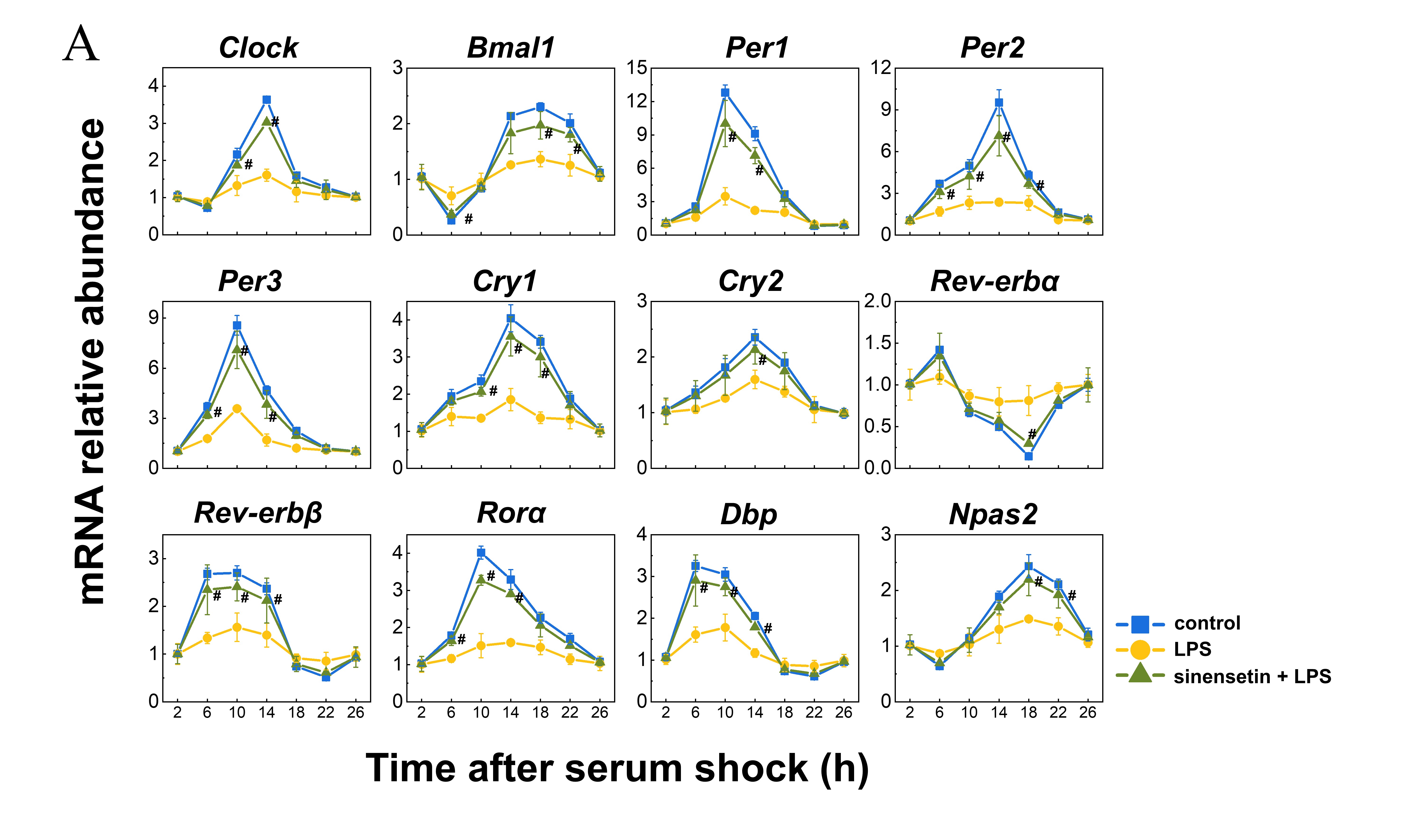

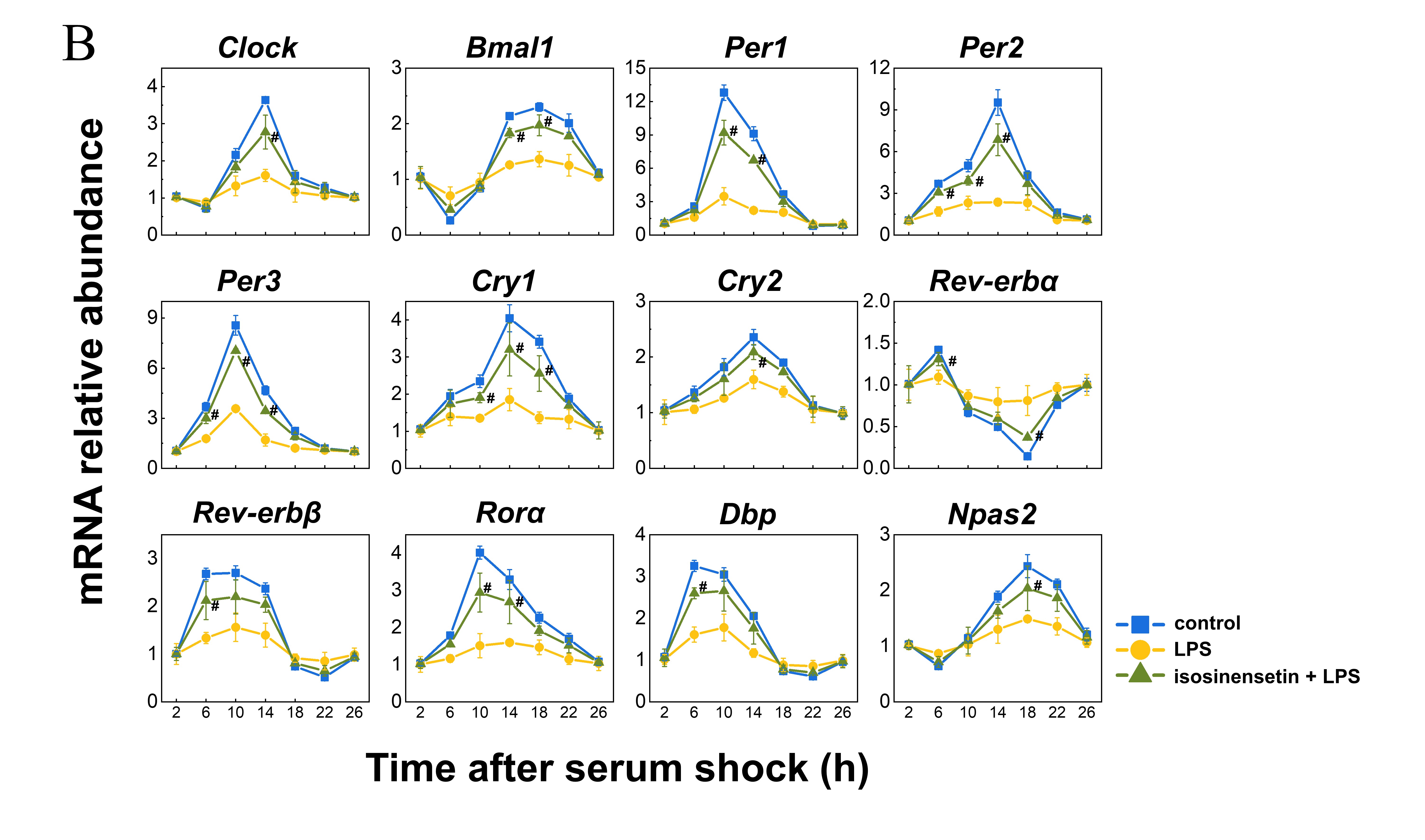

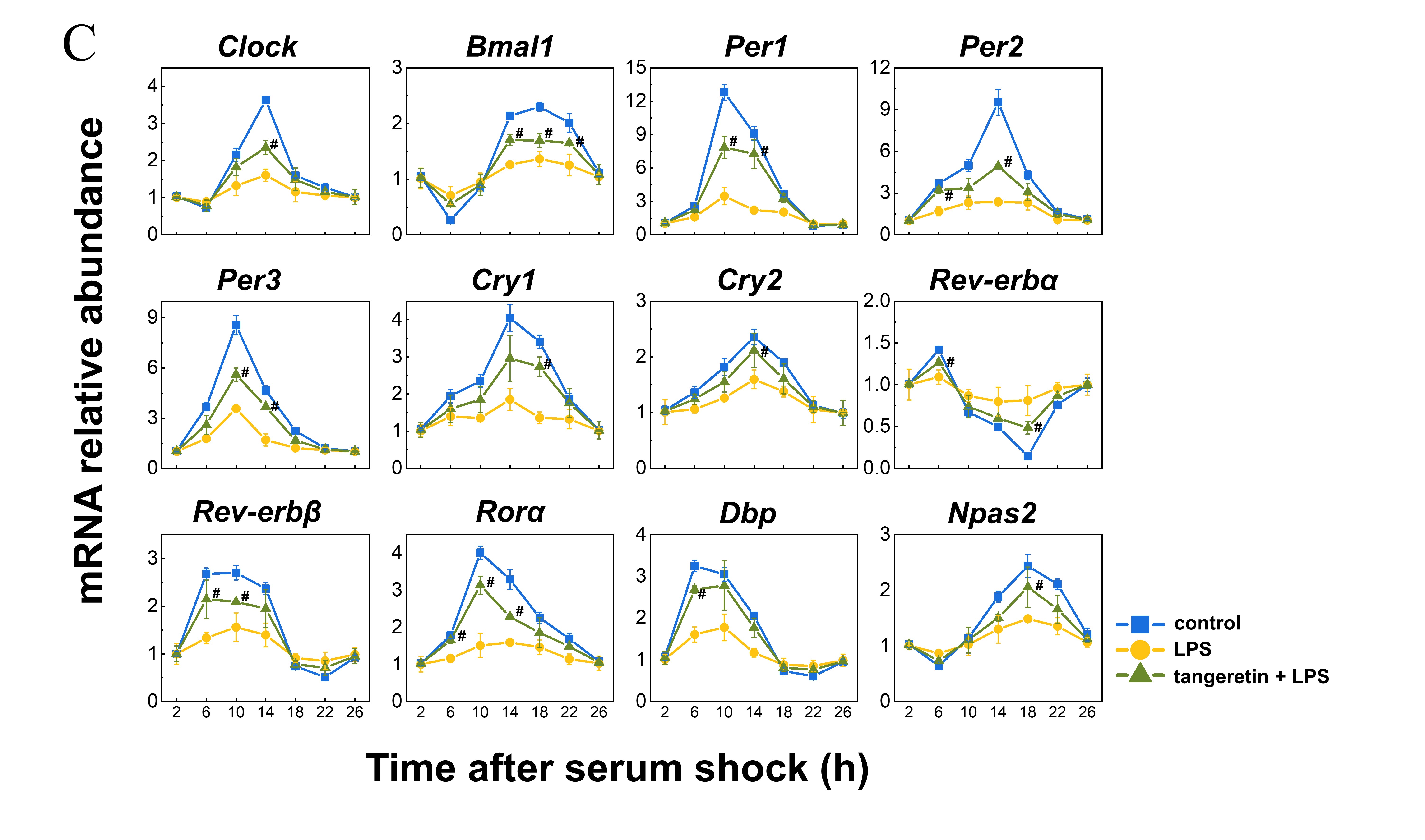

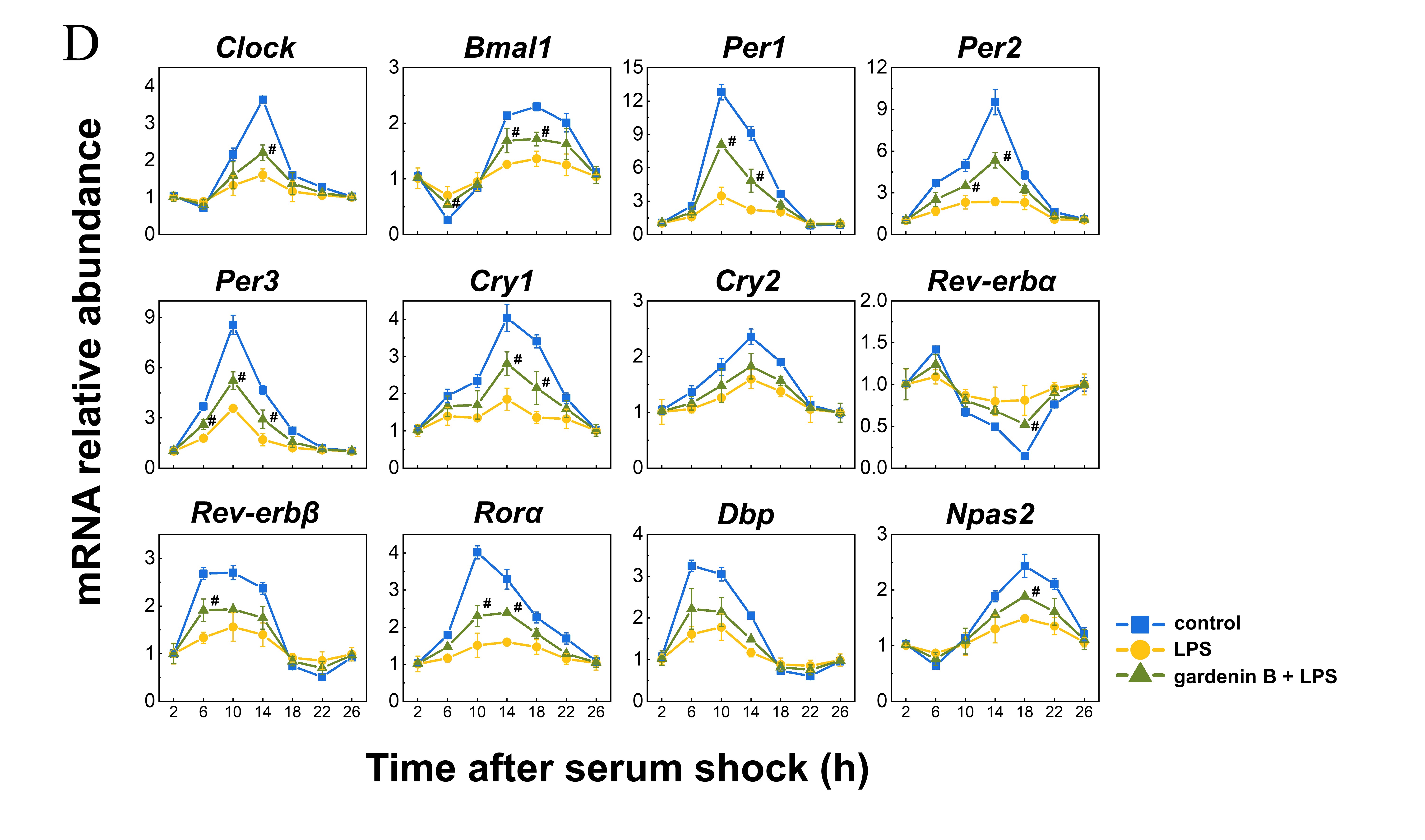

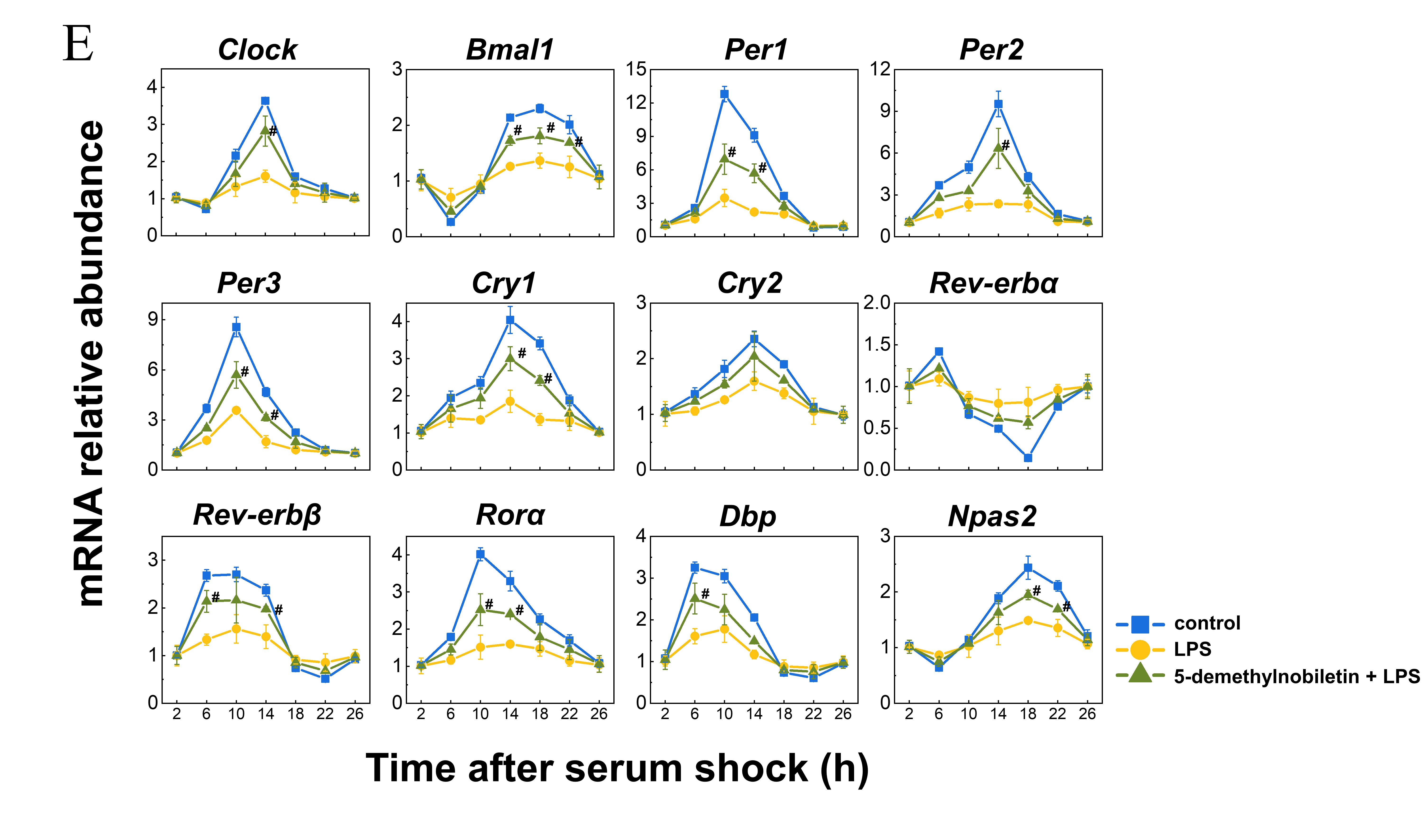

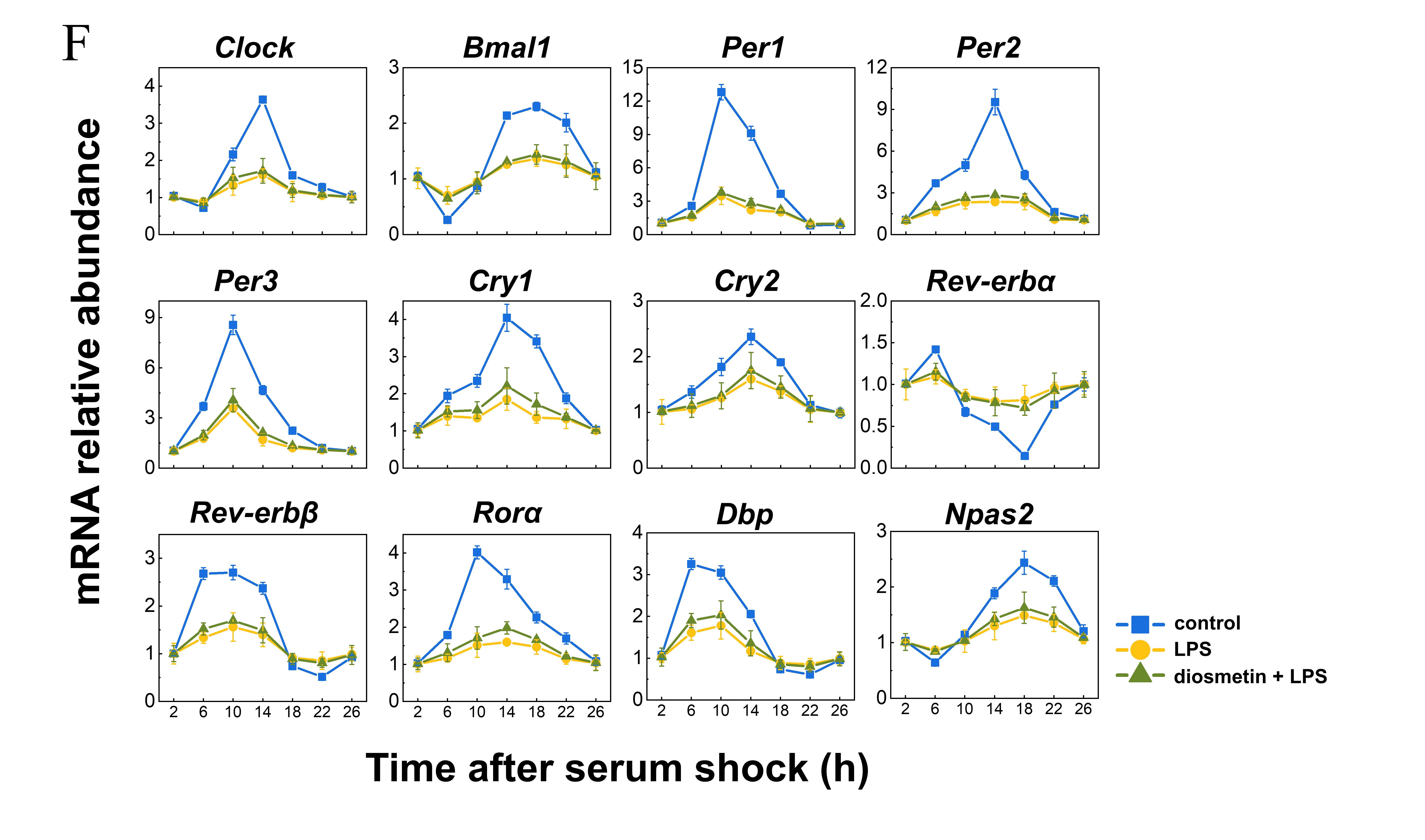

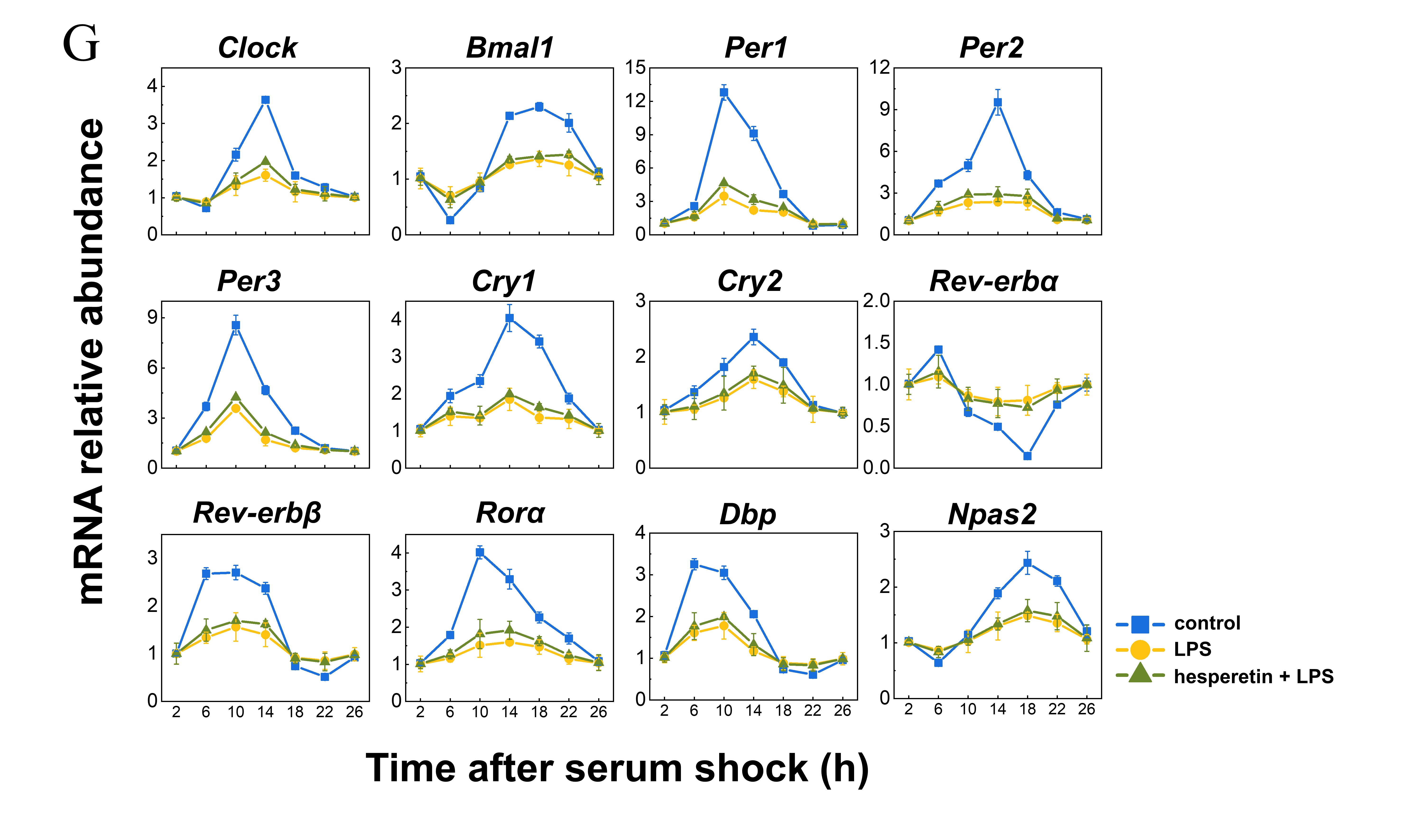

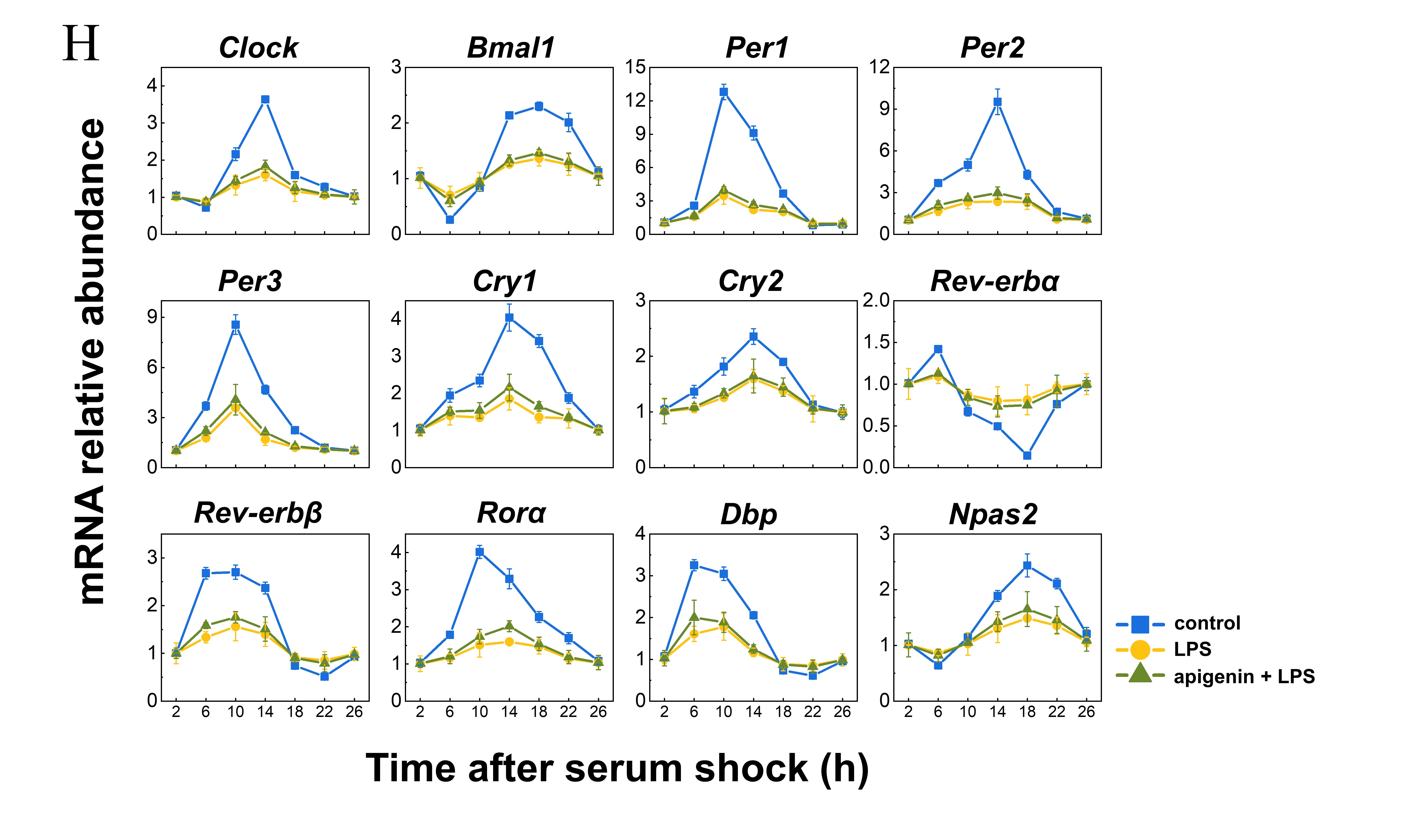


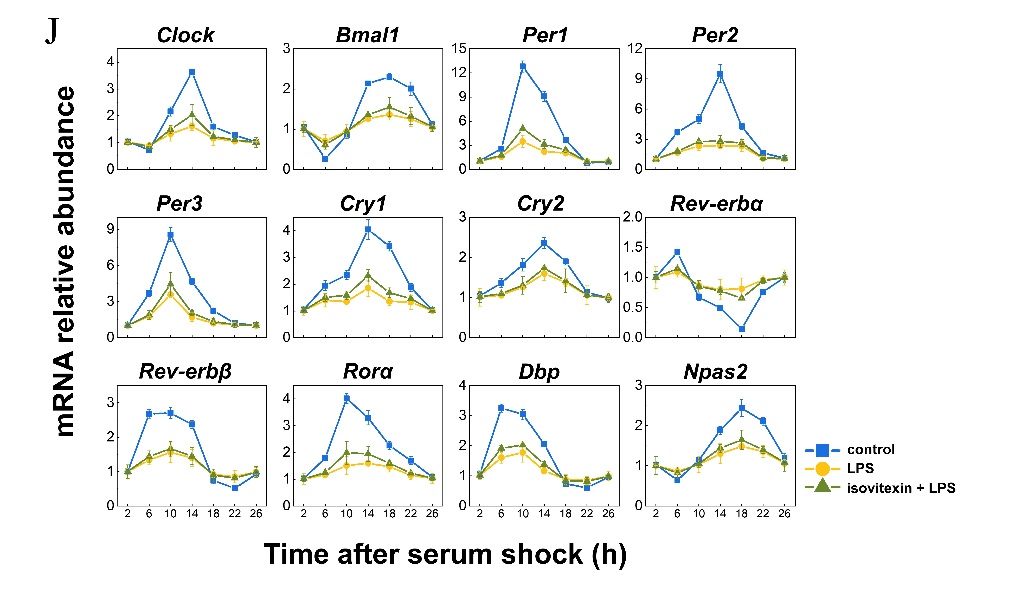


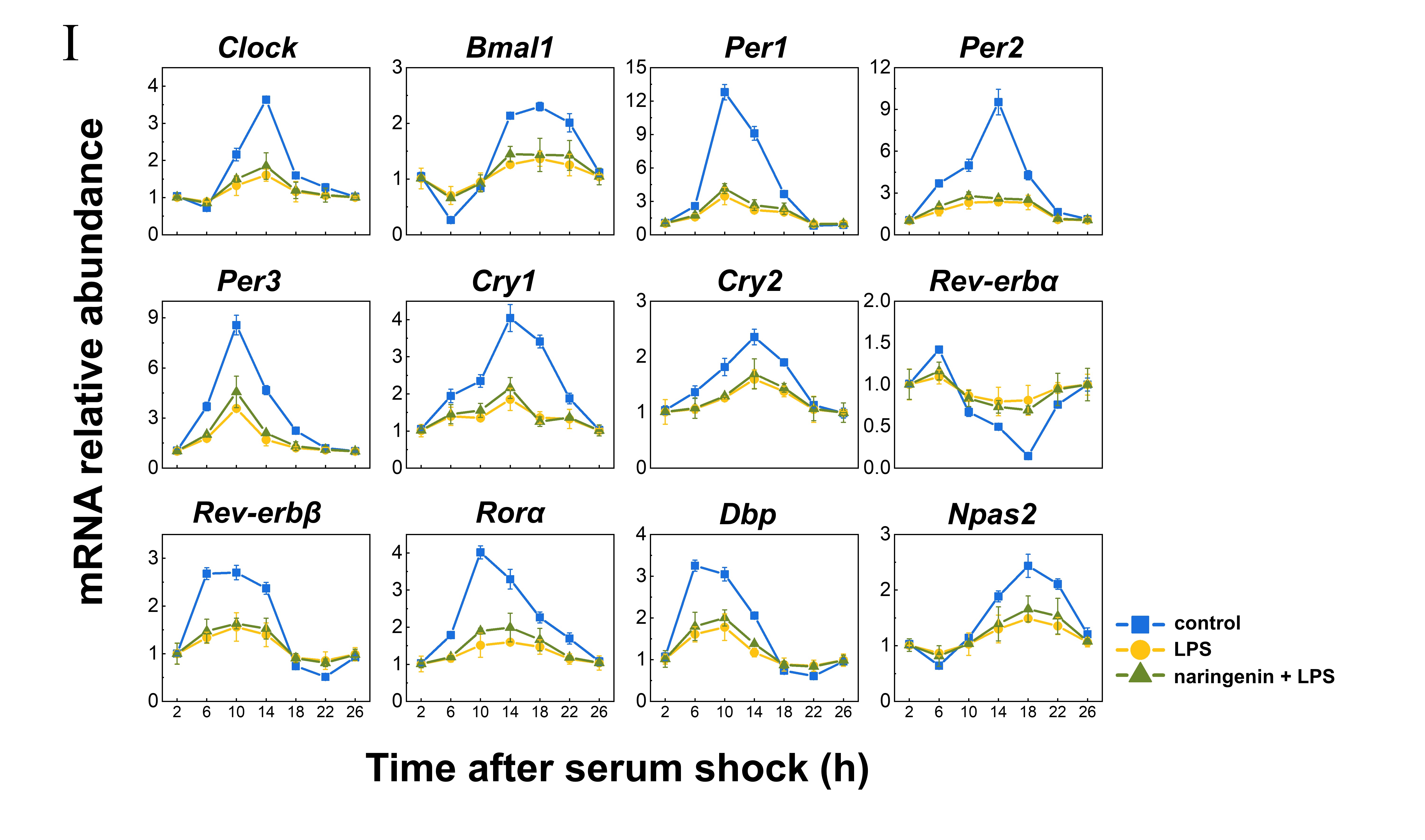


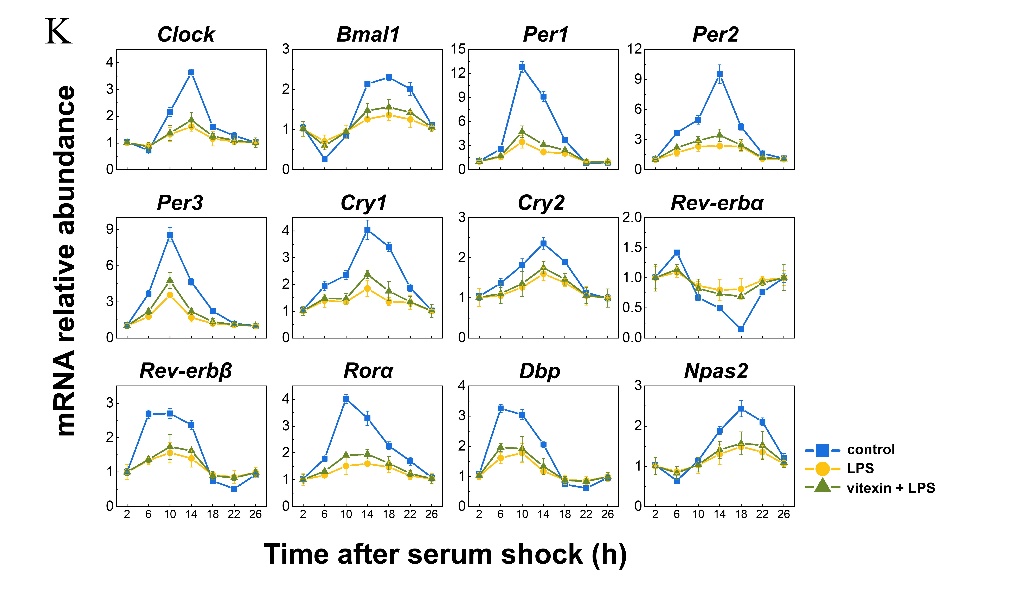


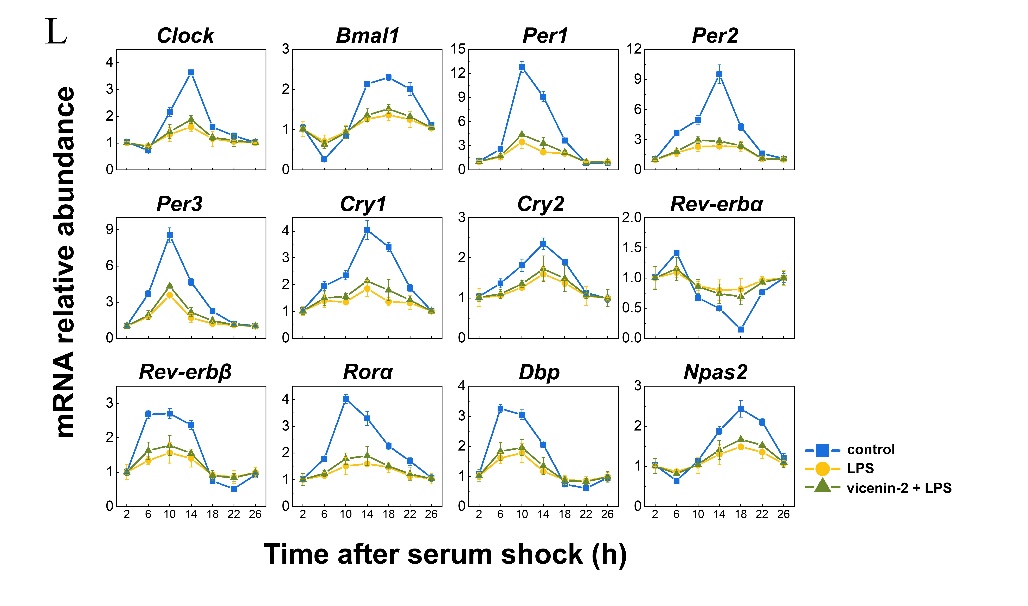


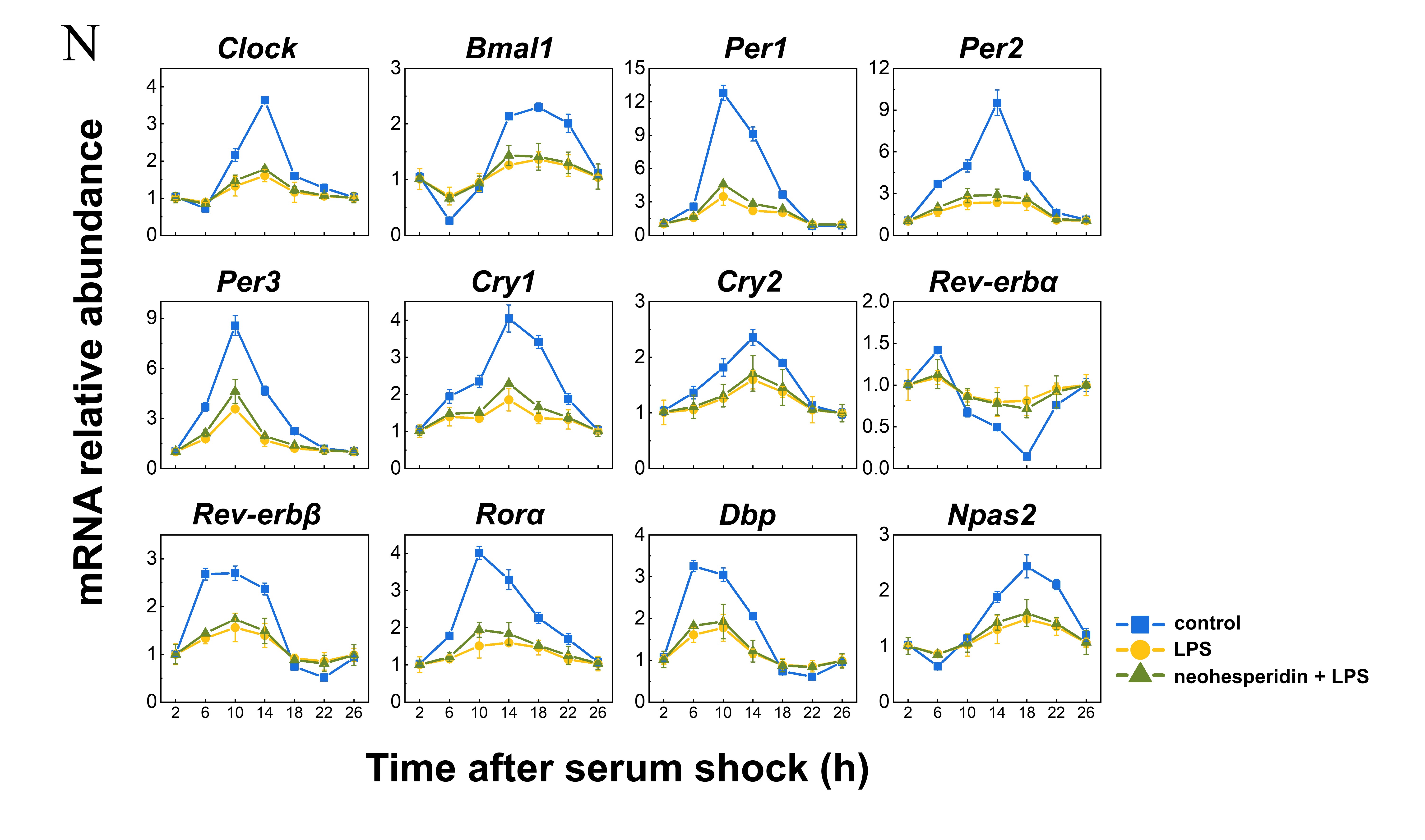

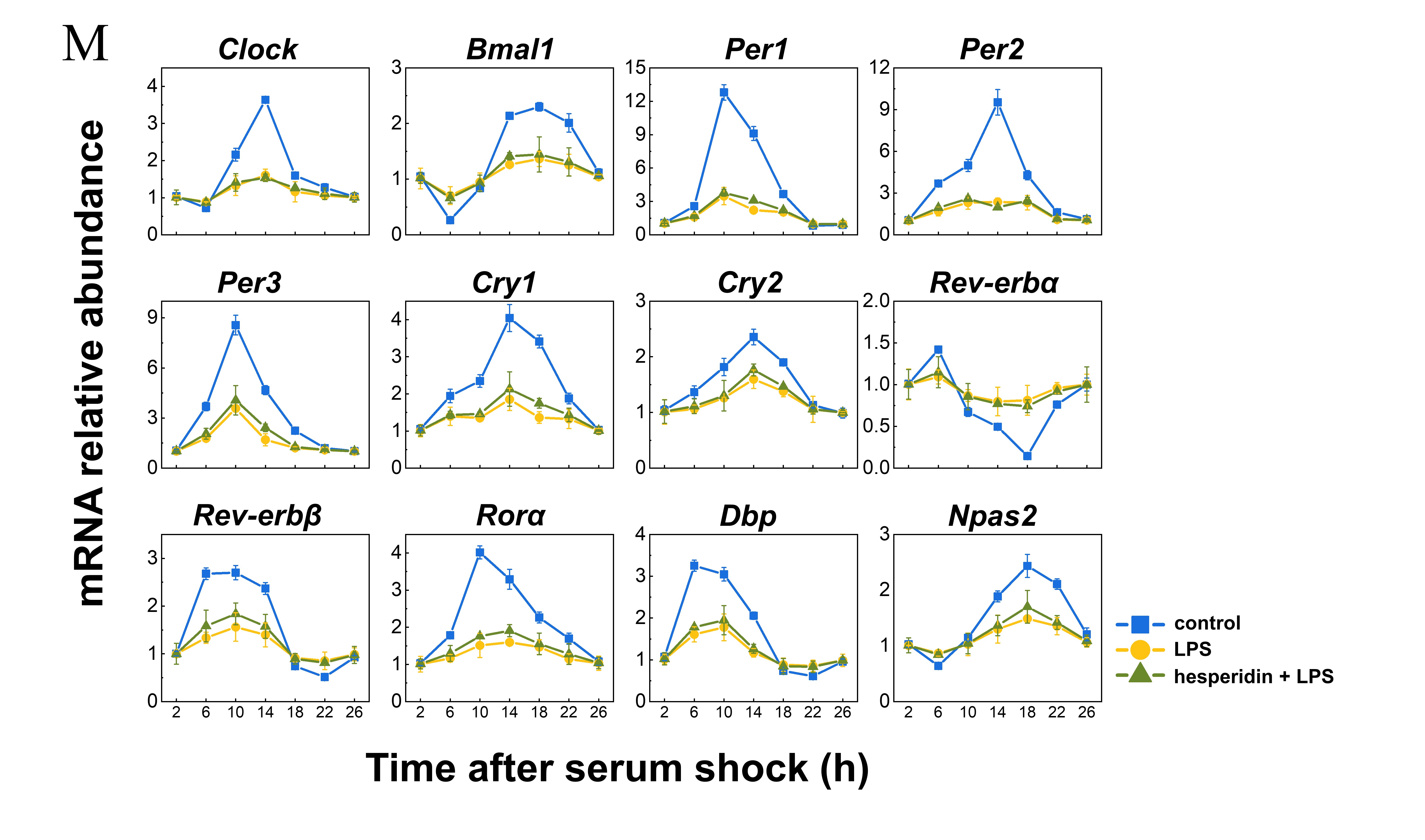

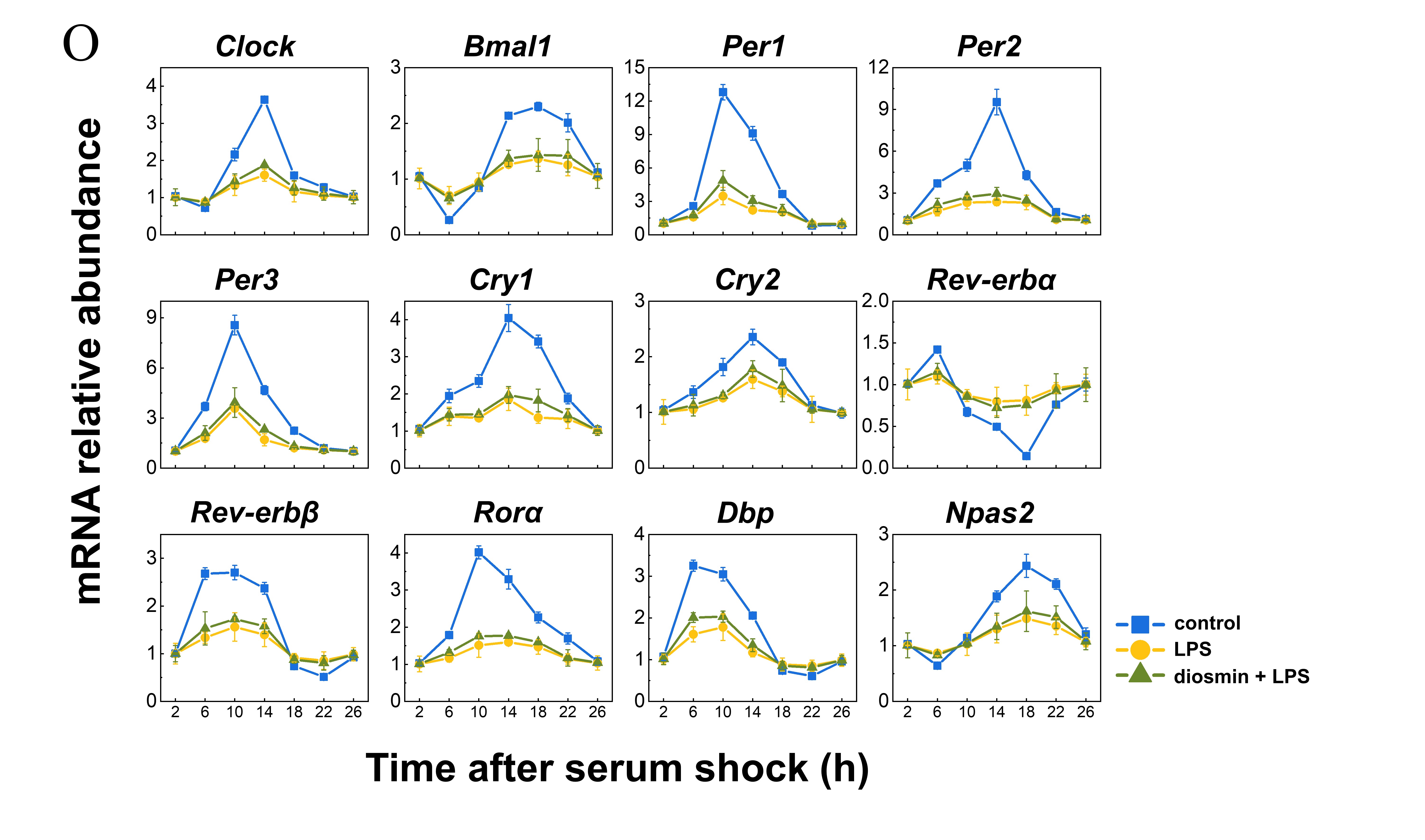

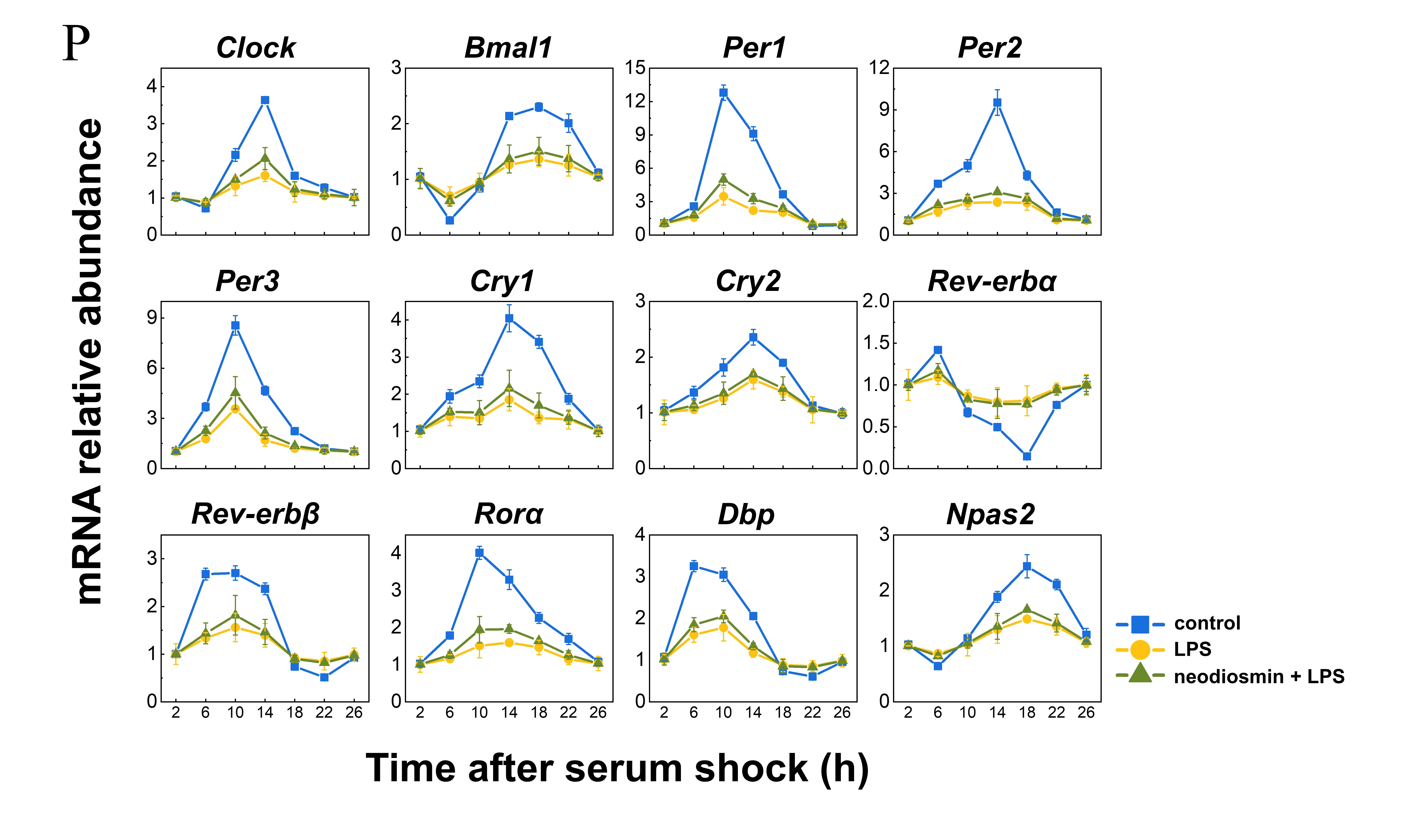

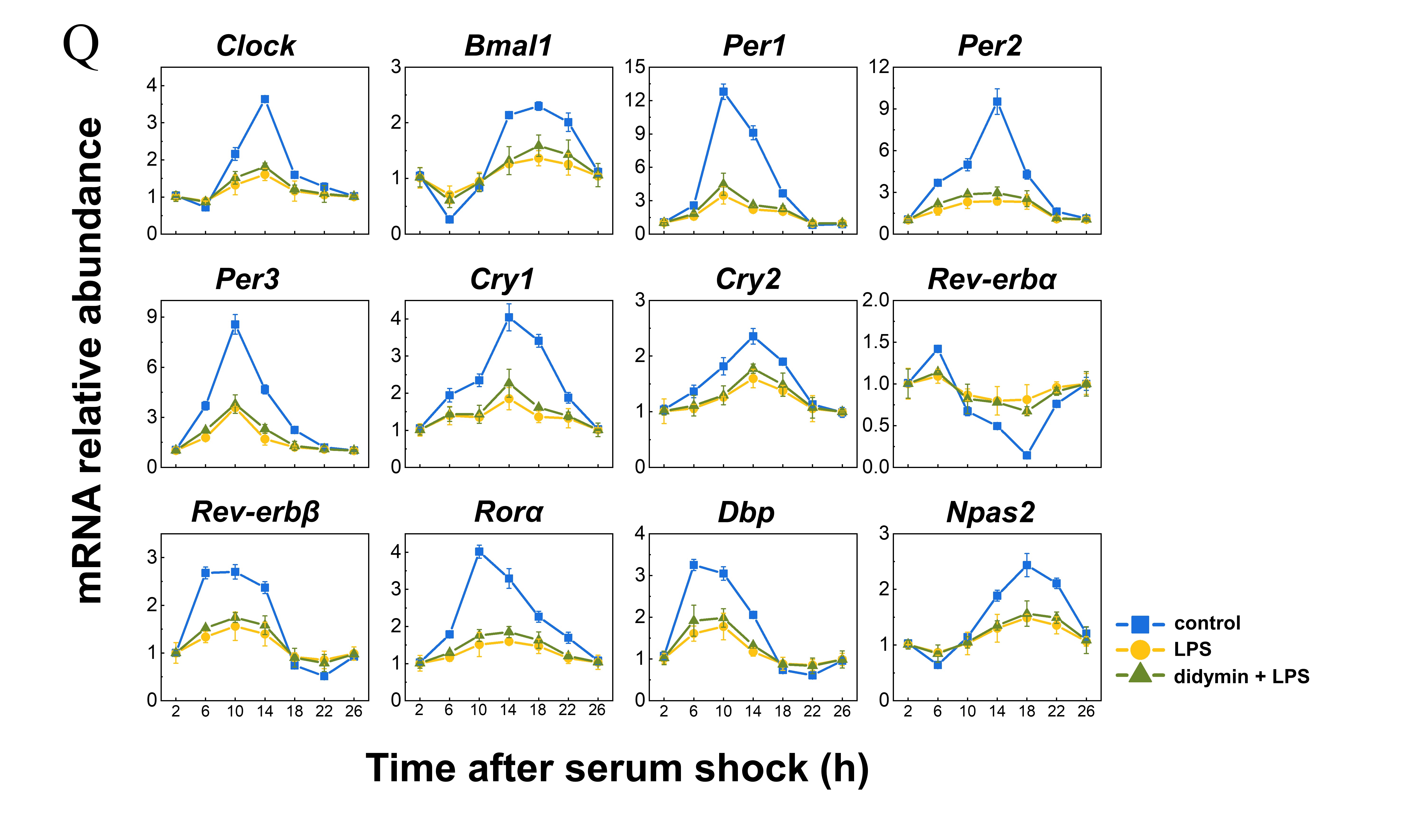

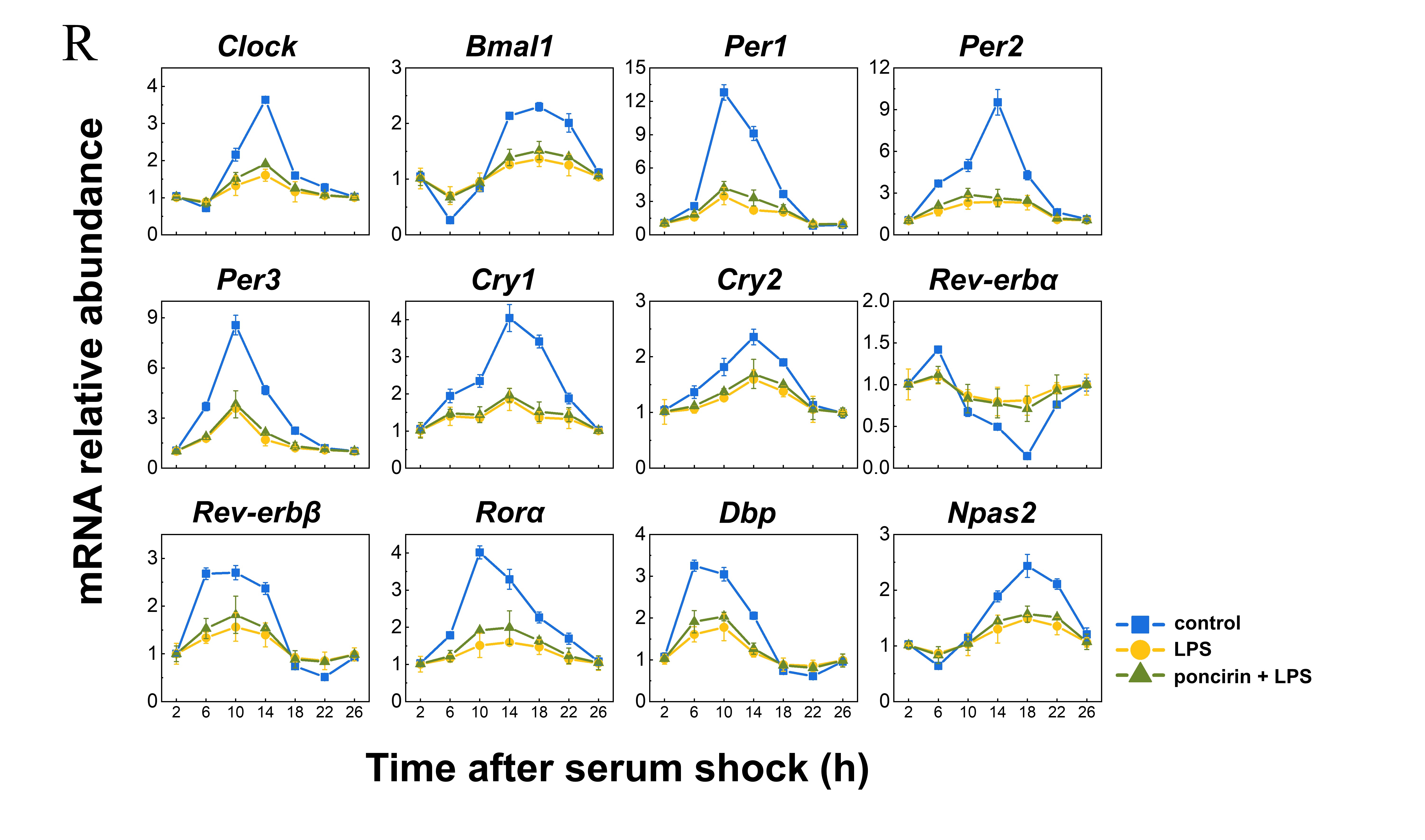

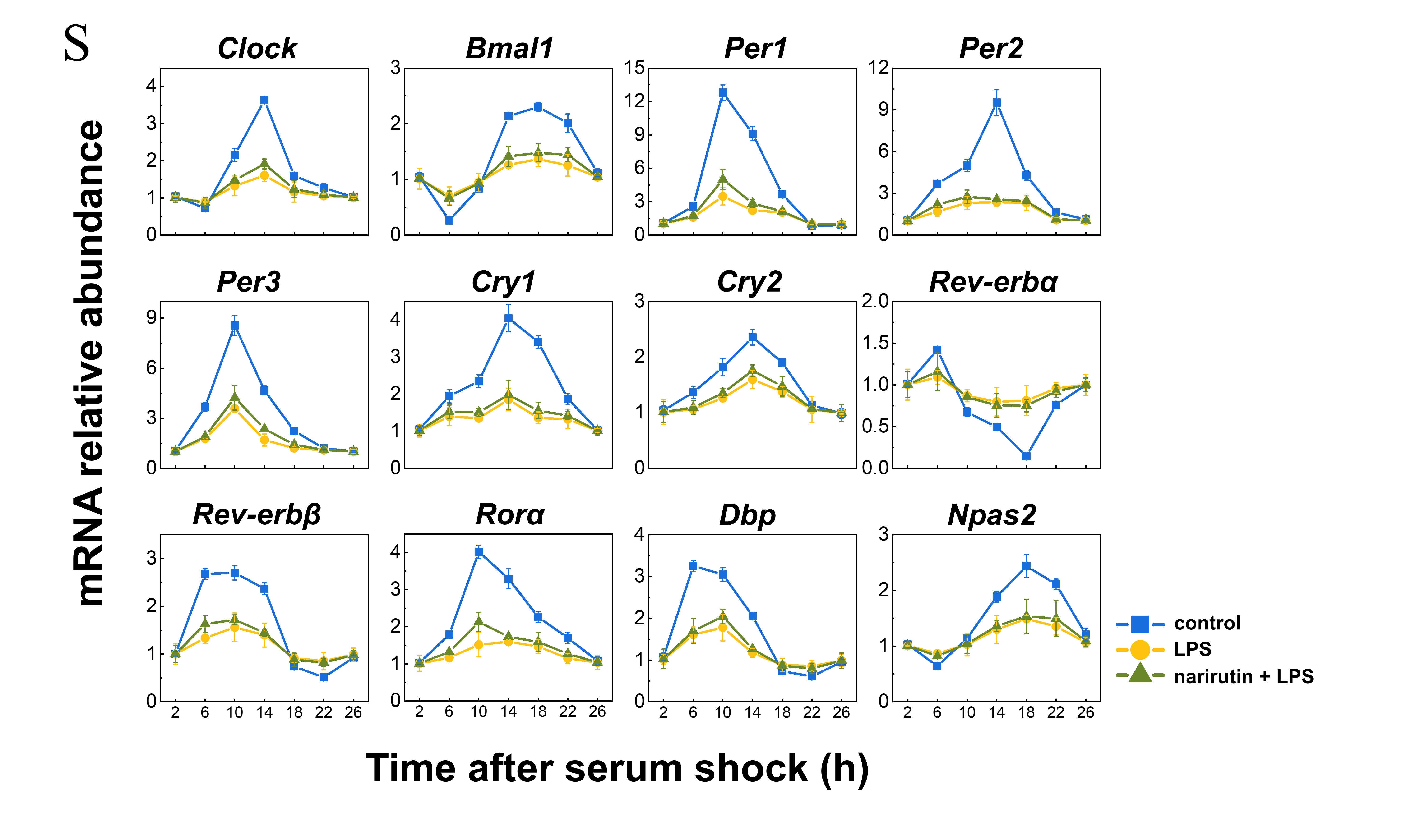

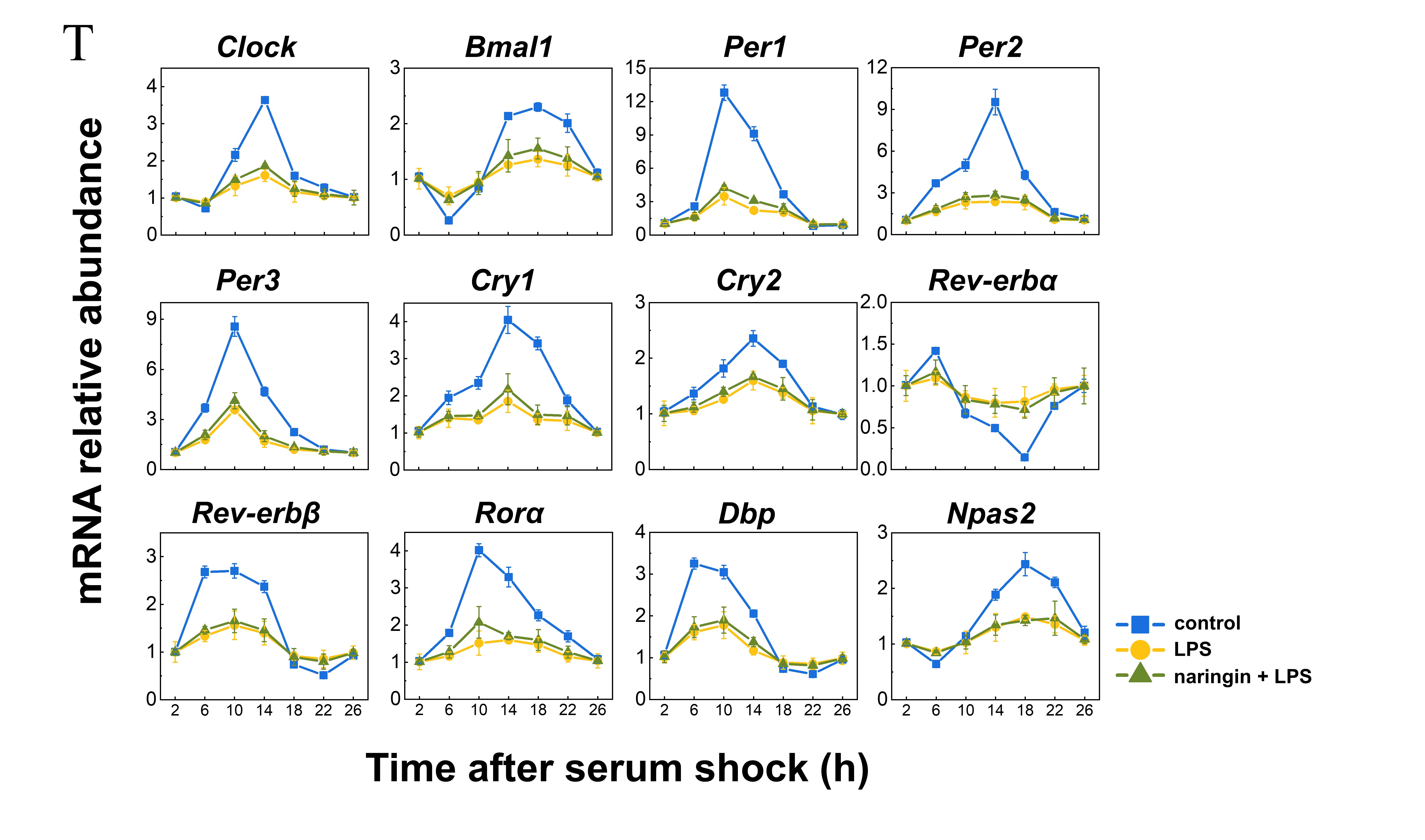

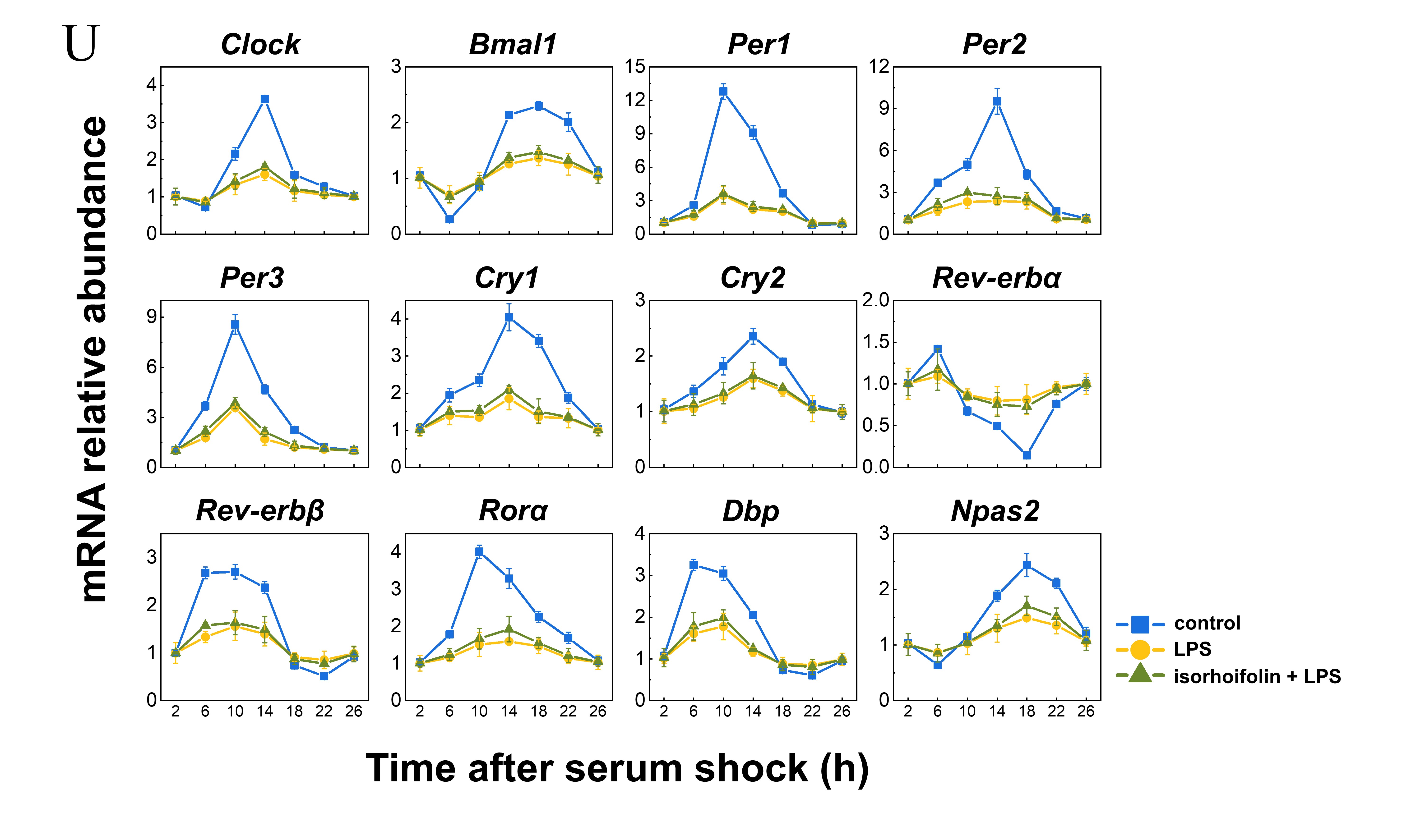

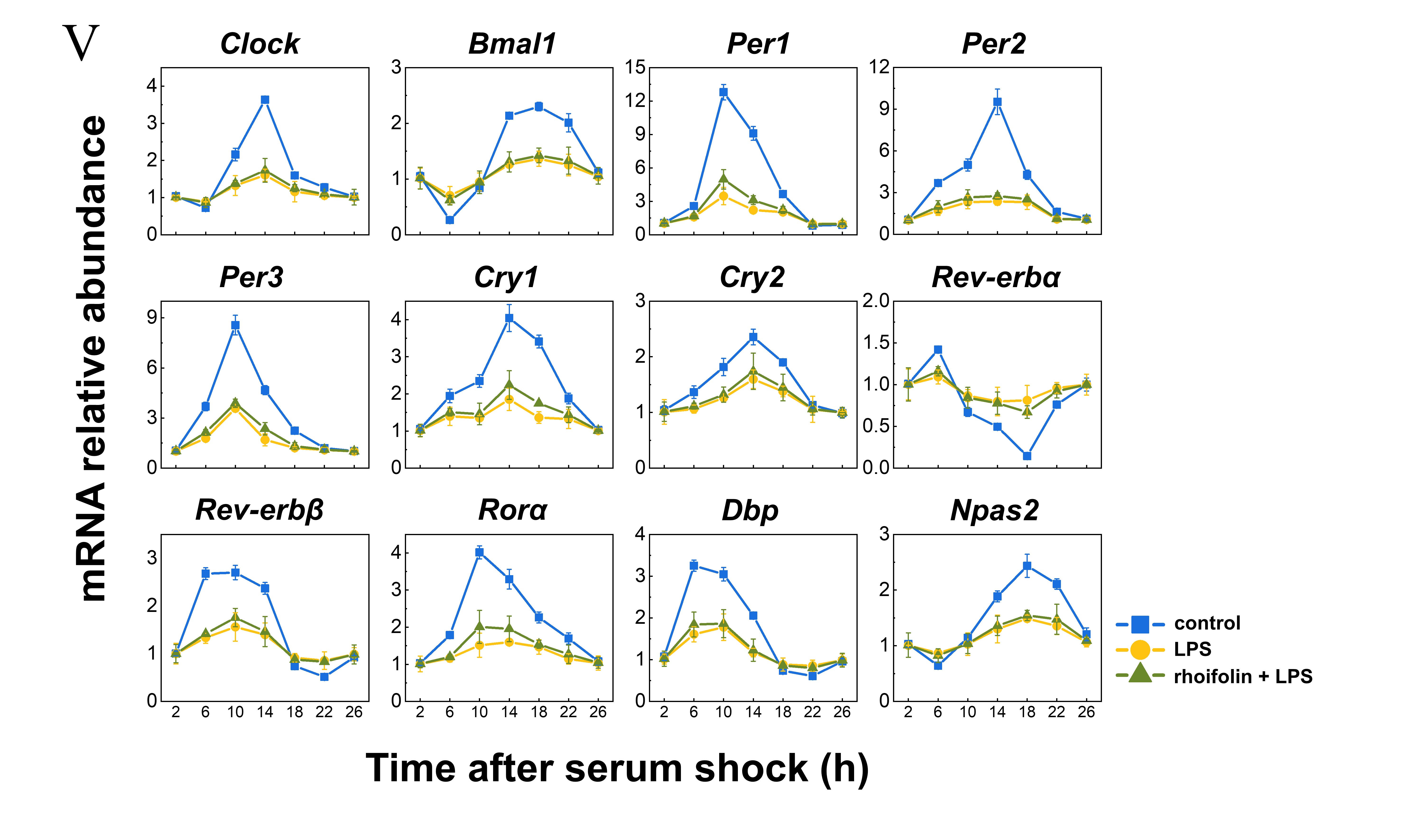

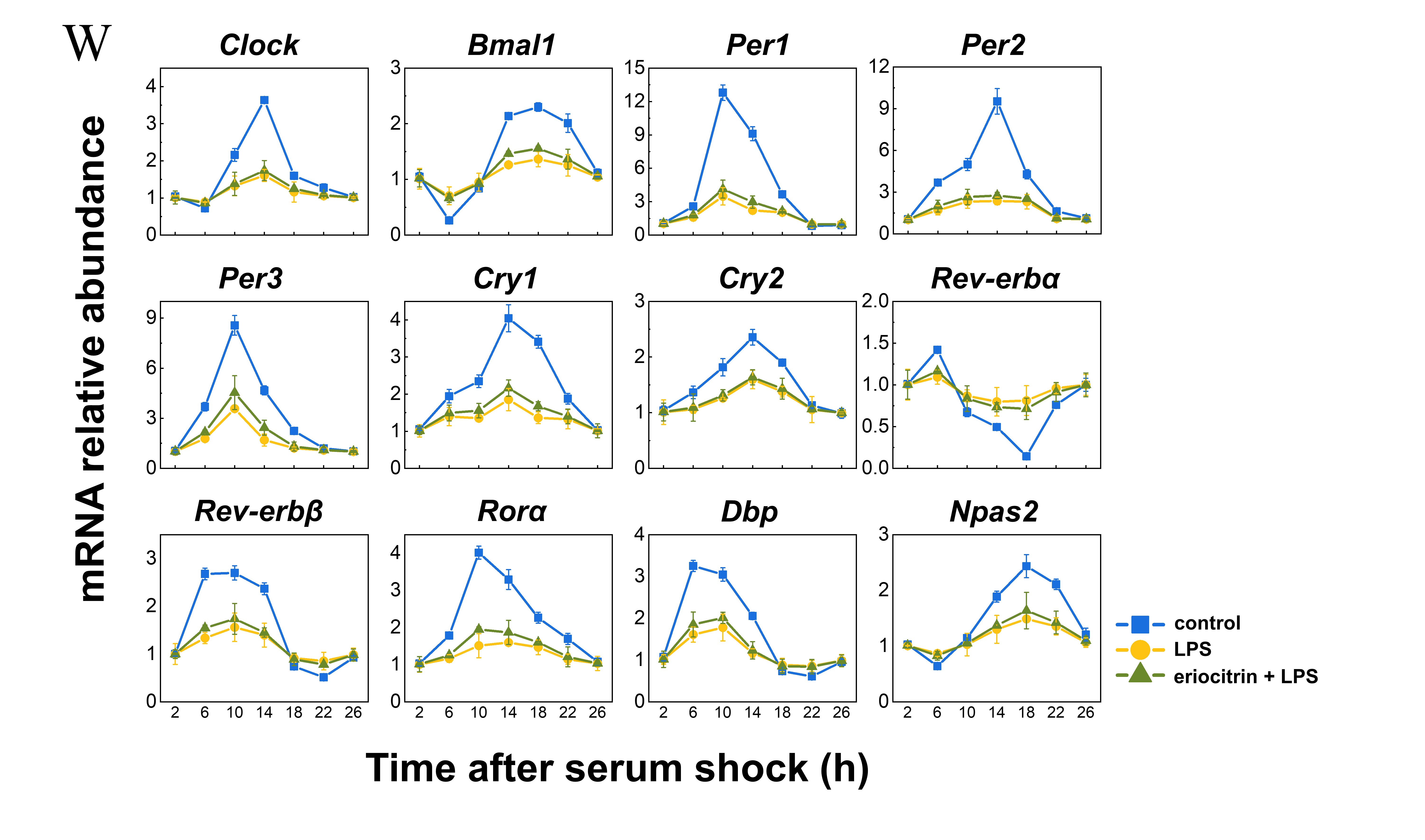

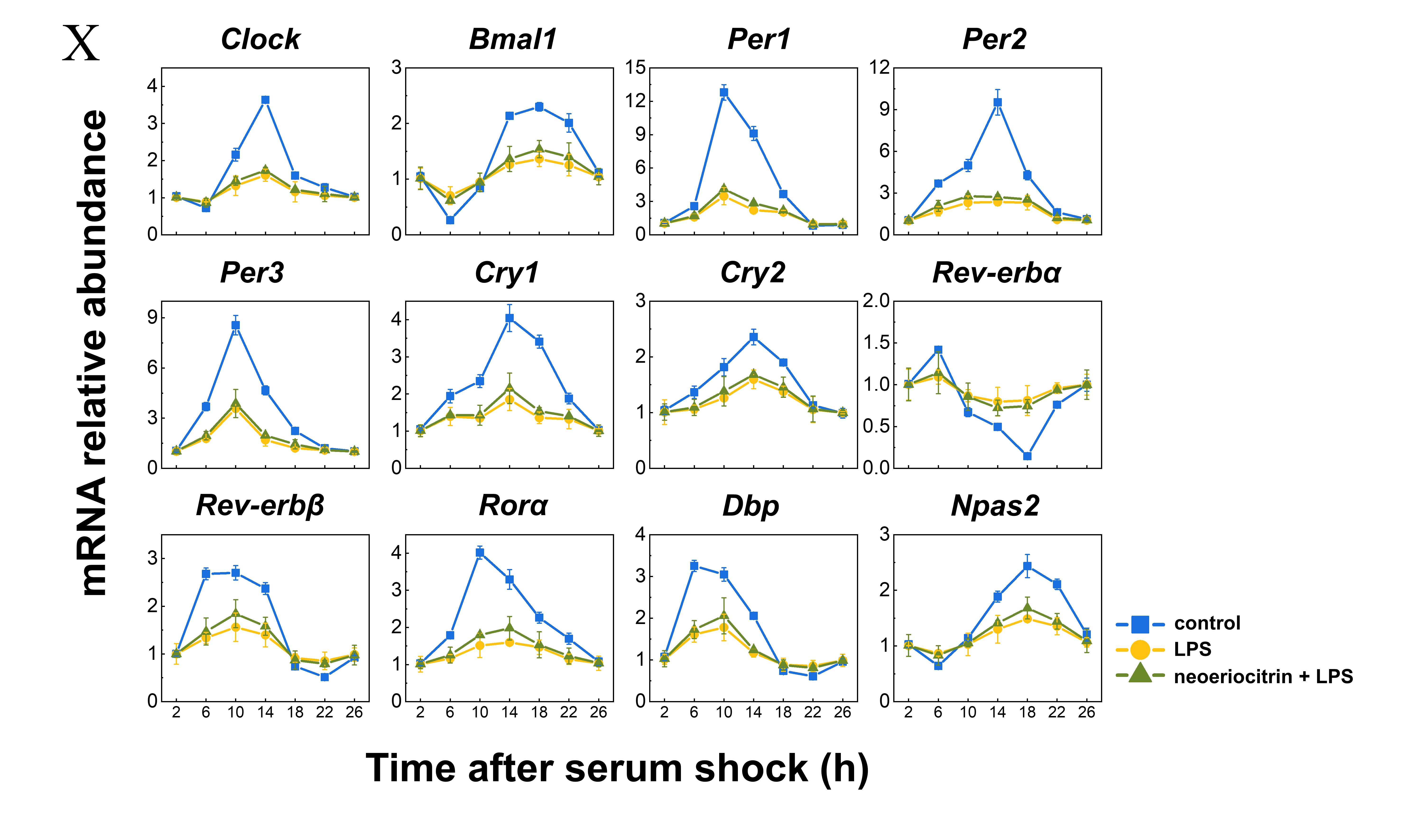


**Figure S3** Effects of flavonoid nobiletin pretreatment on LPS-disturbed expression of circadian genes, *Clock*, *Bmal1*, *Per1*, *Per2*, *Per3*, *Cry1*, *Cry2*, *Rev-erbα*, *Rev-erbβ*, *Rorα*, *Dbp,* and *Npas2*, in BV-2 cells. Relative mRNA levels were determined using qRT-PCR. The pretreatment concentration was: 10 μM for sinensetin, isosinensetin, tangeretin, 5-demethylnobiletin, and gardenin B; 160 μM for diosmetin, hesperetin, agpigenin and naringein, 320 μM for isovitexin, vitexin, vicenin-2, hesperidin, diosmin, didymin, narirutin, isorhoifolin, and eriocitrin, neohesperidin, neodiosmin, poncirin, naringin, rhoifolin, and neoeriocitrin. Data is presented as the mean ± standard deviation (n=3). # *p* < 0.05 of treatments compared to the LPS-induced circadian clock disorder.
